# Systematic Unraveling of the Unsolved Pathway of Nicotine Degradation in *Pseudomonas*

**DOI:** 10.1371/journal.pgen.1003923

**Published:** 2013-10-24

**Authors:** Hongzhi Tang, Lijuan Wang, Weiwei Wang, Hao Yu, Kunzhi Zhang, Yuxiang Yao, Ping Xu

**Affiliations:** State Key Laboratory of Microbial Metabolism, and School of Life Sciences & Biotechnology, Shanghai Jiao Tong University, Shanghai, People's Republic of China; The University of North Carolina at Chapel Hill, United States of America

## Abstract

Microorganisms such as *Pseudomonas putida* play important roles in the mineralization of organic wastes and toxic compounds. To comprehensively and accurately elucidate key processes of nicotine degradation in *Pseudomonas putida*, we measured differential protein abundance levels with MS-based spectral counting in *P. putida* S16 grown on nicotine or glycerol, a non-repressive carbon source. *In silico* analyses highlighted significant clustering of proteins involved in a functional pathway in nicotine degradation. The transcriptional regulation of differentially expressed genes was analyzed by using quantitative reverse transcription-PCR. We observed the following key results: (i) The proteomes, containing 1,292 observed proteins, provide a detailed view of enzymes involved in nicotine metabolism. These proteins could be assigned to the functional groups of transport, detoxification, and amino acid metabolism. There were significant differences in the cytosolic protein patterns of cells growing in a nicotine medium and those in a glycerol medium. (ii) The key step in the conversion of 3-succinoylpyridine to 6-hydroxy-3-succinoylpyridine was catalyzed by a multi-enzyme reaction consisting of a molybdopeterin binding oxidase (*spmA*), molybdopterin dehydrogenase (*spmB*), and a (2Fe-2S)-binding ferredoxin (*spmC*) with molybdenum molybdopterin cytosine dinucleotide as a cofactor. (iii) The gene of a novel nicotine oxidoreductase (*nicA*2) was cloned, and the recombinant protein was characterized. The proteins and functional pathway identified in the current study represent attractive targets for degradation of environmental toxic compounds.

## Author Summary

*Pseudomonas putida* strains are among the microorganisms that have acquired the capability to use toxic and xenobiotic compounds, such as nicotine, for growth. Although nicotine degradation by *Pseudomonas* was first discovered more than 50 years ago, the underlying molecular mechanisms remain unclear. In the last few years, we have made significant efforts to identify the key genes for the hydroxylation of 3-succinoylpyridine (SP) through genomic library screening and purification of wild-type enzymes. However, these efforts did not result in identifying any genes related to SP hydroxylation. In this study, by using comparative genetic analysis, we report the identification of 3 key genes, *spmA*, *spmB* and *spmC* from *P. putida* S16. The heterotrimeric enzyme encoded by these genes requires molybdopterin-cytosine dinucleotide as a cofactor. The proteomes of strain S16 grown on nicotine or glycerol contain 1,292 observed proteins, and provide a detailed view of enzymes involved in nicotine degradation. Our comparative analysis of the proteomic profiles of nicotine grown versus glycerol grown bacterial cells reveals a wide range of cellular processes and functions related to nicotine catabolism.

## Introduction

**Figure 1:**
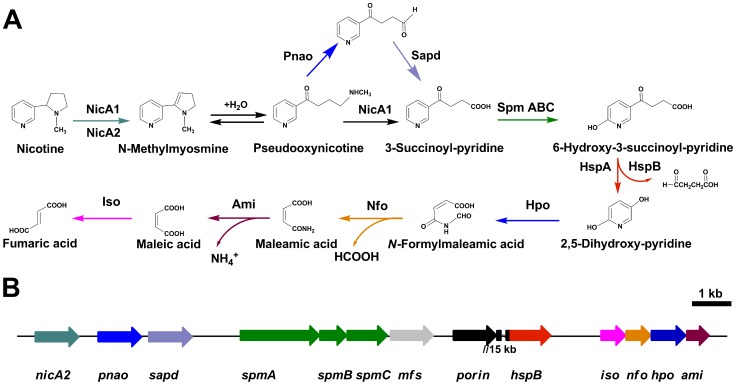
Pyrrolidine pathway of nicotine catabolism in *P. putida* S16. **A**. All complete steps in the catabolism of nicotine by *P. putida* S16. **B**. The genetic organization of a gene cluster involved in nicotine catabolism in *P. putida* S16 were shown. The arrows indicate the size and direction of transcription of each gene, *nicA2*, nicotine oxido-reductase gene; *pnao*, pseudooxynicotine amidase gene; *sapd*, DSP dehydrogenase gene; *spm*, SP monoxygenase gene; *mfs*, major facilitator superfamily gene; *porin*, porin gene; *hspB*, HSP monoxygenase gene; *iso*, maleate isomerase gene; *nfo*, NFM deformylase gene; *hpo*, DHP diooxygenase gene; ami, maleamate amidase gene. Genes are annotated following the color mode indicated. The two genes *porin* and *hspB* are approximate 15 kb apart from each other.

Strains belonging to *Pseudomonas putida*, a non-pathogenic member of the genus *Pseudomonas*, are able to metabolize a variety of organic material [1], [2]. According to their metabolic and physiologic versatility, these strains are thought to play a pivotal role in the recycling of organic wastes in the environment, including soil, fresh water, and the plant rhizosphere [3]. Nicotine is one of the toxic compounds in wastes that accumulate during the processing of tobacco products [4]. Some *Pseudomonas* sp. strains can tolerate high concentrations of nicotine and metabolize nicotine for growth [5]–[9]. *Pseudomonas putida* S16 is one of the bacteria effective in degrading nicotine, and it has been shown to degrade nicotine through the pyrrolidine pathway [9], [10]. The pyrrolidine ring is first dehydrogenated to *N*-methylmyosmine (P), followed by spontaneous hydrolysis to form pseudooxynicotine that is then oxidized to 3-succinoylpyridine (SP) [10], [11]. SP is further hydroxylated to 6-hydroxy-3-succinoylpyridine (HSP), which is converted to fumaric acid in subsequent degradation steps [10], [12] (Figure 1). The HSP hydroxylase (HspA or HspB) converts HSP to 2,5-dihydroxypyridine (DHP) and succinic semialdehyde [12], [13]. *P. putida* S16 transforms DHP through the intermediates, *N*-formylmaleamic acid, maleamic acid and maleic acid, to fumaric acid in later steps of the nicotine degradation pathway (Figure 1). The related four genes, namely 2,5-DHP dioxygenase gene (*hpo*), *N*-formylmaleamate deformylase gene (*nfo*), maleamate amidase gene (*ami*) and maleate *cis-trans* isomerase gene (*iso*) have been cloned and expressed in *Escherichia coli* [14], [15]. Primary annotations for the entire genome of strain S16 have been generated through computational tools such as Glimmer and Blast homology searching [16]. There is, therefore, a need to identify other proteins and pathways capable of degrading nicotine that are not evident from homology modeling, and it should be possible to use a proteomic approach to search for proteins involved in regulatory and molecular mechanisms involving nicotine catabolism.

Despite an increasing awareness of the scientific importance of nicotine degradation, little is known about the key genes encoding for the hydroxylation of SP and the transformation of nicotine in bacteria. In the last few years there have been significant efforts to identify the key genes for the hydroxylation of SP through genome library screening and wild-type enzyme purification. Unfortunately, these efforts did not result in identifying any genes related to SP hydroxylation. Progress is likely to require the development of integrated experimental approaches. With a proteomics approach, it is possible to observe the entire set of protein products synthesized and utilized by an organism [17]. It can help us to accurately determine the boundaries and enumeration of ORFs, and verify unknown ORFs that cannot be well established on the basis of homology.

In this study, we have analyzed the cytosolic protein pattern of strain S16, harboring the essential genes for nicotine degradation, using multi-dimensional liquid chromatography-tandem mass method and molecular genetics. This proteomic information was used to complement gene induction and expression data for a subset of corresponding proteins. Total proteins of 1,292 were identified with the best score (hits> = 5, unique> = 2) by MS/MS spectrum analysis. Proteins involved in transport, energy, detoxification, and stress response were up-regulated in the presence of nicotine. In addition, exposure to nicotine resulted in up-regulation of membrane proteins including porin, outer membrane proteins, and proteins related to solvent efflux pumps. The present study represents an essential approach toward a global molecular characterization of the cellular response in nicotine degradation. We provide evidence for the involvement of SP hydroxylase in the pathway, encoded by a 4.45 kb gene cluster. The enzyme requires the molybdenum molybdopterin cytosine dinucleotide as a cofactor. In addition, we describe another nicotine oxidoreductase (NicA2) that has low amino acid identity (10.9%) to NicA1 (previously called NicA) [11]. Deleting *nicA2* but not *nicA1* prevented nicotine catabolism by *P. putida* S16, demonstrating the importance of the newly discovered NicA2. Four deletion mutants were constructed and showed that these enzymes NicA2, Mfs, Pnao, and SpmABC, are essential for nicotine degradation. To our knowledge, this is the first report of a quantitative study of nicotine-induced changes in global protein and mRNA expression in a *Pseudomonas* strain. Our approach of combining comparative proteomics data with molecular genetics reveals interesting *Pseudomonas*-specific proteins that could be involved in nicotine degradation.

## Results

### Proteomic identification of molecular responses in nicotine degradation of *P. putida* S16 between nicotine and glycerol media

**Figure 2:**
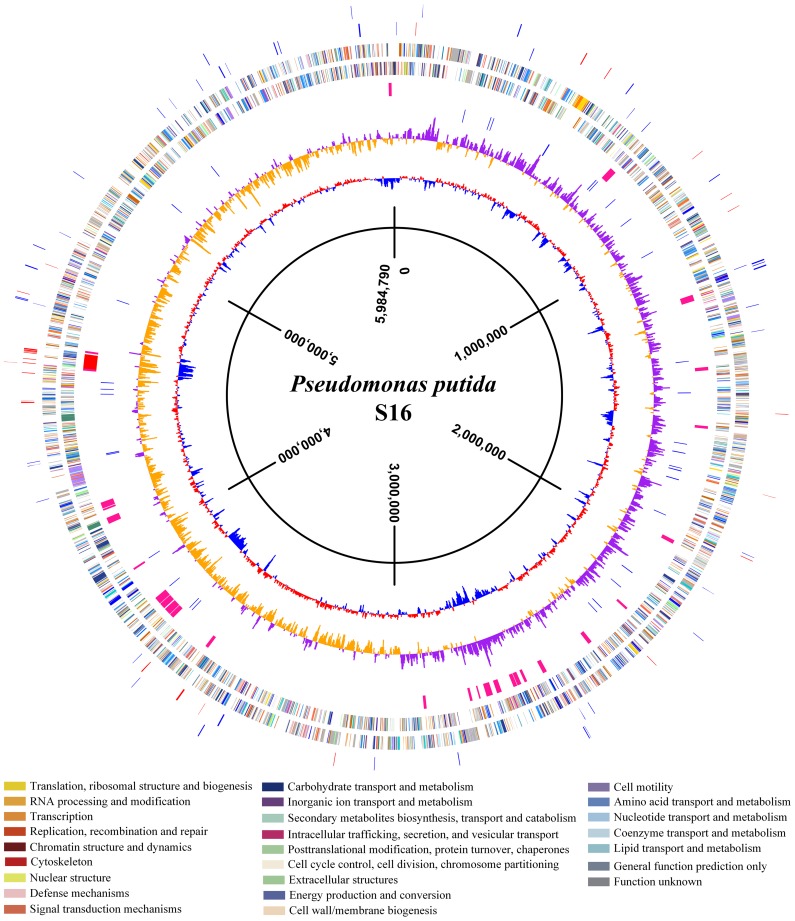
A map of the circular chromosome of *P. putida* S16. The map illustrated the location of known genes, predicted coding regions, genome islands, and differentially expressed proteins. From the outside inwards, circle 1 and circle 2 indicate the protein in the clockwise and anti-clockwise direction that was differentially expressed (> = 3-fold changes and p-values<0.05) in nicotine medium and glycerol medium. The red lines indicate proteins up-expression in nicotine medium, and blue lines indicate proteins up-expression in glycerol medium. Circle 3 and circle 4 indicate the predicted CDSs in the clockwise and anti-clockwise directions, analyzed using the COG database (colors were assigned according to the color code of the COG functional classes); Circle 5 indicates the genome islands predicted in the S16 genome. The red line represents the biggest genome island in S16, in which the nicotine degradation cluster is located in the circle. Circle 6 indicates tRNAs; circle 7 and circle 8 indicate the value of GC skew (G−C/G+C) and percentage of GC content, respectively, with a 4000-bp window size and 2000-bp overlap.

**Figure 3:**
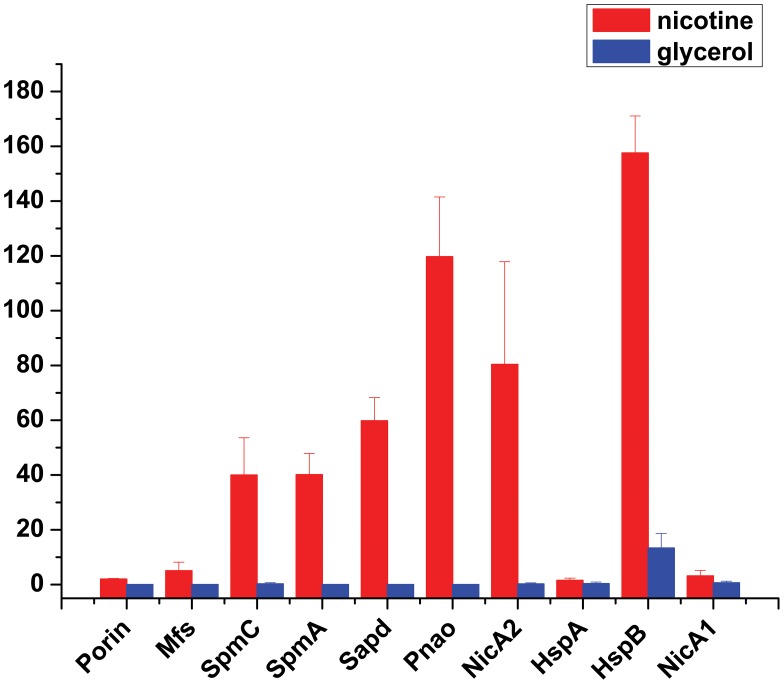
Expression level changes of proteins that were involved in nicotine degradation. The expressions of proteins Porin, Mfs, SpmC, SpmA, Sapd, Pnao, NicA2, HspA, HspB, and NicA1 were identified and detected by MS-based spectral counting. The plot shows expression level of various proteins in different media: red, nicotine medium; blue, glycerol medium. Bars indicate the average ± s.e.m of the normalized spectral counts (y axis).

To uncover molecular pathways in nicotine degradation, we obtained a global overview of protein expression changes between a reference condition (glycerol as the sole carbon source and (NH_4_)_2_SO_4_ as nitrogen source) and a treatment condition (nicotine as the sole carbon and nitrogen source). Direct comparison between the nicotine and glycerol conditions was obtained at the LC-MS/MS measurement stage. Peptides and corresponding proteins were identified and quantitatively analyzed using LTQ Orbitrap XL hybrid FTMS analysis. Three independent runs were measured for each condition (nicotine or glycerol as medium) using a total of six biological samples. Total proteins of 1,292 with a passing score (hits> = 5, unique> = 2) were identified by MS/MS spectrum analysis (Table S1). The 1,292 observed proteins represent 25% of the theoretical proteome (5,218 putative coding sequences of strain S16, [16]). Experimental error was eliminated using global normalization, and a t-test was carried out between the two sets of proteins. Only proteins with> = 3-fold changes and p-values<0.05 were reported as differentially expressed proteins. With these predefined criteria, 126 putative proteins showed a significant change in protein abundance between the nicotine and glycerol mediums (Table S2 and Table S3) (Figure S1 and Figure 2). A map of the circular chromosome of *P. putida* S16, illustrating the location of known genes, predicted coding regions, genome islands, and differentially expressed proteins is provided according to the entire genome and proteomic data of strain S16 [16] (Figure 2). From the outside inwards, circle 1 and circle 2 indicate the proteins in the clockwise and anti-clockwise direction that are differentially expressed (> = 3-fold changes and p-values<0.05) in nicotine and glycerol media. The red lines indicate protein up-expression in nicotine media, and blue lines indicate protein up-expression in glycerol media. Nicotine exposure caused several changes in the abundance of proteins related to carbohydrate metabolism in the proteome of strain S16 (Table S2). NicA2 (PPS_4081), HspB (PPS_4061) (13), Pnao (PPS_4080) [18], and Sapd (PPS_4079) were more abundant in the nicotine condition than in the glycerol condition, suggesting that these enzymes are required for nicotine degradation (Table S2 and Figure 3). Interestingly, the two most likely enzymes (PPS_4077 and PPS_4078) sets for nicotine degradation are significantly up-regulated in the nicotine condition, making them the probable genes responsible for nicotine degradation. The expression of enzyme NicA1 (PPS_0381) in nicotine culture was about 5 times as in glycerol culture. Furthermore, the protein HspA (PPS_0380) was still differently expressed from HspB (PPS_4061) in the nicotine condition as previous reported [13] (Figure 3).

### Effects of nicotine in *P. putida* S16 membrane proteome expression

Proteins other than degradative enzymes that were up-regulated in the presence of nicotine included porin (PPS_4075), major facilitator superfamily metabolite symporter (PPS_4076), TolC family type I secretion outer membrane protein (PPS_3866), the resistance-nodulation-cell division (RND) efflux system outer membrane lipoprotein (PPS_3077), RND family efflux transporter, MFP subunit (PPS_2905), TetR family transcriptional regulator (PPS_1707), and hypothetical proteins (PPS_4370, PPS_3191) (Table S2). Culturing *P. putida* S16 cells in 1 g l^−1^ nicotine resulted in an altered content of several outer membrane proteins, especially in the increased abundance of porin and RND efflux system. The deduced sequence of PPS_4075 indicates it is a putative outer membrane protein porin. The differential expression of the outer membrane porin identified here may relate to adaptation to environmental pressure. The alignment of the sequence indicates that the conserved regions are located on postulated trans-membrane domains in OprD. The RND efflux system outer membrane lipoprotein (PPS_3077), *N*-acetylmuramoyl-L-alanine amidase (PPS_4740), TM helix repeat-containing protein (PPS_1651), and RND family efflux transporter, MFP subunit (PPS_2905) were found to increase following nicotine exposure. The gene PPS_3077 was proposed as an RND efflux transporter outer membrane lipoprotein, which may export toxic organic solvents to the external medium [19]. The content of the subunit was increased after nicotine exposure (Table S2), indicating that this efflux pump is required for strain S16 adaptation to nicotine stress.

### Verification of gene transcriptional expressions

**Figure 4:**
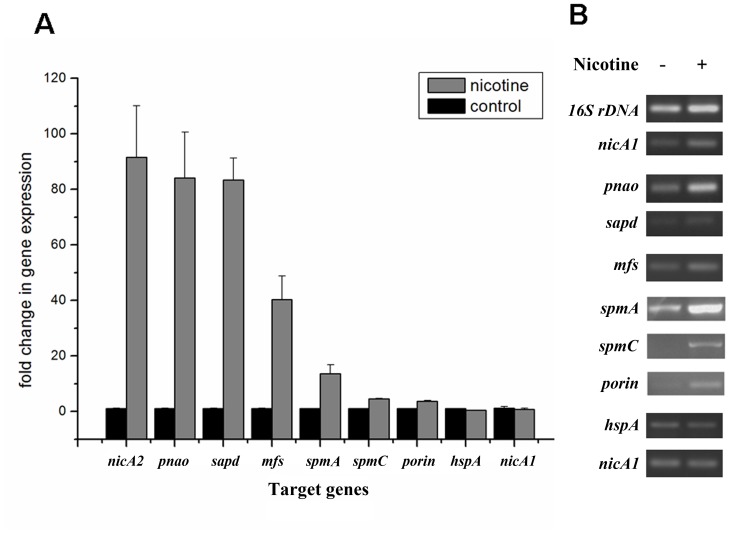
Validation of transcriptional regulation of differentially expressed proteins. RT-qPCR and semi-quantitative RT-PCR analysis of target gene transcripts produced in *P. putida* S16 grown with or without nicotine. **A**. mRNA expression levels of 9 target genes involved in nicotine degradation of *P. putida* S16 were estimated using RT-qPCR and the 2^ΔΔCT^ method. The 16S rRNA gene was used as the reference gene. Results presented in these histograms are the means of three independent experiments, and error bars indicate the standard deviations. **B**. Semi-quantitative RT-PCR analysis of target gene transcripts produced in *P. putida* S16 grown with or without nicotine. The expression of 16 S rDNA was used as an internal control. Primers specific for target genes and 16 S rDNA were used to amplify fragments by RT-PCR.

In order to validate the proteomic data and assess relative transcriptional level of genes related or supposedly related to nicotine degradation in *P. putida* S16, we utilized RT-PCR and RT-qPCR to compare mRNA levels of genes *nicA2*, *pnao*, *sapd*, *mfs*, *spmA*, *spmC*, *porin*, *nicA1*, *and hspA*, supposedly related to nicotine degradation with or without nicotine induction (Figure 4). The RT-qPCR analysis revealed that all target genes appeared to be up-regulated in nicotine-induced *P. putida* S16. The mRNAs of the genes relative to the pathway of nicotine degradation were 18.8- to 90.3- fold more highly expressed in *P. putida* S16 with nicotine relative to non-nicotine induction among which the 90.3-fold upregulated mRNA of *nicA2* was the greatest difference in mRNA levels observed for the tested genes. The mRNAs of *spmA* and *spmC* were also 13.6- and 4.5-fold upregulated, respectively (Figure 4A). Similar results were also observed using semi-quantitative RT-PCR, each of these genes was induced after exposure in nicotine, indicating a role in nicotine degradation of strain S16 (Figure 4B). Overall these data indicate that mRNA levels determined by RT-qPCR were consistent with protein levels measured by 2D LC-MS/MS, and the differentially expressed proteins were regulated at transcriptional level.

### Identification of novel nicotine-induced proteins SpmA, SpmB and SpmC

**Figure 5:**
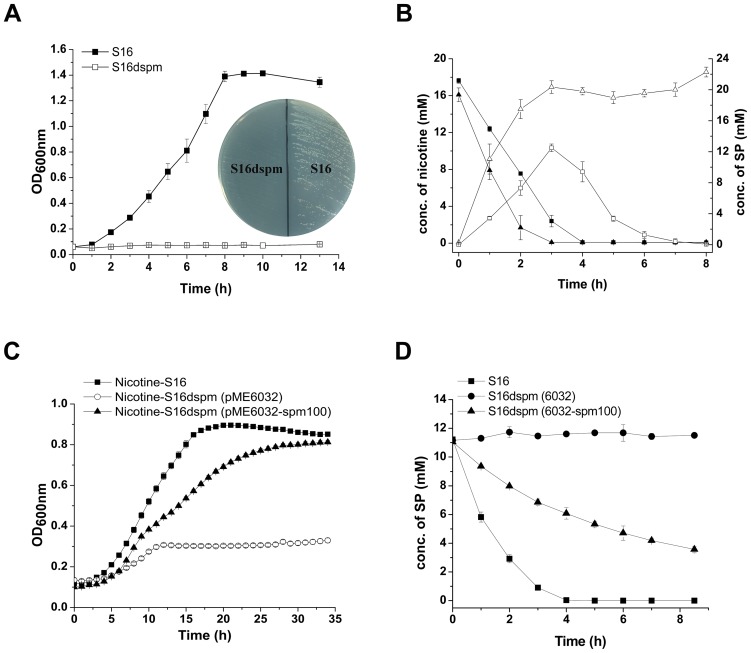
Disruption and complement of *spmABC* genes. **A**. Growth curve of S16 (▪) and the *spmABC* genes disrupted mutant S16d*spm* (□) with nicotine as the sole carbon and nitrogen source. **B**. HPLC analysis of nicotine degradation (solid) and SP formation (open) by resting cells of *P. putida* S16 (square) and *P. putida* S16d*spm* (triangle). Symbol definition: nicotine degradation by S16 (▪solid square), nicotine degradation by S16d*spm* (▴solid triangle), SP formation by S16 (□open square), SP formation by S16d*spm* (▵open triangle). **C**. Growth curves of S16 (▪), S16d*spm* with plasmid pME6032 (○), and S16d*spm* with complementary plasmid pME6032-spm100 were analyzed by Bioscreen C with nicotine as the sole carbon and nitrogen source. **D**. HPLC analysis of SP degradation by resting cells of *P. putida* S16 (▪), *P. putida* S16d*spm* (pME6032) (•), and *P. putida* S16d*spm* (pME6032-spm100) (▴).

The 2D LC-MS/MS and RT-qPCR analysis revealed that the protein and mRNA levels of *spmA* and *spmC* were highly expressed in *P. putida* S16 with nicotine induction, and the fact that the *spmA*, *spmB* and *spmC* gene products show significant sequence identities with the subunits of some members of the xanthine dehydrogenase family (nicotine dehydrogenase of *Arthrobacter nicotinovorans* [20], quinoline 2-oxidoreductase of *P. putida* 86 [21]), suggesting that these three genes encode the three subunits of SP monooxygenase from *P. putida* S16. In order to confirm this hypothesis, the mutant strain *P. putida* S16d*spm* was constructed by polar single homologous recombination in *spmA*, and the related cell growth in nicotine and nicotine conversion by resting cells were both measured. Disruption of the *spmABC* genes (*spmA*, *spmB* and *spmC*) did not allow *P. putida* S16d*spm* to grow with nicotine as the sole carbon and nitrogen source (Figure 5A). Furthermore, resting cells of *P. putida* S16d*spm* converted nicotine to SP completely but could not convert SP anymore, resulting in the accumulation of SP, while those of wide type *P. putida* S16 could catalyze the conversion of SP to HSP (Figure 5B). These data suggest that *spmABC* genes encode the SP monooxygenase that converts SP to HSP. To establish this conclusion more firmly, the fragment *spm*100 including 100 bp upstream from the ATG of *spmA* were cloned and expressed in shuttle plasmid pME6032-spm100. When plasmid pME6032-spm100 was transferred to *P. putida* S16*dspm*, the recombinant strains acquired the ability to grow with nicotine as sole carbon and nitrogen sources (Figure 5C) and also could transform SP to HSP (Figure 5D and Figure S2). These data above indicate that the product of *spmABC* is the monooxygenase responsible for hydroxylation of SP to HSP.

### Functional analysis of *mfs* (PPS_4076) and *sapd* (PPS_4079)

PPS_4076, which encodes the Mfs gene, was knocked out using the pK18mob plasmid. We found that the mutant S16dmfs grew worse than wild type strain S16 (Figure S3). The deletion of *mfs* gene showed that this gene is important for normal nicotine degradation. Interestingly, when the gene *sapd* was knocked out, the mutant S16dsapd grew slower than wild-type strain S16. And both of them could grow well on the nicotine plate (Figure S4).

### Characterization of NicA2 and Pnao

**Figure 6:**
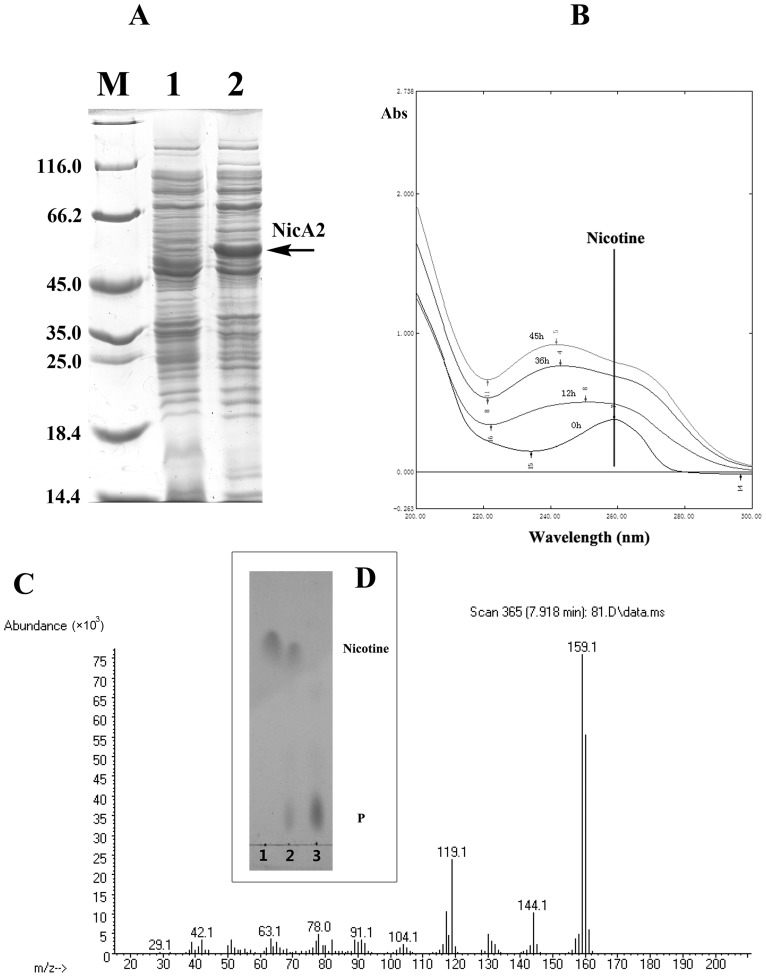
Characterzation of NicA2. **A**. SDS-PAGE analysis of expressed NicA2 in *E. coli* BL21(DE3) on a 12.5% gel. Lane M, protein molecular weight marker (MBI); lane 1, cell extracts of *E. coli* containing plasmid pET-28a; lanes 2, cell extracts of *E. coli* (pET28a-*nicA2*) obtained 4 h after IPTG induction. **B**. UV spectrum for the conversion of nicotine by transformant pET28a-*nicA2*. **C**. Mass spectra of *N*-methylmyosmine (P) as determined by GC-MS analysis. **D**. TLC analysis of the products formed by incubation of nicotine and whole cells of transformant pET28a-*nicA2*. The qualitative analysis of nicotine and *N*-methylmyosmine (P) was performed by using analytical TLC according to published procedures [10], [12]. Nicotine and *N*-methylmyosmine (P) are indicated in the figure.

**Figure 7:**
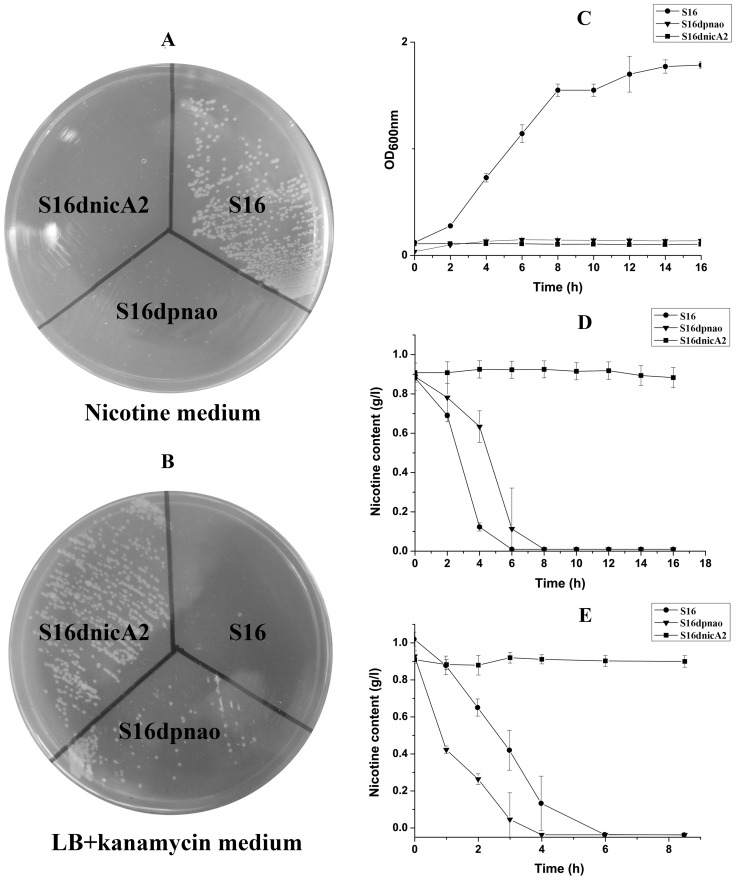
Cell growth and resting cell reactions of strain S16 and the gene deletion mutants. **A**. Strain S16 and gene deletion mutants grown on a nicotine plate containing nicotine as the sole carbon and nitrogen source. **B**. Strain S16 and gene deletion mutants grown on LB plate containing kanamycin. **C**. Growth curves of S16 (•), S16d*pnao* (▾), and S16d-*nicA2* (▪) with nicotine as sole carbon and nitrogen sources. **D**. HPLC analysis of nicotine degradation by cell culture of *P. putida* S16 (•), S16d*pnao* (▾), and S16d*nicA2* (▪). **E**. HPLC analysis of nicotine degradation by resting cells of *P. putida* S16 (•), S16d*pnao* (▾), and S16d*nicA2* (▪). The values are means of three replicates, and the error bars indicate the standard deviations.

To verify the prediction of the novel gene, *nicA2* from strain S16 was cloned and expressed in *E. coli* BL21(DE3) cells (Text S1). After IPTG induction, large amounts of proteins approximately 50 kDa in size were found in the lysate of *E. coli*, whereas no such band could be observed in uninduced cells or in cells containing the vector alone (Figure 6A). Resting cells containing pET28a-*nicA2* plasmids were used to convert nicotine, and they could degrade nicotine as indicated by UV-scan analysis; thus it was confirmed that the *nicA2* gene product possesses the expected functions (Figure 6B). Intermediate *N*-methyl-myosmine (P) was identified by TLC and GC-MS analysis, and we observed the same result as previously reported (Figure 6C and Figure 6D) [11]. His_6_-tagged NicA2 was purified with Ni-NTA affinity columns, and the purity of the enzyme was confirmed by SDS-PAGE, and a single band was observed close to 50 kDa (Figure S5A). Further experimental evidence supported the involvement of FAD in the function of NicA2. UVvisible maximum absorbance at 382 and 452 nm with a minimum at 410 nm is characteristic of a flavoprotein (Figure S5B). The *nicA2* and *pnao* genes were successfully deleted separately (Figure 7A and Figure 7B), and the related cell growth and resting cell reactions were all performed (Text S1). The *nicA2* and *pnao* gene deletion mutant could not grow in the nicotine medium plate and liquid cultures (Figure 7A and Figure 7C). However, the deletion mutant S16dpnao could degrade nicotine well in the LB medium containing 1 g l^−1^ nicotine, at a rate comparable to wild type strain S16 (Figure 7D). Resting cells of the *nicA2* gene deletion mutant could not degrade nicotine, in contrast to strain S16 (Figure 7E). The *pnao* gene deletion mutant could grow in LB medium containing 1 g l^−1^ nicotine and turn its color to deep yellow, whereas the color of the culture medium did not change for the *nicA2* deletion mutant. In addition, resting cells of *pnao* gene deletion mutant degraded nicotine, but it could not further degrade *N*-methylmyosime and use nicotine as the sole carbon and nitrogen source (Figures 7A, 7C and 7E). The above facts indicate that enzymes NicA2 and Pnao are crucial for nicotine degradation by the strain.

## Discussion

Investigations into the functional genomics of the gram-negative *Pseudomonas putida* have revealed valuable insights into basic concepts of cell physiology [3]. *Pseudomonas putida* are among the microorganisms that can utilize any of a variety of organic compounds as a sole carbon and nitrogen source. Many of these have acquired the capability to use toxic and xenobiotic substances for growth. They play key roles in the elimination of organic wastes in polluted water and soils [1], [22]. Recently the degradation of nicotine by such microorganisms has received increased attention for its great potential in detoxifying tobacco wastes [5], [6], [23].

**Figure 8:**
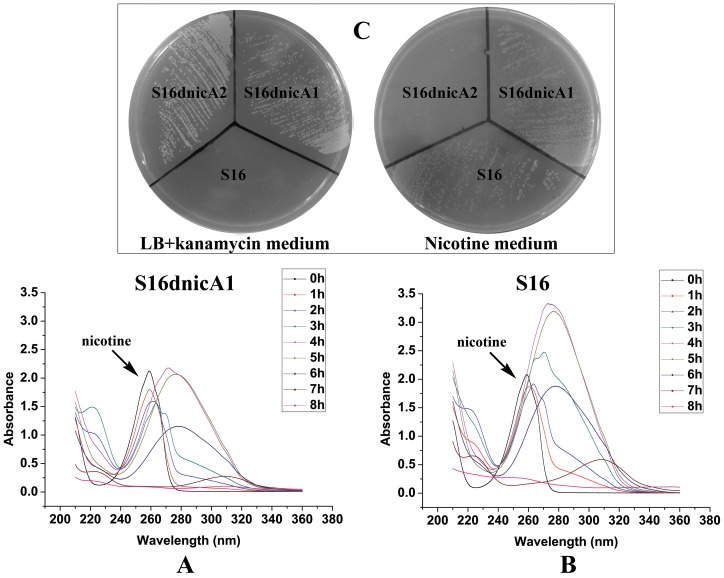
Cell growth and resting cell reactions of strain S16 and the gene deletion mutant S16d*nicA1*. **A**. UV-scan analysis of nicotine degradation by resting cell of gene deletion mutant S16d*nicA1*. **B**. UV-scan analysis of nicotine degradation by resting cell of strain S16. **C**. Strain S16, gene deletion mutant S16d*nicA1*, and gene deletion mutant S16d*nicA2* grown on a nicotine plate containing nicotine as the sole carbon and nitrogen source, and on a LB plate containing kanamycin.

To better understand molecular pathways responding to nicotine, we studied the proteomes of *P. putida* S16 cultures with either nicotine or glycerol as the sole carbon source. The proteomes of strain S16 contain 1,292 observed proteins, and provide a detailed view of the enzymes involved in nicotine degradation (Table S1). By combining the data obtained from reverse transcription-PCR, RT-qPCR, mutant construction, and the proteome of strain S16, we previously identified a genome island of more than 10 nicotine-induced genes (Figures 1, 2, 3, and 4). In our previous work, a *nic* gene cluster encoding nicotine oxidoreductase (NicA) (recently named as NicA1) and HSP hydroxylase (HspA) from *P. putida* S16 has been characterized [11], [12]. In this study, the gene for a novel enzyme nicotine oxidoreductase (*nicA2*), which converts nicotine to *N*-methylmyosmine, was identified from a new genomic island and it showed low amino acid identity (10.8%) to NicA1 [11]. Isoenzymes are present in bacteria such as the *hspA* and *hspB* genes, and they are located in different gene clusters [13]. We propose that these two enzymes may be isoenzymes coexisting in the same cell. The *nicA1* and *nicA2* genes were deleted separately, and the related cell growth and resting cell reactions were all performed (Figure 7 and Figure 8). The *nicA1* gene deletion mutant could grow well in nicotine medium as the same as wild-type strain S16 (Figures 8A, 8B, and 8C), whereas the *nicA2* gene deletion mutant could not grow in nicotine medium (Figure 7 and Figure 8C). Deleting *nicA2* but not *nicA1* prevented nicotine catabolism, demonstrating the importance of the newly discovered NicA2. A homology search of the NCBI databases reveals that the amino acid sequences of NicA2 share 23.6%, 12.0%, and 82.0% identities with the amino acid sequences of 6-hydroxy-l-nicotine oxidase from *Arthrobacter nicotinovorans*, 6-hydroxy-d-nicotine oxidase from *Arthrobacter nicotinovorans*, and nicotine oxidase from *Pseudomonas* sp. strain HZN6, respectively [5], [24]. Collectively these 10 nicotine-induced genes help to clearly understand the unknown catabolic pathway involved in nicotine degradation by *P. putida* S16. Comparative analysis of the proteomic profiles generated in this study reveals a wide range of cellular processes and functions, with some of most profound changes in expression occurring among annotated functional proteins with roles in amino acid transport, energy production, cell wall membrane and envelope biogenesis such as the genes *porin* and *mfs* (Table S2, Figure S1 and Figure 2). The successful application of this protocol leads to the extension and refinement for the global response of *P. putida* to nicotine.

In the last few years there have been significant efforts to identify the key genes for the hydroxylation of SP through different methods without success. Finally in this study, with the help of comparative proteome and genetic analysis, the genes *spmA, B and C* of *P. putida* were newly identified and characterized. These encode 3-succinoyl-pyridine monooxygenase, which hydroxylates SP to HSP as a step in nicotine degradation. The amino acid sequences of all parts of Spm show significant homology to various prokaryotic molybdenum containing hydroxylases and eukaryotic xanthine dehydrogenases. Expression of the *spmA*, *B*, and *C* genes and formation of catalytically active SP monooxygenase was achieved in recombinant *P. putida* S16d*spm*. Attempts to detect the active enzyme in the corresponding *E. coli* BL21(DE3) possessing the recombinant plasmid pET28a-*spmABC* were not successful, a result similar to previous work examining the genes *qorM, S*, and *L* involved in quinoline degradation by *P. putida* 86 [25] and another result similar to pAO1-encoded nicotine dehydrogenease genes in nicotine degradation by *Arthrobacter nicotinovorans* [26]. When the Spm genes are expressed in *E. coli*, it may result in formation of inactive 3-succinoyl-pyridine monooxygenase that lacks the molybdenum molybdopterin cytosine dinucleotide cofactor (Mo-MCD) [21], [25], [26]. In *E. coli*, the cofactor is molybdopterin guanine dinucleotide (MGD) as the organic part of the molybdenum cofactor, while other bacteria make use of Mo-MCD cofactor [27], [28]. Therefore, it is unable to integrate the Mo-MCD cofactor into the functional apoprotein. This explains why we failed to obtain the *spm* genes using several other methods over the past few years, such as construction of a genomic library, cloning genes and expression in *E. coli*. The observations above suggest that Spm is a novel Mo-MCD containing three-subunit hydroxylases involved in nicotine degradation by *Pseudomonas* sp. To our knowledge, *sirA2* (243 bp) in *Pseudomonas* sp. HZN6 is the only reported gene related to SP degradation. SirA2, 80 amino acid residues, is a kind of sulfur-transferase, which is required for formation of the molybtopterin cofactor and for a functional hydroxylase of the pyridine ring [29]. However, Spm in *P. putida* S16 is responsible for hydroxylation of SP to HSP, and Mo-MCD is crucial for Spm in *P. putida* S16. Our present study helps to illuminate the whole nicotine degradation pathway by *P. putida* S16 and further the understanding of nicotine's metabolic mechanism at the molecular level in *Pseudomonas*.

All these mechanistic insights may be extended to *Pseudomonas* adaptation to environments, with a possible impact in biodegradation, bioremediation and biocatalysts. The availability of the 2D LC/MS data presented here will allow a more targeted search for proteins that are differentially expressed during a particular cellular activity. Examining the proteome and the corresponding transcriptional data is a useful method for understanding the molecular changes involved in the catabolism of environmental pollutants and toxicants.

## Materials and Methods

### Chemicals

l-(-)-Nicotine (99% purity) was purchased from Fluka Chemie GmbH (Buchs Corp., Switzerland). Sequencing grade trypsin was from Promega (Madison, Sweden). SP (98%) was obtained from Toronto (Canada). All other reagents were of analytical grade and commercially available.

### Bacterial strains, culture conditions and assays

*P. putida* S16 was cultured with 1 g l^−1^ nicotine as carbon and nitrogen source as described previously [9]. The bacterium was also cultivated in 1 g l^−1^ glycerol, 1 g l^−1^ (NH_4_)_2_SO_4_ and a mineral salts medium with an initial pH of 7.0 and containing 13.3 g l^−1^ K_2_HPO_4_ 3 H_2_O, 4 g l^−1^ KH_2_PO_4_, 0.2 g l^−1^ MgSO_4_ 7 H_2_O, and 0.5 mg trace elements solution. The trace elements solution contained (per liter of 0.1 M HCl) the following materials and quantities: 0.05 g CaCl_2_ 2 H_2_O, 0.05 g CuCl_2_ 2 H_2_O, 0.008 g MnSO_4_ H_2_O, 0.004 g FeSO_4_ 7 H_2_O, 0.1 g ZnSO_4_, 0.1 g Na_2_MoO_4_ 2 H_2_O, and 0.05 g Na_2_WO_4_ 2 H_2_O. Quantitative data of nicotine, SP and HSP were obtained by high-performance liquid chromatography (HPLC) analysis according to the previous reports [11], [12].

### Preparation of proteomes for HPLC-MS/MS analysis

Cells of *P. putida* S16 grown on nicotine or glycerol as the carbon source were separately suspended in PBS buffer. After washing with PBS buffer, cells were lysed by resuspending them in buffer (8 M urea, 0.05% SDS, 10 mM DTT, 10 mM Tris, pH 8.0) with liquid nitrogen grinding. After centrifugation at 12,000 *g* (10 min, at 4°C), the supernatant was mixed with acetone (precooling) according to the volume ration of 1∶4. Following overnight incubation at −20°C, the mixture was centrifuged at 12,000 *g* (10 min, at 4°C). The pellet was washed with precooling acetone three times, and suspended in buffer containing 6 M Gu-HCl, 100 mM Tris, pH 8.3. The protein content was determined using a modified Bradford protocol. The enzyme solution (100 µg) was suspended in buffer containing 10 mM DTT at 56°C for 0.5 h, and 50 mM IAA added at 25°C for 40 min. After 3 K ultrafiltration membrane ultrafiltration and a flush of the membrane with 100 mM NH_4_HCO_3_, the pH of the solution was adjusted 8.0–8.5. 40 µg of sequencing-grade modified trypsin was added to the extract and digestion was carried out overnight at 37°C with gentle rotation (protein : trypsin ration = 50 : 1).

### 2D-LC/MS and protein identification

In order to identify proteins from the cell mixtures, multi-dimensional liquid chromatography in proteomics using an 1100 LC system were used. The first dimension starts with the elution peptides from a silica strong cation exchange column (0.075 mm×5 cm) and C18 column (0.075 mm×10 cm) (Column Technology Inc.) with a real continuous linear salt gradient (0–130 min, 2%–35%; 130–135 min, 35%–90%; 135–140 min, 90%; 140–141 min, 90%–2%; 141–180 min, 2%). Chromatography conditions: buffer A: H_2_O; buffer B: acetonitrile. The nanospray column was directly interfaced to the orifice of an LTQ Classic ion trap mass spectrometer (Thermo Fisher). Nanospray ionization was accomplished with a spray voltage of 3.5 kV and heated capillary temperature of 200°C. The *m*/*z* range was from 400 to 1800.

### Proteome bioinformatics

Databases searches for MS or MS/MS spectra were conducted using proteomics discovery software 1.2 (ThermoFisher, CA, USA). Mass spectra were collected throughout the entire chromatograph run. Mass spectra were analyzed by Bioworks. High scoring peptide matches were automatically identified and organized. A protein database from the annotated *P. putida* S16 genome containing a total of protein entries was used. The peptide match with an assumed charge state of Z = 1 and an XCorr score of >2.2, or charge state of Z = 3 and an XCorr score of >3.75 was automatically accepted as valid.

### RNA extraction and real time quantitative reverse transcription PCR (RT-qPCR)

A single colony of *P. putida* S16 was randomly picked from the minimal medium plate, and a 1 : 100 dilution of a fresh overnight culture was inoculated with 50 ml minimal medium (control) and minimal medium with 1 g l^−1^ nicotine added (induction) in three 250 ml flasks. Batch cultures were incubated at 30°C with shaking (200 rpm) to mid-exponential phase (OD_600_ 1.4). The mid-exponential cells were harvested by 14,000 g centrifugation for 2 min with the pellets stored at −70°C overnight (16 h). Total RNAs were extracted from ∼1×10^9^ cells of *P. putida* S16 with an RNAprep pure cell/bacteria kit (Tiangen), and quantified by NanoVue (GE Healthcare). 0.8 mg of DNase (Fermentas) treated total RNA was reverse transcribed to cDNA using random hexamer primers and SuperScript III reverse transcriptase (Invitrogen). The cDNA was diluted 1 : 10 and served as the template for qPCR analysis using the CFX96 Real-Time PCR Detection system (Bio-Rad) with SYBR Green RealMasterMix (Tiangen) and qPCR primers (Table S4). Melting curves and agarose gel analyses were used to confirm the specificity of the PCR products. The standard curve for each primer pair was constructed with a tenfold dilution of *P. putida* S16 genomic DNA. Experiments were performed routinely with control and nicotine induction cultures of *P. putida* S16 in triplicates, and the threshold cycle (C_T_) values for each target gene was normalized to the reference gene, 16S rRNA gene. The 2^ΔΔCT^ method was used to calculate the relative expression level, where ΔΔC_T_ = (C_T, target_−C_T_, _16S_)_induction_−(C_T, target_−C_T_, _16S_)_control_ [30].

### Construction of the *spmABC* genes disrupted mutant *P. putida* S16d*spm*

A fragment of *spm*A carrying 5′- and 3′- truncations was amplified from *P. putida* S16 total DNA by PCR, and cloned into the polylinker of pK18mob, a mobilizable plasmid that does not replicate in *Pseudomonas*. PCR primer sequences are as follows: *spmA*-SalI, CCACGTCGACCAAGTTAACTGGTTATGCGAC and *spmA*-EcoRI, CCACGAATTCAGTCCTTGGCCGAAACTTTGC. Recombinant plasmids were transferred from the broad-host-range mobilizing strain *E. coli* S17-1 to *P. putida* S16 by biparental filter mating [31]. Donors and recipients were grown to OD_600nm_ 0.6 in LB broths at 37°C and 30°C, respectively. The 10^9^ donor cells and 2×10^9^ recipient cells were washed with 0.85% NaCl three times, mixed and spread on 0.45-µm filters placed on LB agar. The plates were incubated face up for 5 h at 37°C, and 12 h at 30°C. The cells were then resuspended in 0.5 ml 0.85% NaCl and plated at different solutions on M9 plates containing 100 mg l^−1^ kanamycin and incubated at 30°C. Kan^r^ transconjugants were tested by PCR analysis with the primer pair *spmA*-SalI, and pK18mob-269, GCTTCCCAACCTTACCAGAG.

### Construction of the plasmid pME6032-spm100 containing complementary *spmABC* genes

The 4.45 kb fragment containing the coding region of the *spmABC* genes and 100 bp upstream from the ATG of *spmA* was amplified from the total DNA of *P. putida* S16 with the primers in , and then cloned into pME6032 [32] generating recombinant plasmid pME6032-spm100.

### Construction of the genes *mfs*, *sapd, pnao, nicA2* disrupted mutants of *P. putida* S16

DNA fragments of *mfs*, *sapd*, *pnao* and *nicA2* were amplified from total DNA of *P. putida* S16 by PCR, and cloned into the polylinker of pK18mob. PCR primer pair sequences are as shown in Table S6. *P. putida* S16 was transformed by electroporation according to the following conditions: 0.5–1 µg DNA was added to 100 µl electrocompetent cells of *P. putida* S16, and the mixture was electroporated at 12 kV cm^−1^, 200 Ω, 25 µF with a Bio-Rad Gene-Pulser Xcell (Bio-Rad Laboratories, Hercules, CA).

## Supporting Information

Figure S1 Functional category distribution of up-regulated proteins identified from strain S16 cells grown in the medium containing nicotine as the sole carbon and nitrogen source.
(TIF)

Figure S2 HPLC analysis of trasformation of SP to HSP (for 3-h reaction) by resting cells. Resting cells are from cultures of *P. putida* S16, *P. putida* S16d*spm* (pME6032), and *P. putida* S16d*spm* (pME6032-spm100).
(TIF)

Figure S3 Cell growth of strain S16 and the gene deletion mutant. **A**. Strain S16 and gene deletion mutants grown on LB plate containing kanamycin. **B**. Strain S16 and gene deletion mutant grown on a nicotine plate containing nicotine as the sole carbon and nitrogen source. **C**. Growth curves of S16 and S16d*pps4076* with nicotine as sole carbon and nitrogen sources. The values are means of three replicates, and the error bars indicate the standard deviations.
(TIF)

Figure S4 Cell growth of strain S16 and the gene deletion mutant. **A**. Strain S16 and gene deletion mutant S16-d*pps4079* grown on LB plate containing kanamycin. **B**. Strain S16 and gene deletion mutant S16-d*pps4079* grown on a nicotine plate containing nicotine as the sole carbon and nitrogen source. **C**. Growth curves of strains S16 and S16d*pps4079* with nicotine as sole carbon and nitrogen sources. The values are means of three replicates, and the error bars indicate the standard deviations.
(TIF)

Figure S5 Purification and characterization of NicA2. **A**. SDS-PAGE. *Lane M*, marker proteins; *lane Sup*, supernatant of strain S16 cell extract; *lane Pre*, precipitant of strain S16 cell extract; *lane FT*, flute throw after purification by Ni-NTA affinity columns; *lane E1*, elution by 20 mM imidazole using Ni-NTA affinity column; *lane* E2, elution by 50 mM imidazole using Ni-NTA affinity column. The molecular masses of markers (in kilodaltons) are indicated on the left. The molecular mass of the purified protein is about 50 kDa. **B**. UV-scan analysis of purified protein NicA2. The red line means for FAD from the boiled NicA2 solution. The black line means for FAD from the unboiled NicA2 solution.
(TIF)

Table S1 Comparative analysis of the proteomics of *Pseudomonas putida* S16 in nicotine and glycerol mediums.
(DOCX)

Table S2 Abundance of differential expression protein components involved in central energy metabolism in *P. putida* S16 cells grown on nicotine and glycerol.
(DOCX)

Table S3 Abundance of differential expression protein components involved in central energy metabolism in *P. putida* S16 cells grown on nicotine and glycerol.
(DOC)

Table S4 Primers used for gene expression analysis (RT-qPCR).
(DOC)

Table S5 The *spm* genes and their products in *P. putida* S16.
(DOC)

Table S6 Primers of genes knocked out.
(DOCX)

Text S1 Supplementary materials and methods.
(DOCX)
