# Supplementary figures and images for: Systematic Unraveling of the Unsolved Pathway of Nicotine Degradation in Pseudomonas
Source: PLoS Genet. 2013 Oct 24;9(10):e1003923. doi: 10.1371/journal.pgen.1003923 (PMC3812094; doi:10.1371/journal.pgen.1003923)

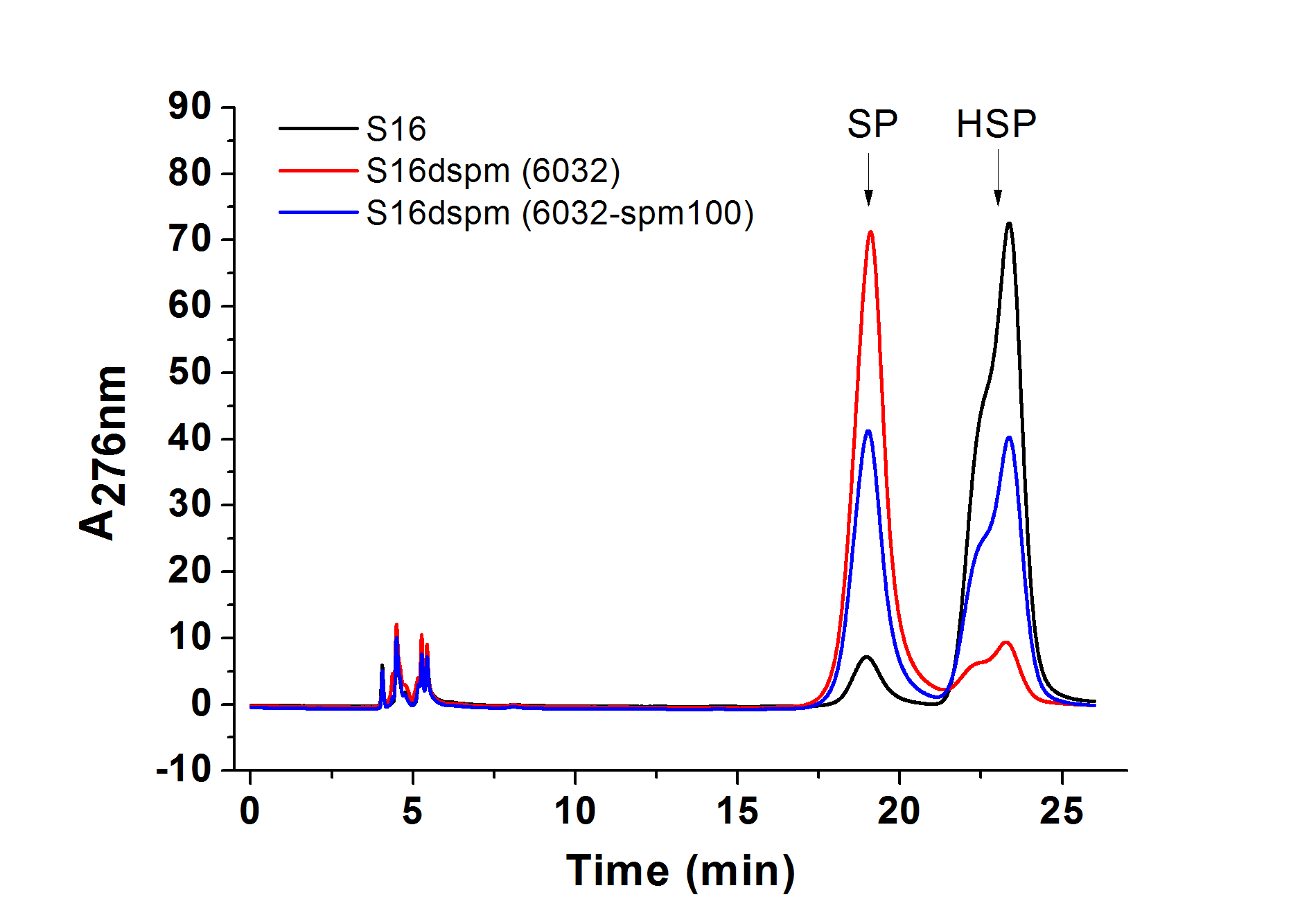

Supplement: Figure S2 — HPLC analysis of trasformation of SP to HSP (for 3-h reaction) by resting cells. Resting cells are from cultures of P. putida S16, P. putida S16dspm (pME6032), and P. putida S16dspm (pME6032-spm100). (TIF) [file pgen.1003923.s002.tif]

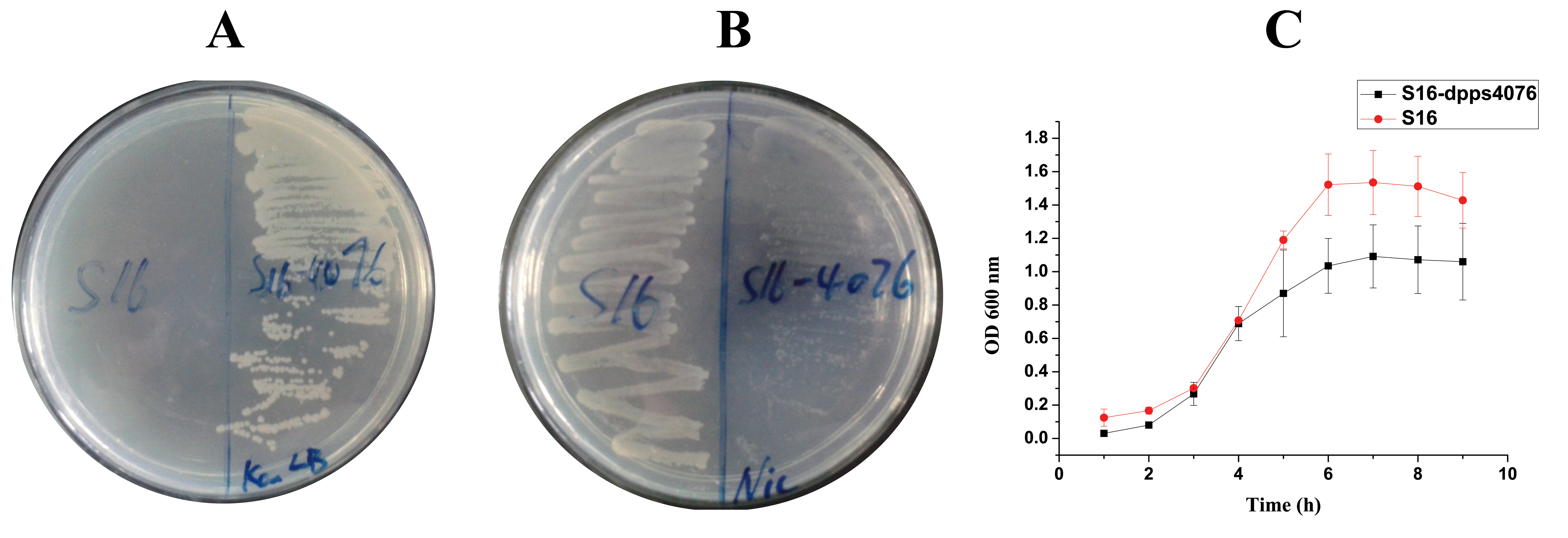

Supplement: Figure S3 — Cell growth of strain S16 and the gene deletion mutant. A. Strain S16 and gene deletion mutants grown on LB plate containing kanamycin. B. Strain S16 and gene deletion mutant grown on a nicotine plate containing nicotine as the sole carbon and nitrogen source. C. Growth curves of S16 and S16dpps4076 with nicotine as sole carbon and nitrogen sources. The values are means of three replicates, and the error bars indicate the standard deviations. (TIF) [file pgen.1003923.s003.tif]

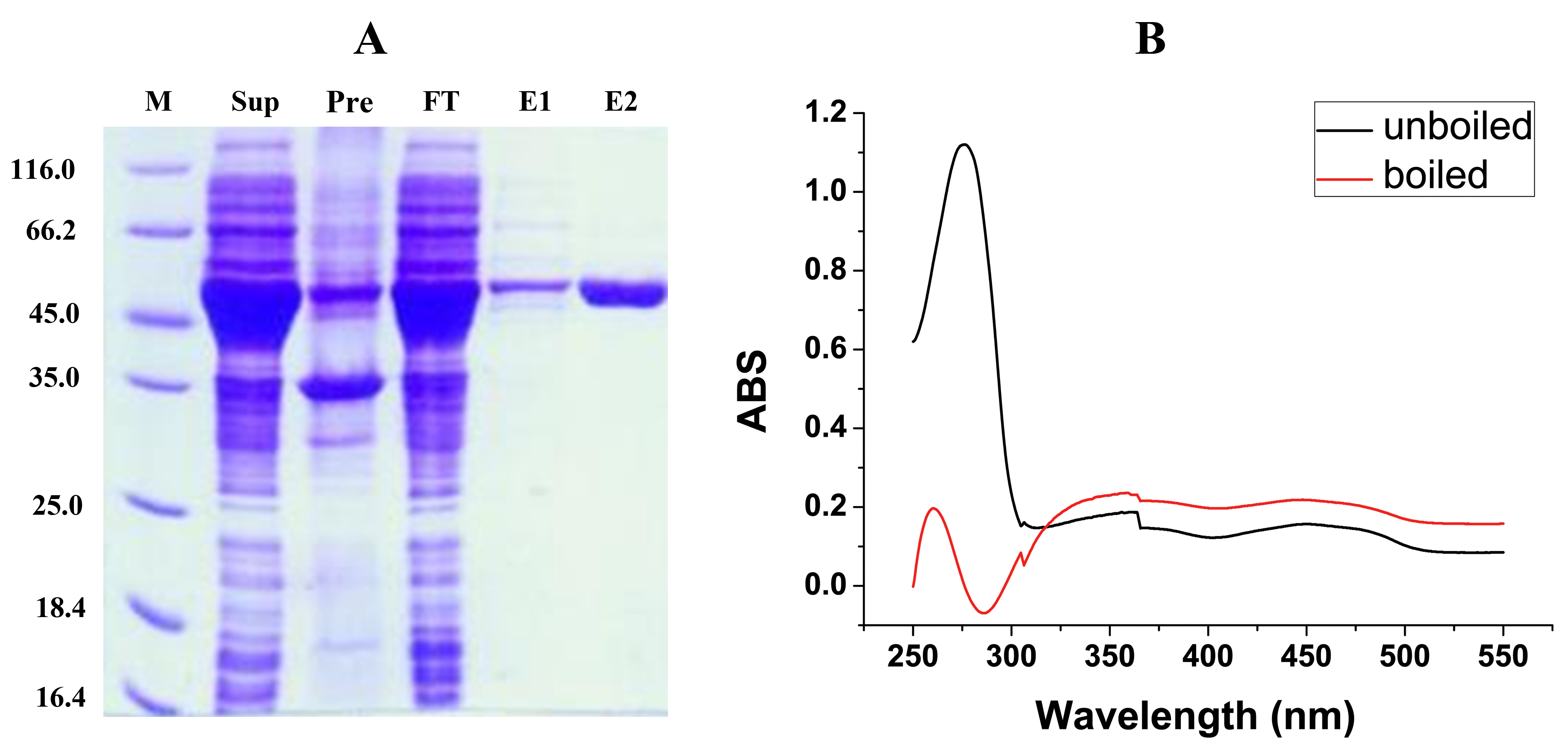

Supplement: Figure S5 — Purification and characterization of NicA2. A. SDS-PAGE. Lane M, marker proteins; lane Sup, supernatant of strain S16 cell extract; lane Pre, precipitant of strain S16 cell extract; lane FT, flute throw after purification by Ni-NTA affinity columns; lane E1, elution by 20 mM imidazole using Ni-NTA affinity column; lane E2, elution by 50 mM imidazole using Ni-NTA affinity column. The molecular masses of markers (in kilodaltons) are indicated on the left. The molecular mass of the purified protein is about 50 kDa. B. UV-scan analysis of purified protein NicA2. The red line means for FAD from the boiled NicA2 solution. The black line means for FAD from the unboiled NicA2 solution. (TIF) [file pgen.1003923.s005.tif]
