# Supplementary material for: Systematic Unraveling of the Unsolved Pathway of Nicotine Degradation in Pseudomonas
Source: PLoS Genet. 2013 Oct 24;9(10):e1003923. doi: 10.1371/journal.pgen.1003923 (PMC3812094; doi:10.1371/journal.pgen.1003923)
Supplement: Table S1 — Comparative analysis of the proteomics of Pseudomonas putida S16 in nicotine and glycerol mediums. (DOCX) [file pgen.1003923.s006.docx]

Table S1 Comparative analysis of the proteomics of *Pseudomonas putida* S16 in nicotine and glycerol mediums.

| References | KEG analysis | gs16_1 | gs16_2 | gs16_3 | ns16_1 | ns16_2 | ns16_3 | Comparison of Multiple protein Expression | Hits | UniquePep | P-value |
| --- | --- | --- | --- | --- | --- | --- | --- | --- | --- | --- | --- |
| PPS_1707 | Transcription | 0 | 0 | 0 | 3.12525 | 3.468165 | 1.113377 | #DIV/0! | 6 | 3 | 0.072916 |
| PPS_4075 |  | 0 | 0 | 0 | 2.0835 | 1.734082 | 2.226755 | #DIV/0! | 5 | 2 | 0.005232 |
| PPS_4076 | Carbohydrate transport and metabolism | 0 | 0 | 0 | 3.12525 | 8.670412 | 3.340132 | #DIV/0! | 11 | 2 | 0.108578 |
| PPS_4078 | Energy production and conversion | 0 | 0 | 0 | 48.96225 | 34.68165 | 36.74145 | #DIV/0! | 100 | 24 | 0.012111 |
| PPS_4079 | Energy production and conversion | 0 | 0 | 0 | 66.672 | 50.28839 | 62.34913 | #DIV/0! | 149 | 15 | 0.00666 |
| PPS_4080 | Amino acid transport and metabolism | 0 | 0 | 0 | 140.6363 | 97.10861 | 121.3581 | #DIV/0! | 300 | 19 | 0.010887 |
| PPS_1542 | Cell division and chromosome partitioning | 0 | 0 | 0 | 1.04175 | 1.734082 | 3.340132 | #DIV/0! | 5 | 2 | 0.095753 |
| PPS_3077 | Cell envelope biogenesis | 0 | 0 | 0 | 4.167 | 3.468165 | 1.113377 | #DIV/0! | 7 | 6 | 0.08738 |
| PPS_1628 | Amino acid transport and metabolism | 0 | 0 | 0 | 3.12525 | 3.468165 | 0 | #DIV/0! | 5 | 2 | 0.184605 |
| PPS_0750 | Energy production and conversion | 0 | 0 | 0 | 4.167 | 3.468165 | 2.226755 | #DIV/0! | 8 | 2 | 0.028519 |
| PPS_0749 | Energy production and conversion | 0 | 0 | 0 | 9.37575 | 6.93633 | 3.340132 | #DIV/0! | 16 | 6 | 0.064733 |
| PPS_3898 | Lipid metabolism | 0 | 0 | 0 | 4.167 | 0 | 3.340132 | #DIV/0! | 7 | 2 | 0.188412 |
| PPS_4740 | Cell envelope biogenesis | 0 | 0 | 0 | 1.04175 | 3.468165 | 3.340132 | #DIV/0! | 6 | 3 | 0.080021 |
| PPS_3778 | Cell motility and secretion | 0 | 0 | 0 | 3.12525 | 0 | 4.453509 | #DIV/0! | 7 | 6 | 0.195762 |
| PPS_4370 | General function prediction only | 0 | 0 | 0 | 1.04175 | 3.468165 | 2.226755 | #DIV/0! | 5 | 3 | 0.08508 |
| PPS_3191 | Amino acid transport and metabolism | 0 | 0 | 0 | 3.12525 | 3.468165 | 2.226755 | #DIV/0! | 7 | 4 | 0.015482 |
| PPS_3192 | Energy production and conversion | 0 | 0 | 0 | 2.0835 | 3.468165 | 2.226755 | #DIV/0! | 6 | 4 | 0.027566 |
| PPS_3194 | Lipid metabolism | 0 | 0 | 0 | 4.167 | 0 | 3.340132 | #DIV/0! | 7 | 3 | 0.188412 |
| PPS_3960 | Coenzyme metabolism | 0 | 0 | 0 | 11.45925 | 10.40449 | 7.793641 | #DIV/0! | 24 | 5 | 0.011929 |
| PPS_1564 | Defense mechanisms | 0 | 0 | 0 | 3.12525 | 0 | 3.340132 | #DIV/0! | 6 | 3 | 0.183954 |
| PPS_4047 | Intracellular trafficking and secretion | 0 | 0 | 0 | 6.2505 | 6.93633 | 8.907018 | #DIV/0! | 18 | 4 | 0.011487 |
| PPS_4050 | Posttranslational modification | 0 | 0 | 0 | 7.29225 | 6.93633 | 10.0204 | #DIV/0! | 20 | 10 | 0.014215 |
| PPS_4052 | Posttranslational modification | 0 | 0 | 0 | 2.0835 | 10.40449 | 4.453509 | #DIV/0! | 12 | 3 | 0.150035 |
| PPS_4053 | Posttranslational modification | 0 | 0 | 0 | 3.12525 | 6.93633 | 7.793641 | #DIV/0! | 14 | 3 | 0.053492 |
| PPS_4081 | Amino acid transport and metabolism | 0 | 0 | 0.71471 | 120.843 | 46.82022 | 73.4829 | 337.4039569 | 210 | 13 | 0.065805 |
| PPS_4077 | Energy production and conversion | 0 | 0.755256 | 0 | 34.37775 | 55.49064 | 30.06119 | 158.79324 | 93 | 17 | 0.03684 |
| PPS_3300 | Posttranslational modification | 0 | 0 | 0.71471 | 5.20875 | 6.93633 | 11.13377 | 32.57102676 | 20 | 6 | 0.04829 |
| PPS_2020 | Amino acid transport and metabolism | 0 | 0 | 0.71471 | 7.29225 | 3.468165 | 4.453509 | 21.28683646 | 14 | 5 | 0.046781 |
| PPS_4429 | Energy production and conversion | 0 | 0.755256 | 0 | 6.2505 | 0 | 4.453509 | 14.17268685 | 11 | 5 | 0.214487 |
| PPS_4061 | Coenzyme metabolism | 8.313414 | 18.8814 | 12.86479 | 156.2625 | 171.6742 | 144.739 | 11.7993098 | 429 | 22 | 0.000945 |
| PPS_3996 | Function unknown | 0 | 0 | 0.71471 | 1.04175 | 6.93633 | 0 | 11.16267419 | 6 | 5 | 0.378725 |
| PPS_3957 | Energy production and conversion | 1.187631 | 2.265768 | 0 | 10.4175 | 13.87266 | 13.36053 | 10.90250074 | 34 | 11 | 0.001932 |
| PPS_3131 | Lipid metabolism | 1.187631 | 0 | 0 | 5.20875 | 0 | 5.566886 | 9.073222005 | 11 | 5 | 0.213699 |
| PPS_0935 | Amino acid transport and metabolism | 0 | 0.755256 | 0 | 1.04175 | 3.468165 | 2.226755 | 8.919714491 | 6 | 4 | 0.091033 |
| PPS_3930 | Function unknown | 0 | 0.755256 | 0 | 3.12525 | 3.468165 | 0 | 8.730037731 | 6 | 4 | 0.215753 |
| PPS_4027 | Energy production and conversion | 2.375261 | 0 | 0.71471 | 6.2505 | 12.13858 | 7.793641 | 8.473449075 | 23 | 5 | 0.034706 |
| PPS_0595 | Coenzyme metabolism | 0 | 0.755256 | 0 | 2.0835 | 3.468165 | 0 | 7.350704397 | 5 | 3 | 0.250157 |
| PPS_4124 | Amino acid transport and metabolism | 0 | 0.755256 | 0 | 3.12525 | 0 | 2.226755 | 7.086343427 | 6 | 2 | 0.236538 |
| PPS_4192 | Transcription | 0 | 0 | 0.71471 | 1.04175 | 1.734082 | 2.226755 | 6.999459896 | 5 | 3 | 0.032176 |
| PPS_2834 | Posttranslational modification | 0 | 0 | 0.71471 | 2.0835 | 1.734082 | 1.113377 | 6.899241305 | 5 | 4 | 0.020265 |
| PPS_0477 | Inorganic ion transport and metabolism | 0 | 0.755256 | 1.429421 | 6.2505 | 3.468165 | 4.453509 | 6.487079706 | 15 | 4 | 0.022642 |
| PPS_5082 | Amino acid transport and metabolism | 0 | 0 | 0.71471 | 3.12525 | 0 | 1.113377 | 5.930551924 | 5 | 3 | 0.326862 |
| PPS_4266 | Transcription | 0 | 1.510512 | 0 | 2.0835 | 3.468165 | 3.340132 | 5.886609769 | 9 | 2 | 0.021965 |
| PPS_4988 | Transcription | 2.375261 | 0 | 0 | 4.167 | 8.670412 | 1.113377 | 5.873370439 | 12 | 2 | 0.214086 |
| PPS_4932 | Translation | 0 | 0.755256 | 0 | 2.0835 | 0 | 2.226755 | 5.707010094 | 5 | 2 | 0.236132 |
| PPS_4722 | Translation | 1.187631 | 0 | 0 | 1.04175 | 3.468165 | 2.226755 | 5.672360726 | 6 | 3 | 0.100665 |
| PPS_3721 | Posttranslational modification | 0 | 1.510512 | 0 | 1.04175 | 5.202247 | 2.226755 | 5.607866678 | 8 | 3 | 0.193037 |
| PPS_0381 | DNA replication | 1.187631 | 0 | 0.71471 | 3.12525 | 5.202247 | 1.113377 | 4.962766511 | 9 | 4 | 0.158924 |
| PPS_0752 | Energy production and conversion | 0 | 3.021025 | 3.573552 | 14.5845 | 12.13858 | 5.566886 | 4.896442076 | 35 | 7 | 0.070064 |
| PPS_0593 | Transcription | 0 | 1.510512 | 0 | 6.2505 | 0 | 1.113377 | 4.875085857 | 9 | 2 | 0.419382 |
| PPS_3301 | Posttranslational modification | 19.00209 | 14.34987 | 15.00892 | 84.38175 | 67.62921 | 80.16316 | 4.80086686 | 248 | 12 | 0.004019 |
| PPS_2160 | Defense mechanisms | 1.187631 | 0 | 0 | 1.04175 | 3.468165 | 1.113377 | 4.734882992 | 5 | 2 | 0.197415 |
| PPS_0530 | Inorganic ion transport and metabolism | 0 | 0 | 1.429421 | 2.0835 | 3.468165 | 1.113377 | 4.66275701 | 7 | 4 | 0.112497 |
| PPS_5118 | Energy production and conversion | 1.187631 | 0 | 0.71471 | 0 | 5.202247 | 3.340132 | 4.490456114 | 8 | 2 | 0.280918 |
| PPS_3132 | Function unknown | 0 | 1.510512 | 0.71471 | 4.167 | 3.468165 | 2.226755 | 4.431879617 | 11 | 2 | 0.026303 |
| PPS_3384 |  | 0 | 0 | 2.144131 | 3.12525 | 3.468165 | 2.226755 | 4.113633092 | 10 | 2 | 0.069846 |
| PPS_4467 | Amino acid transport and metabolism | 2.375261 | 0 | 0.71471 | 5.20875 | 5.202247 | 2.226755 | 4.089924776 | 13 | 5 | 0.065689 |
| PPS_0599 | Energy production and conversion | 0 | 1.510512 | 0 | 1.04175 | 1.734082 | 3.340132 | 4.04893367 | 7 | 4 | 0.150145 |
| PPS_3442 | Amino acid transport and metabolism | 1.187631 | 1.510512 | 0 | 0 | 8.670412 | 2.226755 | 4.038765445 | 10 | 2 | 0.403826 |
| PPS_0364 | Lipid metabolism | 0 | 0.755256 | 3.573552 | 8.334 | 5.202247 | 3.340132 | 3.898620113 | 20 | 8 | 0.088249 |
| PPS_4062 | Cell motility and secretion | 2.375261 | 3.776281 | 0.71471 | 14.5845 | 5.202247 | 6.680264 | 3.854651551 | 31 | 10 | 0.144944 |
| PPS_0864 | Energy production and conversion | 4.750523 | 1.510512 | 3.573552 | 9.37575 | 17.34082 | 11.13377 | 3.84869714 | 40 | 6 | 0.046304 |
| PPS_1651 | Cell envelope biogenesis | 0 | 0 | 1.429421 | 1.04175 | 0 | 4.453509 | 3.844395514 | 7 | 3 | 0.42488 |
| PPS_0405 | Signal transduction mechanisms | 1.187631 | 0 | 1.429421 | 2.0835 | 3.468165 | 4.453509 | 3.823071108 | 11 | 2 | 0.04836 |
| PPS_3187 | General function prediction only | 1.187631 | 0 | 0 | 2.0835 | 0 | 2.226755 | 3.629288802 | 5 | 3 | 0.291619 |
| PPS_1916 | Carbohydrate transport and metabolism | 0 | 0.755256 | 0.71471 | 4.167 | 0 | 1.113377 | 3.592175037 | 7 | 4 | 0.41592 |
| PPS_0514 | Coenzyme metabolism | 4.750523 | 7.552562 | 5.002973 | 11.45925 | 26.01124 | 24.4943 | 3.580525915 | 69 | 9 | 0.079264 |
|  |  | 0 | 2.265768 | 0 | 2.0835 | 3.468165 | 2.226755 | 3.433015941 | 9 | 2 | 0.120136 |
| PPS_0590 | Nucleotide transport and metabolism | 0 | 1.510512 | 0.71471 | 5.20875 | 0 | 2.226755 | 3.341465276 | 10 | 3 | 0.369769 |
| PPS_1399 | General function prediction only | 1.187631 | 0.755256 | 0.71471 | 3.12525 | 3.468165 | 2.226755 | 3.318851062 | 10 | 5 | 0.019233 |
| PPS_1571 | Posttranslational modification | 2.375261 | 21.14717 | 20.01189 | 32.29425 | 46.82022 | 63.4625 | 3.275047381 | 173 | 6 | 0.045601 |
| PPS_0050 | Energy production and conversion | 1.187631 | 1.510512 | 0 | 3.12525 | 3.468165 | 2.226755 | 3.26897777 | 10 | 3 | 0.027709 |
| PPS_1700 | Lipid metabolism | 1.187631 | 2.265768 | 0.71471 | 5.20875 | 3.468165 | 4.453509 | 3.150210847 | 16 | 5 | 0.012078 |
| PPS_0902 | Intracellular trafficking and secretion | 2.375261 | 1.510512 | 1.429421 | 5.20875 | 6.93633 | 4.453509 | 3.12285635 | 19 | 2 | 0.023327 |
| PPS_4173 | Inorganic ion transport and metabolism | 0 | 0.755256 | 1.429421 | 0 | 3.468165 | 3.340132 | 3.116385893 | 8 | 3 | 0.306948 |
| PPS_1435 | General function prediction only | 0 | 1.510512 | 0 | 0 | 3.468165 | 1.113377 | 3.033104722 | 5 | 3 | 0.436955 |
| PPS_4856 | General function prediction only | 0 | 1.510512 | 0.71471 | 1.04175 | 3.468165 | 2.226755 | 3.027413478 | 8 | 4 | 0.156293 |
| PPS_4736 | General function prediction only | 1.187631 | 0 | 0.71471 | 0 | 3.468165 | 2.226755 | 2.993637409 | 6 | 4 | 0.339694 |
| PPS_1806 | Amino acid transport and metabolism | 1.187631 | 0 | 0.71471 | 0 | 3.468165 | 2.226755 | 2.993637409 | 6 | 2 | 0.339694 |
| PPS_3190 | Cell envelope biogenesis | 0 | 3.776281 | 2.858842 | 10.4175 | 6.93633 | 2.226755 | 2.951050885 | 25 | 5 | 0.20361 |
| PPS_1406 | Coenzyme metabolism | 0 | 0.755256 | 0.71471 | 2.0835 | 0 | 2.226755 | 2.9322126 | 6 | 3 | 0.318517 |
| PPS_4157 | Cell division and chromosome partitioning | 1.187631 | 2.265768 | 1.429421 | 4.167 | 3.468165 | 6.680264 | 2.931795261 | 18 | 3 | 0.071886 |
| PPS_0445 | Translation | 4.750523 | 0 | 0.71471 | 0 | 6.93633 | 8.907018 | 2.898933666 | 17 | 2 | 0.340679 |
| PPS_5119 | Energy production and conversion | 1.187631 | 0.755256 | 0 | 2.0835 | 3.468165 | 0 | 2.857430921 | 6 | 2 | 0.356816 |
| PPS_3039 | Secondary metabolites biosynthesis | 1.187631 | 0.755256 | 0 | 1.04175 | 0 | 4.453509 | 2.828399014 | 7 | 3 | 0.474507 |
| PPS_4436 | Function unknown | 1.187631 | 0 | 0.71471 | 3.12525 | 0 | 2.226755 | 2.813378042 | 7 | 4 | 0.343229 |
| PPS_0465 | Translation | 13.06394 | 9.063074 | 13.5795 | 59.37975 | 22.54307 | 17.81404 | 2.793240241 | 128 | 8 | 0.244835 |
| PPS_4566 | Translation | 1.187631 | 0 | 1.429421 | 1.04175 | 1.734082 | 4.453509 | 2.762399423 | 9 | 2 | 0.276524 |
| PPS_4284 |  | 2.375261 | 0.755256 | 1.429421 | 6.2505 | 5.202247 | 1.113377 | 2.755766357 | 15 | 6 | 0.225406 |
|  | General function prediction only | 0 | 0 | 2.144131 | 4.167 | 1.734082 | 0 | 2.752202016 | 8 | 3 | 0.433574 |
| PPS_0098 | Signal transduction mechanisms | 0 | 0.755256 | 0.71471 | 0 | 1.734082 | 2.226755 | 2.694508147 | 5 | 4 | 0.345613 |
| PPS_3979 | Cell envelope biogenesis | 3.562892 | 6.042049 | 3.573552 | 9.37575 | 13.87266 | 12.24715 | 2.693445932 | 44 | 13 | 0.013243 |
| PPS_0987 | Secondary metabolites biosynthesis | 1.187631 | 0.755256 | 0.71471 | 3.12525 | 1.734082 | 2.226755 | 2.666350981 | 9 | 3 | 0.054457 |
| PPS_2905 | Cell envelope biogenesis | 0 | 0.755256 | 0.71471 | 2.0835 | 1.734082 | 0 | 2.597053862 | 5 | 2 | 0.351058 |
| PPS_1468 | Translation | 2.375261 | 0.755256 | 1.429421 | 2.0835 | 5.202247 | 4.453509 | 2.57443316 | 14 | 5 | 0.108986 |
| PPS_5074 | Secondary metabolites biosynthesis | 0 | 0.755256 | 1.429421 | 1.04175 | 3.468165 | 1.113377 | 2.573969537 | 7 | 2 | 0.291505 |
| PPS_3892 | DNA replication | 1.187631 | 3.021025 | 1.429421 | 5.20875 | 6.93633 | 2.226755 | 2.54906706 | 18 | 12 | 0.156818 |
| PPS_2983 |  | 0 | 0.755256 | 1.429421 | 2.0835 | 3.468165 | 0 | 2.541183331 | 7 | 2 | 0.387461 |
| PPS_1507 | Coenzyme metabolism | 1.187631 | 0.755256 | 0.71471 | 2.0835 | 3.468165 | 1.113377 | 2.507920324 | 8 | 3 | 0.185045 |
| PPS_1585 | Function unknown | 1.187631 | 0.755256 | 0 | 3.12525 | 1.734082 | 0 | 2.501088802 | 6 | 3 | 0.400166 |
| PPS_3008 | Posttranslational modification | 4.750523 | 3.021025 | 7.147104 | 14.5845 | 8.670412 | 13.36053 | 2.454339758 | 49 | 6 | 0.035547 |
|  |  | 9.501045 | 0.755256 | 0.71471 | 5.20875 | 15.60674 | 5.566886 | 2.404735211 | 29 | 9 | 0.317676 |
| PPS_1695 | Cell envelope biogenesis | 2.375261 | 1.510512 | 0.71471 | 3.12525 | 3.468165 | 4.453509 | 2.401252535 | 14 | 6 | 0.027521 |
| PPS_3862 | Transcription | 4.750523 | 1.510512 | 1.429421 | 4.167 | 8.670412 | 5.566886 | 2.393134958 | 22 | 4 | 0.109437 |
| PPS_4979 | Coenzyme metabolism | 0 | 0 | 2.144131 | 0 | 1.734082 | 3.340132 | 2.366559495 | 7 | 3 | 0.465111 |
| PPS_1350 | DNA replication | 4.750523 | 5.286793 | 4.288263 | 7.29225 | 17.34082 | 8.907018 | 2.34127319 | 42 | 10 | 0.175093 |
| PPS_1302 | Energy production and conversion | 0 | 0 | 1.429421 | 0 | 0 | 3.340132 | 2.336702886 | 5 | 3 | 0.638931 |
| PPS_2476 |  | 1.187631 | 0.755256 | 0 | 1.04175 | 3.468165 | 0 | 2.321244251 | 5 | 3 | 0.499247 |
| PPS_3866 | Cell envelope biogenesis | 29.69077 | 19.63666 | 24.30015 | 60.4215 | 58.9588 | 51.21535 | 2.317007502 | 223 | 22 | 0.001368 |
| PPS_1437 |  | 0 | 0 | 2.858842 | 3.12525 | 3.468165 | 0 | 2.306323857 | 9 | 4 | 0.442249 |
| PPS_3823 | Energy production and conversion | 1.187631 | 0.755256 | 0.71471 | 1.04175 | 1.734082 | 3.340132 | 2.301313449 | 8 | 2 | 0.228904 |
| PPS_3519 | Energy production and conversion | 2.375261 | 0.755256 | 1.429421 | 4.167 | 5.202247 | 1.113377 | 2.298852277 | 13 | 4 | 0.244395 |
| PPS_0507 | Posttranslational modification | 0 | 1.510512 | 4.288263 | 3.12525 | 3.468165 | 6.680264 | 2.289048763 | 19 | 4 | 0.215322 |
| PPS_3385 | Amino acid transport and metabolism | 0 | 0 | 1.429421 | 1.04175 | 0 | 2.226755 | 2.28659359 | 5 | 3 | 0.489865 |
| PPS_0801 | Coenzyme metabolism | 0 | 0 | 1.429421 | 1.04175 | 0 | 2.226755 | 2.28659359 | 5 | 2 | 0.489865 |
| PPS_4541 | Translation | 0 | 0 | 1.429421 | 1.04175 | 0 | 2.226755 | 2.28659359 | 5 | 4 | 0.489865 |
| PPS_0080 |  | 0 | 0 | 1.429421 | 1.04175 | 0 | 2.226755 | 2.28659359 | 5 | 2 | 0.489865 |
| PPS_4212 | Coenzyme metabolism | 0 | 2.265768 | 1.429421 | 2.0835 | 5.202247 | 1.113377 | 2.272988934 | 11 | 3 | 0.342698 |
| PPS_3130 | Inorganic ion transport and metabolism | 0 | 0.755256 | 0.71471 | 0 | 0 | 3.340132 | 2.272250164 | 5 | 2 | 0.635069 |
| PPS_5269 | Cell envelope biogenesis | 2.375261 | 2.265768 | 0.71471 | 5.20875 | 6.93633 | 0 | 2.267675281 | 15 | 7 | 0.392231 |
| PPS_4752 | Amino acid transport and metabolism | 3.562892 | 1.510512 | 2.858842 | 10.4175 | 1.734082 | 5.566886 | 2.233726611 | 25 | 7 | 0.322712 |
| PPS_4845 | Posttranslational modification | 11.87631 | 11.32884 | 7.861815 | 26.04375 | 24.27715 | 18.92741 | 2.229001796 | 92 | 13 | 0.011911 |
| PPS_4309 | Energy production and conversion | 1.187631 | 0 | 0.71471 | 3.12525 | 0 | 1.113377 | 2.228111131 | 6 | 2 | 0.492879 |
| PPS_3659 | DNA replication | 0 | 1.510512 | 1.429421 | 2.0835 | 0 | 4.453509 | 2.223523021 | 10 | 5 | 0.457529 |
| PPS_0075 | General function prediction only | 0 | 0.755256 | 0.71471 | 1.04175 | 0 | 2.226755 | 2.223523021 | 5 | 2 | 0.457529 |
| PPS_2184 | Signal transduction mechanisms | 1.187631 | 0.755256 | 2.858842 | 2.0835 | 5.202247 | 3.340132 | 2.212927917 | 14 | 6 | 0.163059 |
| PPS_3455 | Carbohydrate transport and metabolism | 0 | 0.755256 | 0.71471 | 2.0835 | 0 | 1.113377 | 2.174795879 | 5 | 2 | 0.44887 |
| PPS_3463 | Carbohydrate transport and metabolism | 2.375261 | 0 | 0.71471 | 2.0835 | 3.468165 | 1.113377 | 2.156991311 | 8 | 2 | 0.291203 |
| PPS_2977 | Lipid metabolism | 2.375261 | 0 | 1.429421 | 3.12525 | 1.734082 | 3.340132 | 2.155098373 | 11 | 6 | 0.168238 |
| PPS_1424 | Cell envelope biogenesis | 3.562892 | 1.510512 | 2.858842 | 5.20875 | 5.202247 | 6.680264 | 2.154655949 | 23 | 7 | 0.018475 |
| PPS_4573 | Posttranslational modification | 104.5115 | 64.19677 | 71.47104 | 165.6383 | 176.8764 | 173.6869 | 2.149233861 | 690 | 36 | 0.012994 |
| PPS_5274 | Energy production and conversion | 8.313414 | 11.32884 | 10.72066 | 26.04375 | 19.07491 | 20.04079 | 2.146020932 | 91 | 16 | 0.020846 |
| PPS_1458 | General function prediction only | 3.562892 | 1.510512 | 4.288263 | 3.12525 | 6.93633 | 10.0204 | 2.145128167 | 27 | 6 | 0.207613 |
| PPS_0824 | Signal transduction mechanisms | 1.187631 | 0.755256 | 1.429421 | 1.04175 | 1.734082 | 4.453509 | 2.143737245 | 10 | 7 | 0.341784 |
| PPS_4249 | Signal transduction mechanisms | 0 | 0.755256 | 0.71471 | 3.12525 | 0 | 0 | 2.126068737 | 5 | 3 | 0.652945 |
| PPS_3649 | Energy production and conversion | 0 | 1.510512 | 0 | 2.0835 | 0 | 1.113377 | 2.11641919 | 5 | 2 | 0.514557 |
| PPS_3655 | Signal transduction mechanisms | 4.750523 | 1.510512 | 0.71471 | 7.29225 | 5.202247 | 2.226755 | 2.110348239 | 19 | 2 | 0.251712 |
| PPS_3236 | Amino acid transport and metabolism | 0 | 0.755256 | 2.144131 | 1.04175 | 1.734082 | 3.340132 | 2.109398747 | 9 | 2 | 0.311776 |
| PPS_1912 | alpha-L-glutamate ligase-like protein | 0 | 0.755256 | 1.429421 | 0 | 3.468165 | 1.113377 | 2.097125593 | 6 | 4 | 0.52763 |
| PPS_5247 | Inorganic ion transport and metabolism | 1.187631 | 1.510512 | 3.573552 | 6.2505 | 3.468165 | 3.340132 | 2.082179773 | 19 | 5 | 0.138405 |
| PPS_0409 | Amino acid transport and metabolism | 1.187631 | 0 | 0.71471 | 0 | 1.734082 | 2.226755 | 2.082085616 | 5 | 2 | 0.432729 |
| PPS_0697 | Function unknown | 1.187631 | 0.755256 | 0.71471 | 1.04175 | 0 | 4.453509 | 2.067754683 | 8 | 3 | 0.555399 |
| PPS_2934 | Cell envelope biogenesis | 13.06394 | 5.286793 | 6.432394 | 17.70975 | 20.80899 | 12.24715 | 2.048405562 | 67 | 10 | 0.067859 |
| PPS_3168 | Energy production and conversion | 3.562892 | 3.021025 | 0.71471 | 6.2505 | 8.670412 | 0 | 2.044345062 | 19 | 6 | 0.433814 |
| PPS_4187 | Amino acid transport and metabolism | 0 | 1.510512 | 2.858842 | 3.12525 | 3.468165 | 2.226755 | 2.018643787 | 13 | 4 | 0.207004 |
| PPS_0441 | Translation | 46.31759 | 39.27332 | 37.16494 | 83.34 | 58.9588 | 104.6575 | 2.011767676 | 351 | 12 | 0.08263 |
| PPS_3681 | Transcription | 0 | 0 | 2.144131 | 2.0835 | 0 | 2.226755 | 2.010256838 | 7 | 3 | 0.515826 |
| PPS_3172 | Inorganic ion transport and metabolism | 1.187631 | 0 | 0.71471 | 2.0835 | 1.734082 | 0 | 2.006781273 | 5 | 3 | 0.445511 |
| PPS_1706 | Transcription | 1.187631 | 0.755256 | 0 | 1.04175 | 1.734082 | 1.113377 | 2.001768546 | 5 | 3 | 0.202136 |
| PPS_4269 | Cell envelope biogenesis | 17.81446 | 3.776281 | 4.288263 | 10.4175 | 34.68165 | 6.680264 | 2.000827157 | 62 | 13 | 0.447296 |
| PPS_2729 | Inorganic ion transport and metabolism | 2.375261 | 2.265768 | 1.429421 | 5.20875 | 6.93633 | 0 | 2.000688319 | 16 | 4 | 0.434317 |
| PPS_4037 | Defense mechanisms | 3.562892 | 3.776281 | 4.288263 | 9.37575 | 10.40449 | 3.340132 | 1.988433023 | 32 | 4 | 0.223353 |
| PPS_4775 | Energy production and conversion | 0 | 0.755256 | 1.429421 | 2.0835 | 0 | 2.226755 | 1.972948188 | 7 | 4 | 0.452453 |
| PPS_1730 | Cell envelope biogenesis | 2.375261 | 0.755256 | 3.573552 | 5.20875 | 3.468165 | 4.453509 | 1.958575133 | 19 | 3 | 0.102897 |
| PPS_5037 | Amino acid transport and metabolism | 2.375261 | 2.265768 | 1.429421 | 7.29225 | 3.468165 | 1.113377 | 1.95599847 | 17 | 3 | 0.395277 |
| PPS_3250 | Function unknown | 3.562892 | 0 | 0.71471 | 3.12525 | 5.202247 | 0 | 1.946767509 | 10 | 4 | 0.512536 |
| PPS_2773 |  | 0 | 2.265768 | 0 | 1.04175 | 0 | 3.340132 | 1.933949491 | 7 | 2 | 0.602551 |
| PPS_4525 | Coenzyme metabolism | 5.938153 | 0 | 1.429421 | 5.20875 | 3.468165 | 5.566886 | 1.933309549 | 19 | 4 | 0.329453 |
| PPS_0392 | Function unknown | 2.375261 | 0.755256 | 0 | 2.0835 | 1.734082 | 2.226755 | 1.930778887 | 8 | 3 | 0.298485 |
| PPS_3578 | Posttranslational modification | 59.38153 | 43.80486 | 45.74147 | 85.4235 | 91.90637 | 109.111 | 1.923353008 | 405 | 37 | 0.008219 |
| PPS_1421 | Cell envelope biogenesis | 0 | 2.265768 | 0 | 2.0835 | 0 | 2.226755 | 1.902336698 | 7 | 4 | 0.549292 |
| PPS_4783 |  | 0 | 2.265768 | 0 | 2.0835 | 0 | 2.226755 | 1.902336698 | 7 | 2 | 0.549292 |
| PPS_4751 | Lipid metabolism | 2.375261 | 1.510512 | 0 | 1.04175 | 5.202247 | 1.113377 | 1.893413063 | 9 | 3 | 0.507769 |
| PPS_4766 | Cell envelope biogenesis | 1.187631 | 5.286793 | 2.858842 | 11.45925 | 1.734082 | 4.453509 | 1.890746767 | 28 | 9 | 0.448968 |
| PPS_1299 | Posttranslational modification | 2.375261 | 1.510512 | 0 | 7.29225 | 0 | 0 | 1.876653347 | 11 | 2 | 0.691773 |
| PPS_4377 | Translation | 10.68868 | 17.37089 | 12.86479 | 20.835 | 12.13858 | 43.42171 | 1.866743907 | 116 | 4 | 0.331605 |
| PPS_1423 | Cell envelope biogenesis | 4.750523 | 6.042049 | 3.573552 | 6.2505 | 10.40449 | 10.0204 | 1.856825819 | 38 | 8 | 0.070391 |
| PPS_0909 | Energy production and conversion | 7.125784 | 4.531537 | 3.573552 | 9.37575 | 12.13858 | 6.680264 | 1.851147381 | 39 | 5 | 0.094699 |
| PPS_3177 | Signal transduction mechanisms | 0 | 1.510512 | 1.429421 | 2.0835 | 0 | 3.340132 | 1.844814661 | 9 | 4 | 0.503763 |
| PPS_1886 | Posttranslational modification | 24.94024 | 13.59461 | 5.717683 | 29.169 | 26.01124 | 25.60768 | 1.825610863 | 113 | 14 | 0.156097 |
| PPS_3323 |  | 2.375261 | 0 | 0 | 2.0835 | 0 | 2.226755 | 1.814644401 | 6 | 3 | 0.579387 |
| PPS_1374 | Cell division and chromosome partitioning | 0 | 0 | 2.144131 | 1.04175 | 1.734082 | 1.113377 | 1.813885991 | 6 | 2 | 0.50663 |
| PPS_4721 | Translation | 14.25157 | 15.86038 | 12.86479 | 43.7535 | 17.34082 | 16.70066 | 1.810165054 | 118 | 7 | 0.322315 |
| PPS_0184 |  | 8.313414 | 0.755256 | 2.858842 | 7.29225 | 8.670412 | 5.566886 | 1.80503259 | 29 | 5 | 0.290282 |
| PPS_3129 | Inorganic ion transport and metabolism | 1.187631 | 0 | 2.144131 | 3.12525 | 1.734082 | 1.113377 | 1.792658015 | 9 | 2 | 0.363619 |
| PPS_1623 | General function prediction only | 0 | 3.021025 | 0.71471 | 0 | 0 | 6.680264 | 1.788205935 | 11 | 3 | 0.713989 |
| PPS_4562 | Carbohydrate transport and metabolism | 2.375261 | 3.021025 | 3.573552 | 5.20875 | 5.202247 | 5.566886 | 1.781290087 | 24 | 7 | 0.013583 |
| PPS_4796 | DNA replication | 0 | 0.755256 | 1.429421 | 1.04175 | 1.734082 | 1.113377 | 1.780221815 | 6 | 4 | 0.310076 |
| PPS_1686 | Coenzyme metabolism | 5.938153 | 0 | 2.144131 | 5.20875 | 6.93633 | 2.226755 | 1.778189605 | 19 | 3 | 0.400082 |
| PPS_0865 | Energy production and conversion | 8.313414 | 1.510512 | 3.573552 | 9.37575 | 8.670412 | 5.566886 | 1.762499394 | 33 | 3 | 0.234102 |
| PPS_1508 | Posttranslational modification | 3.562892 | 1.510512 | 2.144131 | 4.167 | 5.202247 | 3.340132 | 1.760902879 | 18 | 4 | 0.087982 |
|  | Secondary metabolites biosynthesis | 9.501045 | 14.34987 | 11.43537 | 19.79325 | 24.27715 | 17.81404 | 1.753781976 | 92 | 13 | 0.023506 |
| PPS_0591 | Secondary metabolites biosynthesis | 13.06394 | 6.042049 | 4.288263 | 26.04375 | 10.40449 | 4.453509 | 1.748367896 | 60 | 6 | 0.470893 |
| PPS_1450 | Carbohydrate transport and metabolism | 2.375261 | 3.021025 | 2.858842 | 5.20875 | 6.93633 | 2.226755 | 1.740958448 | 21 | 3 | 0.275351 |
| PPS_0606 | Lipid metabolism | 10.68868 | 10.57359 | 9.291235 | 11.45925 | 22.54307 | 18.92741 | 1.732362552 | 77 | 13 | 0.147226 |
| PPS_3041 | Energy production and conversion | 10.68868 | 9.063074 | 7.861815 | 12.501 | 20.80899 | 14.4739 | 1.730450037 | 69 | 2 | 0.103823 |
| PPS_0470 | Intracellular trafficking and secretion | 9.501045 | 3.021025 | 4.288263 | 13.54275 | 12.13858 | 3.340132 | 1.726406011 | 41 | 4 | 0.350653 |
| PPS_4268 | Defense mechanisms | 5.938153 | 4.531537 | 2.858842 | 9.37575 | 6.93633 | 6.680264 | 1.725046955 | 34 | 6 | 0.059858 |
| PPS_0006 | Intracellular trafficking and secretion | 5.938153 | 0.755256 | 0.71471 | 4.167 | 5.202247 | 3.340132 | 1.715601187 | 17 | 3 | 0.418733 |
| PPS_4205 | Posttranslational modification | 1.187631 | 4.531537 | 4.288263 | 4.167 | 5.202247 | 7.793641 | 1.715014529 | 27 | 6 | 0.192571 |
| PPS_0460 | Translation | 16.62683 | 9.81833 | 12.15008 | 27.0855 | 15.60674 | 23.38092 | 1.711951291 | 100 | 10 | 0.09541 |
| PPS_3994 | Cell envelope biogenesis | 24.94024 | 7.552562 | 14.29421 | 20.835 | 31.21348 | 27.83443 | 1.707373677 | 114 | 5 | 0.150809 |
| PPS_2034 | Translation | 16.62683 | 7.552562 | 7.861815 | 13.54275 | 27.74532 | 13.36053 | 1.705572418 | 76 | 2 | 0.263359 |
| PPS_1356 | Posttranslational modification | 8.313414 | 8.307818 | 8.576525 | 13.54275 | 10.40449 | 18.92741 | 1.701526749 | 66 | 11 | 0.141315 |
| PPS_5271 | Cell envelope biogenesis | 8.313414 | 3.776281 | 4.288263 | 12.501 | 8.670412 | 6.680264 | 1.700558521 | 41 | 8 | 0.163707 |
| PPS_1881 | Posttranslational modification | 52.25575 | 59.66524 | 49.31502 | 61.46325 | 110.9813 | 100.204 | 1.69099005 | 405 | 25 | 0.126928 |
| PPS_3645 |  | 1.187631 | 0.755256 | 0 | 1.04175 | 0 | 2.226755 | 1.682292842 | 5 | 2 | 0.587191 |
| PPS_0249 | Energy production and conversion | 1.187631 | 0.755256 | 0 | 1.04175 | 0 | 2.226755 | 1.682292842 | 5 | 3 | 0.587191 |
| PPS_3780 | Cell motility and secretion | 20.18972 | 15.86038 | 17.15305 | 22.9185 | 20.80899 | 45.64847 | 1.679899714 | 137 | 19 | 0.267153 |
| PPS_3431 | General function prediction only | 1.187631 | 0.755256 | 1.429421 | 1.04175 | 3.468165 | 1.113377 | 1.667490832 | 8 | 2 | 0.448155 |
| PPS_3977 | Nucleotide transport and metabolism | 5.938153 | 6.797305 | 8.576525 | 10.4175 | 13.87266 | 11.13377 | 1.662160239 | 54 | 8 | 0.026309 |
| PPS_1728 |  | 3.562892 | 0 | 0 | 4.167 | 1.734082 | 0 | 1.656261987 | 8 | 4 | 0.669428 |
| PPS_0194 |  | 0 | 3.021025 | 2.144131 | 5.20875 | 0 | 3.340132 | 1.655106245 | 15 | 6 | 0.565791 |
| PPS_3861 | General function prediction only | 3.562892 | 4.531537 | 3.573552 | 6.2505 | 5.202247 | 7.793641 | 1.649504583 | 30 | 2 | 0.061701 |
| PPS_1447 | Cell envelope biogenesis | 1.187631 | 0.755256 | 0.71471 | 1.04175 | 0 | 3.340132 | 1.648813368 | 7 | 5 | 0.620614 |
| PPS_0657 | Amino acid transport and metabolism | 1.187631 | 1.510512 | 0.71471 | 1.04175 | 3.468165 | 1.113377 | 1.647680532 | 8 | 3 | 0.456546 |
| PPS_0493 | Amino acid transport and metabolism | 1.187631 | 0 | 0.71471 | 3.12525 | 0 | 0 | 1.64284422 | 5 | 3 | 0.740217 |
| PPS_0971 | Function unknown | 3.562892 | 0.755256 | 0 | 3.12525 | 1.734082 | 2.226755 | 1.641001387 | 10 | 5 | 0.492921 |
| PPS_1587 | Nucleotide transport and metabolism | 5.938153 | 6.042049 | 4.288263 | 11.45925 | 5.202247 | 10.0204 | 1.640098969 | 42 | 10 | 0.201533 |
| PPS_3522 |  | 4.750523 | 2.265768 | 0 | 4.167 | 1.734082 | 5.566886 | 1.634477354 | 17 | 8 | 0.450964 |
| PPS_1882 | Posttranslational modification | 4.750523 | 2.265768 | 5.002973 | 10.4175 | 3.468165 | 5.566886 | 1.618447786 | 31 | 4 | 0.356646 |
| PPS_2037 | Translation | 23.75261 | 17.37089 | 17.15305 | 32.29425 | 41.61798 | 20.04079 | 1.612192404 | 140 | 21 | 0.189153 |
| PPS_1590 | Function unknown | 1.187631 | 0.755256 | 0 | 3.12525 | 0 | 0 | 1.608560012 | 5 | 3 | 0.748404 |
| PPS_5025 | Defense mechanisms | 2.375261 | 0 | 0 | 2.0835 | 1.734082 | 0 | 1.607226314 | 5 | 2 | 0.66313 |
| PPS_3825 | Amino acid transport and metabolism | 2.375261 | 0 | 0 | 2.0835 | 1.734082 | 0 | 1.607226314 | 5 | 3 | 0.66313 |
|  | Amino acid transport and metabolism | 1.187631 | 1.510512 | 2.144131 | 2.0835 | 3.468165 | 2.226755 | 1.606356638 | 12 | 3 | 0.146411 |
| PPS_1309 | General function prediction only | 3.562892 | 0 | 3.573552 | 5.20875 | 1.734082 | 4.453509 | 1.596921588 | 18 | 3 | 0.422957 |
| PPS_0455 | Translation | 11.87631 | 3.776281 | 7.861815 | 10.4175 | 10.40449 | 16.70066 | 1.595730729 | 57 | 5 | 0.212087 |
| PPS_4030 | Cell envelope biogenesis | 36.81655 | 31.72076 | 45.02676 | 51.04575 | 39.8839 | 90.18356 | 1.59481086 | 289 | 11 | 0.275246 |
| PPS_5040 | Function unknown | 0 | 4.531537 | 2.144131 | 2.0835 | 5.202247 | 3.340132 | 1.591732635 | 17 | 7 | 0.459946 |
| PPS_2535 | Energy production and conversion | 13.06394 | 8.307818 | 4.288263 | 14.5845 | 17.34082 | 8.907018 | 1.591282724 | 60 | 2 | 0.227332 |
| PPS_3425 | General function prediction only | 0 | 0.755256 | 1.429421 | 0 | 3.468165 | 0 | 1.587495443 | 5 | 3 | 0.754659 |
| PPS_1904 |  | 39.19181 | 12.0841 | 16.43834 | 57.29625 | 8.670412 | 41.19496 | 1.582556441 | 169 | 4 | 0.482011 |
| PPS_1440 | Cell envelope biogenesis | 0 | 0.755256 | 2.144131 | 0 | 3.468165 | 1.113377 | 1.580175874 | 7 | 3 | 0.669222 |
| PPS_2029 |  | 2.375261 | 0 | 1.429421 | 3.12525 | 1.734082 | 1.113377 | 1.569831462 | 9 | 2 | 0.473055 |
| PPS_1961 | Amino acid transport and metabolism | 5.938153 | 9.81833 | 10.72066 | 11.45925 | 12.13858 | 17.81404 | 1.564061064 | 67 | 17 | 0.123066 |
| PPS_4367 | General function prediction only | 14.25157 | 14.34987 | 10.00595 | 29.169 | 15.60674 | 15.58728 | 1.563509943 | 96 | 15 | 0.245666 |
| PPS_2032 | Translation | 29.69077 | 14.34987 | 12.86479 | 31.2525 | 39.8839 | 17.81404 | 1.563127569 | 131 | 19 | 0.273209 |
| PPS_3573 | Lipid metabolism | 0 | 1.510512 | 0.71471 | 0 | 3.468165 | 0 | 1.55856972 | 5 | 2 | 0.762962 |
| PPS_4899 | Intracellular trafficking and secretion | 1.187631 | 6.042049 | 2.858842 | 7.29225 | 1.734082 | 6.680264 | 1.556877868 | 27 | 5 | 0.456436 |
| PPS_3331 | General function prediction only | 5.938153 | 1.510512 | 0 | 4.167 | 5.202247 | 2.226755 | 1.55678917 | 16 | 4 | 0.537508 |
| PPS_4332 | Posttranslational modification | 147.2662 | 170.6879 | 175.8188 | 242.7278 | 235.8352 | 289.4781 | 1.555454171 | 1225 | 36 | 0.016934 |
| PPS_0921 | Function unknown | 7.125784 | 1.510512 | 4.288263 | 6.2505 | 10.40449 | 3.340132 | 1.547064528 | 29 | 5 | 0.420643 |
| PPS_4896 | Signal transduction mechanisms | 2.375261 | 1.510512 | 5.002973 | 5.20875 | 5.202247 | 3.340132 | 1.547026787 | 22 | 5 | 0.269751 |
| PPS_4720 | Translation | 3.562892 | 2.265768 | 2.858842 | 8.334 | 1.734082 | 3.340132 | 1.543391197 | 22 | 2 | 0.513194 |
| PPS_3985 | General function prediction only | 1.187631 | 0.755256 | 1.429421 | 0 | 5.202247 | 0 | 1.542637194 | 7 | 3 | 0.759333 |
| PPS_3227 | Signal transduction mechanisms | 0 | 1.510512 | 1.429421 | 1.04175 | 3.468165 | 0 | 1.534019494 | 7 | 2 | 0.678369 |
| PPS_0884 | Translation | 1.187631 | 2.265768 | 0.71471 | 4.167 | 0 | 2.226755 | 1.533969893 | 11 | 3 | 0.611238 |
| PPS_4384 | Function unknown | 8.313414 | 5.286793 | 5.717683 | 12.501 | 10.40449 | 6.680264 | 1.531521124 | 46 | 3 | 0.173274 |
| PPS_0922 | Function unknown | 7.125784 | 7.552562 | 7.861815 | 14.5845 | 12.13858 | 7.793641 | 1.531343061 | 55 | 8 | 0.180766 |
| PPS_1270 |  | 0 | 0.755256 | 1.429421 | 0 | 0 | 3.340132 | 1.52889045 | 6 | 2 | 0.770504 |
| PPS_1486 | Lipid metabolism | 13.06394 | 3.021025 | 4.288263 | 10.4175 | 13.87266 | 6.680264 | 1.520153249 | 45 | 4 | 0.410534 |
| PPS_4734 | Posttranslational modification | 4.750523 | 2.265768 | 8.576525 | 8.334 | 5.202247 | 10.0204 | 1.510736901 | 39 | 14 | 0.319403 |
| PPS_0430 | Posttranslational modification | 1.187631 | 2.265768 | 0 | 5.20875 | 0 | 0 | 1.508296558 | 9 | 4 | 0.776438 |
| PPS_5156 | Intracellular trafficking and secretion | 3.562892 | 4.531537 | 6.432394 | 5.20875 | 12.13858 | 4.453509 | 1.500729813 | 34 | 3 | 0.430936 |
| PPS_3109 | Function unknown | 3.562892 | 5.286793 | 8.576525 | 5.20875 | 8.670412 | 12.24715 | 1.499253815 | 43 | 11 | 0.317734 |
| PPS_0683 | Posttranslational modification | 5.938153 | 3.021025 | 5.002973 | 5.20875 | 3.468165 | 12.24715 | 1.498627622 | 34 | 6 | 0.483852 |
| PPS_0442 | Translation | 68.88258 | 70.23882 | 57.89154 | 98.96625 | 88.4382 | 106.8842 | 1.493752972 | 474 | 8 | 0.010047 |
| PPS_1147 | Translation | 15.4392 | 18.8814 | 17.15305 | 25.002 | 29.4794 | 22.26755 | 1.491033619 | 123 | 18 | 0.039294 |
| PPS_3577 | Secondary metabolites biosynthesis | 5.938153 | 8.307818 | 7.147104 | 13.54275 | 13.87266 | 4.453509 | 1.489683836 | 51 | 9 | 0.37555 |
| PPS_0458 | Translation | 20.18972 | 18.8814 | 20.01189 | 48.96225 | 27.74532 | 11.13377 | 1.48674435 | 143 | 3 | 0.473598 |
| PPS_3594 | Energy production and conversion | 7.125784 | 2.265768 | 3.573552 | 6.2505 | 5.202247 | 7.793641 | 1.48447614 | 30 | 3 | 0.290412 |
| PPS_4352 | Cell division and chromosome partitioning | 23.75261 | 9.063074 | 12.86479 | 25.002 | 26.01124 | 16.70066 | 1.482337833 | 104 | 15 | 0.247585 |
| PPS_4637 | Cell envelope biogenesis | 7.125784 | 1.510512 | 1.429421 | 5.20875 | 5.202247 | 4.453509 | 1.476745905 | 22 | 4 | 0.48625 |
| PPS_0989 | Secondary metabolites biosynthesis | 2.375261 | 2.265768 | 2.144131 | 2.0835 | 3.468165 | 4.453509 | 1.474566906 | 16 | 2 | 0.258139 |
| PPS_1426 | Cell envelope biogenesis | 9.501045 | 8.307818 | 17.15305 | 20.835 | 13.87266 | 16.70066 | 1.470409194 | 86 | 17 | 0.191786 |
| PPS_4388 | Amino acid transport and metabolism | 8.313414 | 6.042049 | 5.717683 | 13.54275 | 10.40449 | 5.566886 | 1.470329023 | 47 | 6 | 0.306969 |
| PPS_4735 | Posttranslational modification | 21.37735 | 9.81833 | 10.72066 | 17.70975 | 26.01124 | 17.81404 | 1.46804386 | 94 | 11 | 0.235522 |
| PPS_0456 | Translation | 4.750523 | 3.776281 | 7.147104 | 9.37575 | 6.93633 | 6.680264 | 1.466918389 | 38 | 4 | 0.13983 |
| PPS_4142 |  | 0 | 1.510512 | 1.429421 | 2.0835 | 0 | 2.226755 | 1.4661063 | 8 | 2 | 0.631126 |
| PPS_3354 | General function prediction only | 2.375261 | 3.021025 | 3.573552 | 5.20875 | 3.468165 | 4.453509 | 1.463841805 | 22 | 5 | 0.09447 |
| PPS_1436 |  | 152.0167 | 106.4911 | 108.636 | 160.4295 | 194.2172 | 182.5939 | 1.463297396 | 851 | 9 | 0.040653 |
| PPS_1276 | General function prediction only | 3.562892 | 1.510512 | 1.429421 | 2.0835 | 5.202247 | 2.226755 | 1.462826026 | 14 | 5 | 0.467016 |
| PPS_1139 | Cell motility and secretion | 5.938153 | 3.021025 | 5.717683 | 8.334 | 8.670412 | 4.453509 | 1.462023849 | 34 | 6 | 0.249838 |
| PPS_2394 | Energy production and conversion | 10.68868 | 6.042049 | 6.432394 | 15.62625 | 10.40449 | 7.793641 | 1.460269044 | 54 | 7 | 0.27538 |
| PPS_3591 | Energy production and conversion | 7.125784 | 11.32884 | 7.147104 | 7.29225 | 15.60674 | 14.4739 | 1.45978008 | 60 | 7 | 0.274443 |
| PPS_4132 | Coenzyme metabolism | 1.187631 | 0 | 2.144131 | 3.12525 | 1.734082 | 0 | 1.458487295 | 8 | 3 | 0.669403 |
| PPS_3372 | Lipid metabolism | 0 | 0 | 2.144131 | 3.12525 | 0 | 0 | 1.457583333 | 6 | 3 | 0.810075 |
| PPS_1442 | Cell envelope biogenesis | 2.375261 | 3.021025 | 4.288263 | 7.29225 | 3.468165 | 3.340132 | 1.4559839 | 24 | 5 | 0.381005 |
| PPS_2036 | Translation | 5.938153 | 9.81833 | 6.432394 | 8.334 | 13.87266 | 10.0204 | 1.452396826 | 52 | 9 | 0.18264 |
| PPS_0031 | Translation | 0 | 3.021025 | 0.71471 | 2.0835 | 0 | 3.340132 | 1.451824529 | 10 | 3 | 0.694972 |
| PPS_4395 | General function prediction only | 1.187631 | 4.531537 | 6.432394 | 6.2505 | 6.93633 | 4.453509 | 1.451693168 | 30 | 3 | 0.364076 |
| PPS_1194 | Cell envelope biogenesis | 15.4392 | 6.042049 | 6.432394 | 11.45925 | 15.60674 | 13.36053 | 1.448271059 | 62 | 13 | 0.307261 |
| PPS_1433 | Amino acid transport and metabolism | 10.68868 | 12.0841 | 11.43537 | 17.70975 | 17.34082 | 14.4739 | 1.447739544 | 81 | 10 | 0.025476 |
| PPS_1441 | Cell envelope biogenesis | 27.3155 | 15.10512 | 17.15305 | 30.21075 | 31.21348 | 24.4943 | 1.442223079 | 136 | 16 | 0.13117 |
| PPS_1696 | General function prediction only | 5.938153 | 6.042049 | 5.717683 | 5.20875 | 6.93633 | 13.36053 | 1.441166877 | 42 | 14 | 0.404097 |
| PPS_0846 | Carbohydrate transport and metabolism | 1.187631 | 0.755256 | 1.429421 | 3.12525 | 1.734082 | 0 | 1.440951693 | 8 | 4 | 0.64162 |
| PPS_0461 | Translation | 30.8784 | 15.10512 | 17.15305 | 31.2525 | 32.94757 | 26.72105 | 1.440070635 | 143 | 11 | 0.194062 |
| PPS_4311 | Cell envelope biogenesis | 0 | 1.510512 | 0.71471 | 2.0835 | 0 | 1.113377 | 1.436654948 | 6 | 3 | 0.6876 |
| PPS_0004 | Cell division and chromosome partitioning | 1.187631 | 0.755256 | 0.71471 | 2.0835 | 1.734082 | 0 | 1.436478928 | 6 | 3 | 0.612934 |
| PPS_1780 |  | 2.375261 | 4.531537 | 4.288263 | 4.167 | 5.202247 | 6.680264 | 1.43362427 | 27 | 8 | 0.180696 |
| PPS_4351 | Cell division and chromosome partitioning | 17.81446 | 13.59461 | 13.5795 | 23.96025 | 26.01124 | 14.4739 | 1.432483691 | 103 | 13 | 0.201649 |
| PPS_1081 | Cell envelope biogenesis | 0 | 0.755256 | 1.429421 | 3.12525 | 0 | 0 | 1.430531831 | 6 | 4 | 0.800293 |
| PPS_3786 | Cell motility and secretion | 0 | 0.755256 | 1.429421 | 3.12525 | 0 | 0 | 1.430531831 | 6 | 4 | 0.800293 |
| PPS_4577 | Translation | 4.750523 | 2.265768 | 2.144131 | 6.2505 | 3.468165 | 3.340132 | 1.4255671 | 21 | 3 | 0.36603 |
| PPS_4526 | Cell envelope biogenesis | 1.187631 | 1.510512 | 5.002973 | 4.167 | 3.468165 | 3.340132 | 1.425156657 | 19 | 9 | 0.467617 |
| PPS_1196 | 3-hydroxymyristoyl | 1.187631 | 1.510512 | 4.288263 | 3.12525 | 3.468165 | 3.340132 | 1.421839403 | 17 | 5 | 0.423505 |
| PPS_3512 | Energy production and conversion | 7.125784 | 2.265768 | 10.72066 | 11.45925 | 10.40449 | 6.680264 | 1.419237866 | 47 | 8 | 0.391193 |
| PPS_0918 | Function unknown | 4.750523 | 0 | 2.144131 | 0 | 8.670412 | 1.113377 | 1.419039964 | 13 | 2 | 0.773196 |
| PPS_4382 | Translation | 7.125784 | 9.063074 | 7.861815 | 10.4175 | 19.07491 | 4.453509 | 1.411433116 | 54 | 8 | 0.519275 |
| PPS_3253 |  | 4.750523 | 1.510512 | 3.573552 | 4.167 | 5.202247 | 4.453509 | 1.405524842 | 22 | 3 | 0.293607 |
| PPS_0227 |  | 19.00209 | 21.14717 | 24.30015 | 31.2525 | 19.07491 | 40.08158 | 1.402789852 | 155 | 18 | 0.289117 |
| PPS_5062 | Coenzyme metabolism | 2.375261 | 3.776281 | 2.858842 | 5.20875 | 5.202247 | 2.226755 | 1.402576413 | 21 | 3 | 0.351534 |
| PPS_4167 | Amino acid transport and metabolism | 24.94024 | 19.63666 | 20.7266 | 34.37775 | 38.14981 | 18.92741 | 1.400460441 | 148 | 16 | 0.273627 |
| PPS_0466 | Translation | 13.06394 | 5.286793 | 9.291235 | 10.4175 | 19.07491 | 8.907018 | 1.389171275 | 60 | 7 | 0.413307 |
| PPS_0089 | General function prediction only | 1.187631 | 1.510512 | 2.858842 | 3.12525 | 3.468165 | 1.113377 | 1.38686582 | 13 | 4 | 0.473275 |
| PPS_5275 | Energy production and conversion | 80.75888 | 66.46254 | 51.45915 | 95.841 | 93.64045 | 83.5033 | 1.373988092 | 449 | 19 | 0.082178 |
| PPS_0446 | Translation | 51.06812 | 27.94448 | 31.44726 | 37.503 | 65.89513 | 47.87522 | 1.369487182 | 241 | 10 | 0.284346 |
| PPS_0686 | Coenzyme metabolism | 3.562892 | 6.042049 | 6.432394 | 8.334 | 6.93633 | 6.680264 | 1.368718258 | 38 | 5 | 0.147714 |
| PPS_1659 | Nucleotide transport and metabolism | 7.125784 | 4.531537 | 5.717683 | 12.501 | 3.468165 | 7.793641 | 1.367643168 | 41 | 11 | 0.504638 |
| PPS_2035 | Translation | 9.501045 | 3.776281 | 5.002973 | 11.45925 | 3.468165 | 10.0204 | 1.364737552 | 42 | 3 | 0.505843 |
| PPS_1361 | Cell envelope biogenesis | 7.125784 | 11.32884 | 7.147104 | 15.62625 | 10.40449 | 8.907018 | 1.364664105 | 60 | 15 | 0.284312 |
| PPS_0469 | Translation | 9.501045 | 8.307818 | 8.576525 | 14.5845 | 6.93633 | 14.4739 | 1.364191964 | 62 | 5 | 0.332675 |
| PPS_2455 | Transcription | 0 | 0 | 2.858842 | 1.04175 | 1.734082 | 1.113377 | 1.360414493 | 7 | 3 | 0.756108 |
| PPS_1538 | Translation | 28.50314 | 22.65768 | 22.87073 | 38.54475 | 36.41573 | 25.60768 | 1.358449915 | 167 | 25 | 0.144499 |
| PPS_0452 | Translation | 16.62683 | 19.63666 | 12.86479 | 12.501 | 32.94757 | 21.15417 | 1.355690414 | 108 | 6 | 0.433748 |
| PPS_0450 | Translation | 16.62683 | 30.9655 | 30.73255 | 25.002 | 39.8839 | 41.19496 | 1.354369833 | 182 | 9 | 0.25914 |
| PPS_3783 | Cell motility and secretion | 2.375261 | 0.755256 | 0 | 3.12525 | 0 | 1.113377 | 1.353970192 | 7 | 3 | 0.765584 |
| PPS_1290 | Energy production and conversion | 19.00209 | 17.37089 | 15.72363 | 20.835 | 29.4794 | 20.04079 | 1.350475399 | 116 | 12 | 0.173487 |
| PPS_4694 | Posttranslational modification | 2.375261 | 0 | 0 | 2.0835 | 0 | 1.113377 | 1.345905534 | 5 | 2 | 0.797599 |
| PPS_1495 | Translation | 10.68868 | 12.0841 | 8.576525 | 14.5845 | 8.670412 | 18.92741 | 1.3455588 | 73 | 7 | 0.349766 |
| PPS_1600 |  | 10.68868 | 14.34987 | 13.5795 | 19.79325 | 24.27715 | 7.793641 | 1.343000406 | 87 | 13 | 0.466293 |
| PPS_0440 | Translation | 97.38571 | 107.2464 | 121.5008 | 131.2605 | 119.6517 | 187.0474 | 1.342886973 | 757 | 20 | 0.208197 |
| PPS_4990 | Cell envelope biogenesis | 2.375261 | 1.510512 | 0 | 5.20875 | 0 | 0 | 1.340466677 | 9 | 2 | 0.83074 |
| PPS_4574 | Posttranslational modification | 8.313414 | 1.510512 | 7.861815 | 8.334 | 8.670412 | 6.680264 | 1.339196085 | 39 | 6 | 0.462127 |
| PPS_4851 | Function unknown | 10.68868 | 6.797305 | 7.147104 | 8.334 | 15.60674 | 8.907018 | 1.333481343 | 53 | 7 | 0.375544 |
| PPS_1534 | Function unknown | 3.562892 | 1.510512 | 5.002973 | 3.12525 | 6.93633 | 3.340132 | 1.330012878 | 22 | 3 | 0.527549 |
| PPS_4532 | Inorganic ion transport and metabolism | 13.06394 | 9.063074 | 5.002973 | 15.62625 | 6.93633 | 13.36053 | 1.324110883 | 61 | 9 | 0.448953 |
| PPS_3418 | Posttranslational modification | 13.06394 | 15.10512 | 9.291235 | 11.45925 | 19.07491 | 18.92741 | 1.320373187 | 83 | 17 | 0.267005 |
| PPS_1362 | Energy production and conversion | 5.938153 | 1.510512 | 4.288263 | 5.20875 | 6.93633 | 3.340132 | 1.319358131 | 25 | 6 | 0.494722 |
| PPS_3517 | Energy production and conversion | 4.750523 | 4.531537 | 2.144131 | 4.167 | 5.202247 | 5.566886 | 1.307183983 | 25 | 3 | 0.300506 |
| PPS_4327 |  | 1.187631 | 0.755256 | 0.71471 | 0 | 3.468165 | 0 | 1.305000162 | 5 | 2 | 0.837657 |
| PPS_3916 | Inorganic ion transport and metabolism | 144.8909 | 136.7014 | 126.5037 | 176.0558 | 175.1423 | 180.3671 | 1.302549226 | 912 | 35 | 0.010954 |
| PPS_4689 | General function prediction only | 3.562892 | 6.797305 | 7.861815 | 3.12525 | 13.87266 | 6.680264 | 1.299426913 | 40 | 3 | 0.635724 |
| PPS_4522 | Amino acid transport and metabolism | 8.313414 | 12.0841 | 12.86479 | 19.79325 | 12.13858 | 11.13377 | 1.294727024 | 77 | 6 | 0.366097 |
| PPS_4891 | Signal transduction mechanisms | 10.68868 | 12.83935 | 16.43834 | 7.29225 | 24.27715 | 20.04079 | 1.291340551 | 88 | 15 | 0.533323 |
| PPS_0970 | General function prediction only | 5.938153 | 3.021025 | 4.288263 | 6.2505 | 5.202247 | 5.566886 | 1.284748836 | 29 | 7 | 0.272141 |
| PPS_1548 | Lipid metabolism | 30.8784 | 14.34987 | 17.15305 | 20.835 | 22.54307 | 36.74145 | 1.284351299 | 135 | 4 | 0.456275 |
|  | Transcription | 5.938153 | 6.042049 | 5.002973 | 10.4175 | 6.93633 | 4.453509 | 1.284055435 | 38 | 7 | 0.451864 |
| PPS_1116 | Translation | 16.62683 | 16.61564 | 16.43834 | 19.79325 | 29.4794 | 14.4739 | 1.283122454 | 108 | 7 | 0.39756 |
| PPS_4670 | Coenzyme metabolism | 2.375261 | 0 | 0.71471 | 0 | 1.734082 | 2.226755 | 1.281836 | 6 | 2 | 0.780799 |
| PPS_1965 | General function prediction only | 2.375261 | 2.265768 | 1.429421 | 2.0835 | 3.468165 | 2.226755 | 1.28135782 | 13 | 2 | 0.351946 |
| PPS_4564 | Posttranslational modification | 24.94024 | 17.37089 | 18.58247 | 29.169 | 24.27715 | 24.4943 | 1.279944788 | 134 | 16 | 0.125355 |
| PPS_0969 | General function prediction only | 0 | 1.510512 | 0.71471 | 0 | 1.734082 | 1.113377 | 1.279629049 | 5 | 3 | 0.772368 |
| PPS_0022 | Amino acid transport and metabolism | 1.187631 | 0 | 2.144131 | 3.12525 | 0 | 1.113377 | 1.27218793 | 8 | 2 | 0.799707 |
| PPS_1425 | Cell envelope biogenesis | 13.06394 | 11.32884 | 12.15008 | 15.62625 | 20.80899 | 10.0204 | 1.271264444 | 79 | 7 | 0.400251 |
| PPS_1699 | Lipid metabolism | 16.62683 | 15.86038 | 17.15305 | 18.7515 | 24.27715 | 20.04079 | 1.270530141 | 109 | 18 | 0.109032 |
| PPS_3006 | Translation | 3.562892 | 8.307818 | 9.291235 | 10.4175 | 8.670412 | 7.793641 | 1.270277976 | 49 | 12 | 0.402262 |
| PPS_0790 | Translation | 0 | 3.021025 | 1.429421 | 1.04175 | 3.468165 | 1.113377 | 1.263534644 | 10 | 4 | 0.757525 |
| PPS_1653 | Cell envelope biogenesis | 39.19181 | 27.94448 | 55.0327 | 50.004 | 38.14981 | 65.68926 | 1.259264484 | 276 | 17 | 0.399066 |
| PPS_0473 | Translation | 5.938153 | 6.042049 | 8.576525 | 10.4175 | 8.670412 | 6.680264 | 1.253515453 | 46 | 6 | 0.280205 |
| PPS_4569 | Amino acid transport and metabolism | 48.69286 | 47.58114 | 38.59436 | 58.338 | 55.49064 | 54.55549 | 1.248507263 | 295 | 36 | 0.059754 |
| PPS_4036 | Nucleotide transport and metabolism | 10.68868 | 6.042049 | 10.00595 | 9.37575 | 13.87266 | 10.0204 | 1.244313653 | 57 | 9 | 0.341214 |
| PPS_5108 | DNA replication | 1.187631 | 3.021025 | 4.288263 | 3.12525 | 5.202247 | 2.226755 | 1.242127082 | 19 | 4 | 0.615063 |
| PPS_1644 | Amino acid transport and metabolism | 20.18972 | 17.37089 | 14.29421 | 18.7515 | 27.74532 | 17.81404 | 1.240209755 | 110 | 24 | 0.330038 |
| PPS_1121 | Amino acid transport and metabolism | 21.37735 | 12.0841 | 17.15305 | 22.9185 | 20.80899 | 18.92741 | 1.237884446 | 109 | 15 | 0.272259 |
| PPS_0977 | General function prediction only | 4.750523 | 2.265768 | 2.144131 | 1.04175 | 6.93633 | 3.340132 | 1.23555564 | 18 | 2 | 0.73267 |
| PPS_4956 | Intracellular trafficking and secretion | 2.375261 | 3.021025 | 2.144131 | 3.12525 | 1.734082 | 4.453509 | 1.235056531 | 17 | 8 | 0.537498 |
| PPS_0464 | Translation | 27.3155 | 15.10512 | 24.30015 | 29.169 | 17.34082 | 35.62807 | 1.231069152 | 147 | 13 | 0.478271 |
| PPS_1180 | Amino acid transport and metabolism | 15.4392 | 16.61564 | 12.15008 | 14.5845 | 20.80899 | 18.92741 | 1.228843159 | 95 | 8 | 0.219311 |
| PPS_4012 | Translation | 28.50314 | 21.90243 | 18.58247 | 22.9185 | 38.14981 | 23.38092 | 1.224114235 | 144 | 21 | 0.434874 |
| PPS_1883 | Posttranslational modification | 24.94024 | 24.1682 | 24.30015 | 20.835 | 43.35206 | 25.60768 | 1.223218309 | 155 | 16 | 0.50892 |
| PPS_4322 | General function prediction only | 9.501045 | 7.552562 | 8.576525 | 7.29225 | 17.34082 | 6.680264 | 1.221739238 | 53 | 12 | 0.640284 |
| PPS_4107 | Signal transduction mechanisms | 1.187631 | 0 | 1.429421 | 2.0835 | 0 | 1.113377 | 1.221556894 | 6 | 2 | 0.8096 |
| PPS_0284 | Amino acid transport and metabolism | 0 | 2.265768 | 1.429421 | 1.04175 | 3.468165 | 0 | 1.220482738 | 8 | 4 | 0.836765 |
| PPS_2742 | Transcription | 2.375261 | 1.510512 | 2.144131 | 1.04175 | 5.202247 | 1.113377 | 1.220147688 | 12 | 3 | 0.779999 |
| PPS_1464 | Coenzyme metabolism | 0 | 0.755256 | 3.573552 | 4.167 | 0 | 1.113377 | 1.219822394 | 11 | 4 | 0.85738 |
| PPS_4387 | Inorganic ion transport and metabolism | 21.37735 | 23.41294 | 27.87371 | 26.04375 | 34.68165 | 27.83443 | 1.218757998 | 158 | 17 | 0.185722 |
| PPS_3929 | General function prediction only | 8.313414 | 6.797305 | 4.288263 | 9.37575 | 8.670412 | 5.566886 | 1.217231288 | 41 | 7 | 0.444381 |
| PPS_4646 | Cell envelope biogenesis | 2.375261 | 1.510512 | 2.858842 | 3.12525 | 1.734082 | 3.340132 | 1.215705252 | 15 | 3 | 0.49272 |
| PPS_0239 | Signal transduction mechanisms | 4.750523 | 1.510512 | 3.573552 | 6.2505 | 3.468165 | 2.226755 | 1.214633557 | 21 | 2 | 0.668732 |
| PPS_5153 |  | 0 | 2.265768 | 2.144131 | 3.12525 | 0 | 2.226755 | 1.21363406 | 11 | 2 | 0.80476 |
| PPS_5020 | Inorganic ion transport and metabolism | 8.313414 | 4.531537 | 5.002973 | 7.29225 | 12.13858 | 2.226755 | 1.213450983 | 36 | 4 | 0.712584 |
| PPS_4811 | Coenzyme metabolism | 17.81446 | 16.61564 | 11.43537 | 15.62625 | 27.74532 | 12.24715 | 1.212649261 | 95 | 11 | 0.57392 |
| PPS_4544 | Coenzyme metabolism | 3.562892 | 0 | 2.858842 | 2.0835 | 3.468165 | 2.226755 | 1.211264722 | 13 | 4 | 0.72921 |
|  |  | 36.81655 | 31.72076 | 40.02378 | 47.9205 | 43.35206 | 40.08158 | 1.209955973 | 236 | 39 | 0.084146 |
| PPS_4718 | Translation | 39.19181 | 64.19677 | 44.31205 | 35.4195 | 65.89513 | 76.82303 | 1.206072458 | 321 | 9 | 0.531181 |
| PPS_3257 | Inorganic ion transport and metabolism | 10.68868 | 12.0841 | 6.432394 | 6.2505 | 15.60674 | 13.36053 | 1.205874548 | 61 | 7 | 0.58227 |
| PPS_4341 | Function unknown | 5.938153 | 6.042049 | 7.147104 | 8.334 | 6.93633 | 7.793641 | 1.205813808 | 42 | 4 | 0.079787 |
| PPS_4558 | Translation | 42.7547 | 50.60216 | 49.31502 | 48.96225 | 62.42697 | 60.12237 | 1.202140067 | 309 | 21 | 0.133461 |
| PPS_4379 | Energy production and conversion | 4.750523 | 6.797305 | 5.002973 | 6.2505 | 6.93633 | 6.680264 | 1.200370501 | 36 | 7 | 0.222763 |
| PPS_4997 | Amino acid transport and metabolism | 7.125784 | 2.265768 | 1.429421 | 2.0835 | 8.670412 | 2.226755 | 1.19958403 | 20 | 8 | 0.810632 |
| PPS_0240 | Signal transduction mechanisms | 0 | 1.510512 | 2.144131 | 1.04175 | 0 | 3.340132 | 1.19899019 | 9 | 5 | 0.848104 |
| PPS_2886 | Signal transduction mechanisms | 1.187631 | 0 | 1.429421 | 3.12525 | 0 | 0 | 1.19418744 | 6 | 2 | 0.891478 |
| PPS_1884 | Posttranslational modification | 30.8784 | 23.41294 | 27.159 | 36.46125 | 45.08614 | 15.58728 | 1.192563247 | 170 | 26 | 0.615084 |
| PPS_3687 | Transcription | 4.750523 | 1.510512 | 1.429421 | 5.20875 | 1.734082 | 2.226755 | 1.192333369 | 16 | 5 | 0.765017 |
| PPS_1187 | Translation | 53.44338 | 68.72831 | 61.4651 | 66.672 | 64.16105 | 87.9568 | 1.191427157 | 402 | 19 | 0.266837 |
| PPS_3611 | Cell envelope biogenesis | 10.68868 | 4.531537 | 5.717683 | 8.334 | 12.13858 | 4.453509 | 1.190477106 | 42 | 6 | 0.67227 |
| PPS_1112 | Intracellular trafficking and secretion | 4.750523 | 10.57359 | 7.861815 | 7.29225 | 6.93633 | 13.36053 | 1.189907614 | 52 | 15 | 0.614117 |
| PPS_4476 | Energy production and conversion | 3.562892 | 2.265768 | 3.573552 | 8.334 | 1.734082 | 1.113377 | 1.189237074 | 21 | 7 | 0.822992 |
| PPS_1186 | Translation | 38.00418 | 34.74178 | 30.01784 | 42.71175 | 41.61798 | 37.85483 | 1.188984381 | 219 | 15 | 0.089677 |
| PPS_4963 | Inorganic ion transport and metabolism | 5.938153 | 1.510512 | 8.576525 | 9.37575 | 5.202247 | 4.453509 | 1.187599375 | 35 | 5 | 0.717822 |
| PPS_3242 |  | 1.187631 | 3.776281 | 0.71471 | 1.04175 | 3.468165 | 2.226755 | 1.186321171 | 12 | 5 | 0.781439 |
| PPS_0614 | General function prediction only | 9.501045 | 7.552562 | 4.288263 | 10.4175 | 10.40449 | 4.453509 | 1.184315359 | 44 | 6 | 0.629599 |
| PPS_1193 | Cell envelope biogenesis | 2.375261 | 0.755256 | 2.858842 | 3.12525 | 1.734082 | 2.226755 | 1.183112719 | 13 | 5 | 0.657838 |
| PPS_0920 | Translation | 10.68868 | 6.797305 | 6.432394 | 8.334 | 12.13858 | 7.793641 | 1.181778351 | 49 | 13 | 0.494429 |
| PPS_1146 | Translation | 1.187631 | 4.531537 | 8.576525 | 8.334 | 5.202247 | 3.340132 | 1.180521951 | 33 | 6 | 0.758181 |
| PPS_0462 | Translation | 27.3155 | 15.10512 | 32.87668 | 25.002 | 38.14981 | 25.60768 | 1.178787042 | 158 | 10 | 0.545365 |
| PPS_4559 | Transcription | 28.50314 | 28.69973 | 30.01784 | 26.04375 | 43.35206 | 33.40132 | 1.178586274 | 184 | 15 | 0.409387 |
| PPS_4381 | General function prediction only | 3.562892 | 3.021025 | 2.144131 | 5.20875 | 1.734082 | 3.340132 | 1.178151683 | 19 | 5 | 0.669553 |
| PPS_4224 | Coenzyme metabolism | 3.562892 | 5.286793 | 1.429421 | 4.167 | 3.468165 | 4.453509 | 1.176043328 | 22 | 5 | 0.647535 |
| PPS_4597 | Energy production and conversion | 1.187631 | 0.755256 | 0.71471 | 3.12525 | 0 | 0 | 1.175968271 | 6 | 2 | 0.89537 |
| PPS_4170 | DNA replication | 2.375261 | 0.755256 | 1.429421 | 3.12525 | 0 | 2.226755 | 1.173701097 | 10 | 3 | 0.816379 |
| PPS_0555 | Lipid metabolism | 23.75261 | 17.37089 | 17.86776 | 17.70975 | 34.68165 | 16.70066 | 1.171225217 | 120 | 18 | 0.630994 |
| PPS_4724 | Inorganic ion transport and metabolism | 15.4392 | 12.0841 | 12.15008 | 14.5845 | 17.34082 | 14.4739 | 1.169530692 | 83 | 12 | 0.199165 |
| PPS_0993 | Amino acid transport and metabolism | 9.501045 | 5.286793 | 5.002973 | 9.37575 | 10.40449 | 3.340132 | 1.168237929 | 40 | 9 | 0.698947 |
| PPS_1611 | DNA replication | 4.750523 | 1.510512 | 0.71471 | 4.167 | 1.734082 | 2.226755 | 1.165156787 | 14 | 7 | 0.805659 |
| PPS_1580 | Cell motility and secretion | 13.06394 | 7.552562 | 10.72066 | 4.167 | 15.60674 | 16.70066 | 1.163934658 | 64 | 12 | 0.721524 |
| PPS_0475 | Transcription | 52.25575 | 64.19677 | 57.89154 | 54.171 | 69.3633 | 77.93641 | 1.155592558 | 372 | 17 | 0.329851 |
| PPS_0001 | Transcription | 2.375261 | 2.265768 | 0.71471 | 0 | 1.734082 | 4.453509 | 1.15531958 | 11 | 4 | 0.857384 |
| PPS_4823 |  | 7.125784 | 5.286793 | 9.291235 | 6.2505 | 12.13858 | 6.680264 | 1.155066213 | 45 | 13 | 0.645156 |
| PPS_1574 | Amino acid transport and metabolism | 3.562892 | 2.265768 | 4.288263 | 3.12525 | 5.202247 | 3.340132 | 1.153278434 | 21 | 9 | 0.591234 |
| PPS_4644 | Function unknown | 1.187631 | 0.755256 | 1.429421 | 1.04175 | 1.734082 | 1.113377 | 1.153278434 | 7 | 2 | 0.591234 |
| PPS_5131 | General function prediction only | 4.750523 | 0.755256 | 2.144131 | 3.12525 | 3.468165 | 2.226755 | 1.152976885 | 15 | 4 | 0.776373 |
| PPS_3303 | Cell envelope biogenesis | 10.68868 | 18.12615 | 12.86479 | 16.668 | 15.60674 | 15.58728 | 1.148331811 | 90 | 11 | 0.4499 |
| PPS_3007 | Translation | 8.313414 | 4.531537 | 9.291235 | 7.29225 | 6.93633 | 11.13377 | 1.145741697 | 47 | 13 | 0.615688 |
| PPS_3096 | Transcription | 3.562892 | 2.265768 | 0 | 2.0835 | 3.468165 | 1.113377 | 1.143494669 | 11 | 2 | 0.835459 |
| PPS_0156 |  | 4.750523 | 0 | 0 | 2.0835 | 0 | 3.340132 | 1.141691634 | 9 | 2 | 0.91087 |
| PPS_3679 | Cell division and chromosome partitioning | 19.00209 | 8.307818 | 7.147104 | 16.668 | 10.40449 | 12.24715 | 1.141121705 | 70 | 11 | 0.726342 |
| PPS_0124 | Energy production and conversion | 4.750523 | 3.776281 | 2.858842 | 7.29225 | 3.468165 | 2.226755 | 1.140661712 | 24 | 5 | 0.767223 |
| PPS_1050 | Transcription | 3.562892 | 0.755256 | 2.144131 | 1.04175 | 5.202247 | 1.113377 | 1.138510749 | 12 | 4 | 0.862817 |
| PPS_4904 | Cell envelope biogenesis | 7.125784 | 6.042049 | 5.717683 | 6.2505 | 5.202247 | 10.0204 | 1.137016437 | 40 | 8 | 0.621026 |
| PPS_0331 | Posttranslational modification | 2.375261 | 3.021025 | 2.144131 | 5.20875 | 0 | 3.340132 | 1.133741226 | 17 | 3 | 0.847023 |
| PPS_1646 | Carbohydrate transport and metabolism | 10.68868 | 6.042049 | 5.717683 | 8.334 | 10.40449 | 6.680264 | 1.132318943 | 45 | 5 | 0.639224 |
| PPS_0459 | Translation | 11.87631 | 10.57359 | 10.00595 | 10.4175 | 17.34082 | 8.907018 | 1.129699427 | 66 | 4 | 0.646008 |
| PPS_4184 | Amino acid transport and metabolism | 9.501045 | 11.32884 | 15.72363 | 9.37575 | 17.34082 | 14.4739 | 1.126854052 | 77 | 9 | 0.631857 |
| PPS_0010 | DNA replication | 10.68868 | 8.307818 | 5.717683 | 7.29225 | 13.87266 | 6.680264 | 1.126688257 | 49 | 9 | 0.723612 |
| PPS_0467 | Translation | 32.06603 | 38.51806 | 40.02378 | 56.2545 | 26.01124 | 42.30834 | 1.126267658 | 241 | 9 | 0.652819 |
| PPS_5201 | DNA replication | 2.375261 | 1.510512 | 3.573552 | 2.0835 | 5.202247 | 1.113377 | 1.125989775 | 15 | 5 | 0.834446 |
| PPS_3894 |  | 1.187631 | 3.021025 | 5.717683 | 3.12525 | 6.93633 | 1.113377 | 1.125788394 | 21 | 4 | 0.856878 |
| PPS_4554 | Translation | 39.19181 | 34.74178 | 34.3061 | 33.336 | 41.61798 | 46.76185 | 1.124502646 | 225 | 24 | 0.374343 |
| PPS_3616 |  | 13.06394 | 6.042049 | 11.43537 | 10.4175 | 13.87266 | 10.0204 | 1.123413054 | 62 | 10 | 0.641332 |
| PPS_3915 | Function unknown | 8.313414 | 2.265768 | 5.002973 | 8.334 | 6.93633 | 2.226755 | 1.122892384 | 31 | 3 | 0.814254 |
| PPS_0400 | Cell envelope biogenesis | 26.12787 | 12.83935 | 17.15305 | 27.0855 | 22.54307 | 13.36053 | 1.122394611 | 114 | 19 | 0.704724 |
| PPS_0687 | Translation | 33.25366 | 52.11268 | 46.45618 | 43.7535 | 45.08614 | 59.009 | 1.121573526 | 283 | 6 | 0.511919 |
| PPS_3254 | Inorganic ion transport and metabolism | 4.750523 | 3.021025 | 5.002973 | 4.167 | 3.468165 | 6.680264 | 1.120623576 | 27 | 5 | 0.683915 |
| PPS_4556 | Translation | 3.562892 | 1.510512 | 1.429421 | 2.0835 | 5.202247 | 0 | 1.120397232 | 12 | 3 | 0.886007 |
| PPS_5273 | Energy production and conversion | 104.5115 | 179.751 | 143.6568 | 123.9683 | 168.206 | 187.0474 | 1.119887961 | 911 | 37 | 0.58355 |
| PPS_5172 | Signal transduction mechanisms | 2.375261 | 0.755256 | 1.429421 | 0 | 1.734082 | 3.340132 | 1.112781339 | 9 | 3 | 0.883527 |
| PPS_4126 | Amino acid transport and metabolism | 3.562892 | 0.755256 | 1.429421 | 4.167 | 0 | 2.226755 | 1.112427647 | 12 | 2 | 0.891471 |
| PPS_1408 | DNA replication | 14.25157 | 10.57359 | 15.72363 | 20.835 | 8.670412 | 15.58728 | 1.112060346 | 87 | 21 | 0.722101 |
| PPS_0666 | Translation | 4.750523 | 0.755256 | 2.144131 | 6.2505 | 0 | 2.226755 | 1.108150894 | 16 | 3 | 0.906093 |
| PPS_2935 | Defense mechanisms | 9.501045 | 6.042049 | 4.288263 | 3.12525 | 12.13858 | 6.680264 | 1.106534997 | 38 | 9 | 0.83051 |
| PPS_2056 | Amino acid transport and metabolism | 1.187631 | 2.265768 | 4.288263 | 5.20875 | 0 | 3.340132 | 1.104269626 | 18 | 7 | 0.88834 |
| PPS_0438 | Transcription | 14.25157 | 8.307818 | 11.43537 | 12.501 | 13.87266 | 11.13377 | 1.103330061 | 69 | 5 | 0.582113 |
| PPS_5151 | Signal transduction mechanisms | 3.562892 | 2.265768 | 2.858842 | 1.04175 | 5.202247 | 3.340132 | 1.103208832 | 17 | 8 | 0.831398 |
| PPS_1438 | General function prediction only | 4.750523 | 2.265768 | 1.429421 | 3.12525 | 1.734082 | 4.453509 | 1.102670994 | 17 | 6 | 0.831609 |
| PPS_0121 | Energy production and conversion | 13.06394 | 5.286793 | 8.576525 | 11.45925 | 10.40449 | 7.793641 | 1.1013891 | 54 | 6 | 0.741203 |
| PPS_2038 | DNA replication | 3.562892 | 5.286793 | 3.573552 | 6.2505 | 5.202247 | 2.226755 | 1.101122162 | 26 | 4 | 0.775144 |
| PPS_5184 | Nucleotide transport and metabolism | 10.68868 | 8.307818 | 2.144131 | 7.29225 | 10.40449 | 5.566886 | 1.100423051 | 41 | 5 | 0.823115 |
| PPS_0904 | Carbohydrate transport and metabolism | 7.125784 | 12.83935 | 10.72066 | 9.37575 | 12.13858 | 12.24715 | 1.100231456 | 65 | 7 | 0.627704 |
| PPS_3609 | Cell envelope biogenesis | 4.750523 | 3.776281 | 4.288263 | 2.0835 | 8.670412 | 3.340132 | 1.099802681 | 25 | 7 | 0.853069 |
| PPS_0562 | Function unknown | 9.501045 | 5.286793 | 2.858842 | 6.2505 | 8.670412 | 4.453509 | 1.097907438 | 34 | 5 | 0.816238 |
| PPS_4320 | Inorganic ion transport and metabolism | 58.1939 | 33.98653 | 40.73849 | 60.4215 | 52.02247 | 33.40132 | 1.09725002 | 269 | 21 | 0.709472 |
| PPS_4914 | Posttranslational modification | 0 | 2.265768 | 0.71471 | 1.04175 | 0 | 2.226755 | 1.096637366 | 7 | 2 | 0.922579 |
| PPS_0534 | Energy production and conversion | 20.18972 | 16.61564 | 12.15008 | 17.70975 | 22.54307 | 13.36053 | 1.095146026 | 98 | 10 | 0.682912 |
| PPS_4358 | Cell envelope biogenesis | 0 | 2.265768 | 6.432394 | 2.0835 | 5.202247 | 2.226755 | 1.093622011 | 19 | 9 | 0.90693 |
| PPS_2581 | Posttranslational modification | 2.375261 | 3.021025 | 0 | 4.167 | 1.734082 | 0 | 1.093545171 | 11 | 4 | 0.917437 |
| PPS_3586 | Energy production and conversion | 27.3155 | 36.2523 | 46.45618 | 34.37775 | 46.82022 | 38.9682 | 1.092181738 | 231 | 16 | 0.640245 |
| PPS_0776 | Intracellular trafficking and secretion | 4.750523 | 6.797305 | 2.144131 | 4.167 | 5.202247 | 5.566886 | 1.090868975 | 28 | 9 | 0.792375 |
| PPS_4117 | Transcription | 8.313414 | 3.021025 | 0.71471 | 5.20875 | 3.468165 | 4.453509 | 1.08973865 | 23 | 12 | 0.888934 |
| PPS_3925 | Secondary metabolites biosynthesis | 15.4392 | 15.10512 | 20.01189 | 19.79325 | 20.80899 | 14.4739 | 1.089404046 | 105 | 14 | 0.583893 |
| PPS_1913 |  | 4.750523 | 3.021025 | 0.71471 | 4.167 | 1.734082 | 3.340132 | 1.088962256 | 17 | 4 | 0.864935 |
| PPS_5276 | Energy production and conversion | 9.501045 | 7.552562 | 20.7266 | 15.62625 | 12.13858 | 13.36053 | 1.088542261 | 81 | 3 | 0.814348 |
| PPS_4742 | Carbohydrate transport and metabolism | 1.187631 | 0 | 1.429421 | 0 | 1.734082 | 1.113377 | 1.08804114 | 5 | 3 | 0.914695 |
| PPS_0397 | Translation | 1.187631 | 0 | 1.429421 | 0 | 1.734082 | 1.113377 | 1.08804114 | 5 | 2 | 0.914695 |
| PPS_1467 | Secondary metabolites biosynthesis | 1.187631 | 1.510512 | 2.858842 | 2.0835 | 1.734082 | 2.226755 | 1.087700853 | 12 | 4 | 0.785434 |
| PPS_3948 | Energy production and conversion | 11.87631 | 3.021025 | 10.00595 | 9.37575 | 12.13858 | 5.566886 | 1.087455811 | 49 | 5 | 0.837784 |
| PPS_3409 | Coenzyme metabolism | 2.375261 | 1.510512 | 0.71471 | 1.04175 | 1.734082 | 2.226755 | 1.087404485 | 9 | 5 | 0.832498 |
| PPS_0251 | Function unknown | 0 | 1.510512 | 1.429421 | 2.0835 | 0 | 1.113377 | 1.087397939 | 7 | 2 | 0.917689 |
| PPS_0314 | Amino acid transport and metabolism | 0 | 1.510512 | 1.429421 | 2.0835 | 0 | 1.113377 | 1.087397939 | 7 | 4 | 0.917689 |
| PPS_0472 | Translation | 20.18972 | 13.59461 | 15.72363 | 14.5845 | 22.54307 | 16.70066 | 1.087264134 | 99 | 7 | 0.664608 |
| PPS_1957 | Inorganic ion transport and metabolism | 22.56498 | 18.12615 | 14.29421 | 16.668 | 20.80899 | 22.26755 | 1.086553908 | 111 | 18 | 0.618866 |
| PPS_3817 | Amino acid transport and metabolism | 26.12787 | 15.10512 | 17.15305 | 21.87675 | 19.07491 | 22.26755 | 1.082779273 | 118 | 17 | 0.686972 |
| PPS_0401 | General function prediction only | 4.750523 | 3.021025 | 2.858842 | 4.167 | 1.734082 | 5.566886 | 1.078791087 | 22 | 7 | 0.840136 |
| PPS_0444 | Transcription | 106.8868 | 111.7779 | 114.3537 | 103.1333 | 156.0674 | 99.09058 | 1.075890436 | 676 | 61 | 0.692196 |
| PPS_0246 | Energy production and conversion | 38.00418 | 42.29435 | 43.59734 | 39.5865 | 46.82022 | 46.76185 | 1.074842767 | 256 | 20 | 0.358136 |
| PPS_1483 | Nucleotide transport and metabolism | 0 | 3.021025 | 2.144131 | 2.0835 | 3.468165 | 0 | 1.07483005 | 11 | 3 | 0.92859 |
| PPS_2991 | General function prediction only | 2.375261 | 1.510512 | 2.144131 | 3.12525 | 0 | 3.340132 | 1.072219541 | 13 | 4 | 0.906808 |
| PPS_0023 | Translation | 32.06603 | 32.47601 | 27.87371 | 26.04375 | 32.94757 | 40.08158 | 1.072034788 | 189 | 22 | 0.648452 |
| PPS_1910 | Amino acid transport and metabolism | 3.562892 | 0 | 0 | 2.0835 | 1.734082 | 0 | 1.07148421 | 6 | 3 | 0.953751 |
| PPS_5234 | Energy production and conversion | 0 | 1.510512 | 1.429421 | 3.12525 | 0 | 0 | 1.063034368 | 7 | 3 | 0.960759 |
| PPS_0838 | Amino acid transport and metabolism | 0 | 1.510512 | 1.429421 | 3.12525 | 0 | 0 | 1.063034368 | 7 | 2 | 0.960759 |
| PPS_3675 |  | 53.44338 | 45.31537 | 33.59139 | 43.7535 | 58.9588 | 37.85483 | 1.062085246 | 262 | 25 | 0.764265 |
| PPS_0170 | General function prediction only | 1.187631 | 3.776281 | 5.002973 | 1.04175 | 1.734082 | 7.793641 | 1.060459104 | 22 | 9 | 0.939081 |
| PPS_4310 |  | 1.187631 | 3.021025 | 1.429421 | 3.12525 | 1.734082 | 1.113377 | 1.059352435 | 12 | 3 | 0.899272 |
| PPS_1473 | Function unknown | 2.375261 | 0 | 0.71471 | 1.04175 | 0 | 2.226755 | 1.057778155 | 6 | 3 | 0.953241 |
| PPS_0523 | Lipid metabolism | 2.375261 | 0 | 0.71471 | 1.04175 | 0 | 2.226755 | 1.057778155 | 6 | 2 | 0.953241 |
| PPS_0607 | Lipid metabolism | 2.375261 | 0 | 0.71471 | 1.04175 | 0 | 2.226755 | 1.057778155 | 6 | 2 | 0.953241 |
| PPS_0830 | Energy production and conversion | 2.375261 | 3.021025 | 5.717683 | 2.0835 | 5.202247 | 4.453509 | 1.056261356 | 23 | 3 | 0.888091 |
| PPS_1210 |  | 10.68868 | 8.307818 | 2.858842 | 8.334 | 6.93633 | 7.793641 | 1.055301616 | 43 | 10 | 0.878984 |
| PPS_4555 | Translation | 4.750523 | 3.776281 | 2.144131 | 2.0835 | 6.93633 | 2.226755 | 1.053945559 | 20 | 2 | 0.920649 |
| PPS_4361 | Cell envelope biogenesis | 10.68868 | 4.531537 | 10.00595 | 9.37575 | 10.40449 | 6.680264 | 1.048931329 | 50 | 13 | 0.865536 |
| PPS_4492 | Function unknown | 1.187631 | 0 | 2.858842 | 3.12525 | 0 | 1.113377 | 1.04748703 | 9 | 2 | 0.96113 |
| PPS_1127 | Function unknown | 1.187631 | 3.776281 | 5.002973 | 3.12525 | 1.734082 | 5.566886 | 1.046086052 | 22 | 3 | 0.927794 |
| PPS_2987 | Amino acid transport and metabolism | 16.62683 | 9.81833 | 9.291235 | 21.87675 | 12.13858 | 3.340132 | 1.045305751 | 71 | 12 | 0.932814 |
| PPS_4788 | Posttranslational modification | 5.938153 | 7.552562 | 5.717683 | 10.4175 | 5.202247 | 4.453509 | 1.045025 | 40 | 10 | 0.894771 |
| PPS_3359 | DNA replication | 5.938153 | 6.042049 | 9.291235 | 11.45925 | 5.202247 | 5.566886 | 1.044987349 | 45 | 8 | 0.898572 |
| PPS_3826 | Amino acid transport and metabolism | 10.68868 | 6.797305 | 13.5795 | 5.20875 | 13.87266 | 13.36053 | 1.044308258 | 62 | 10 | 0.900647 |
| PPS_4902 | Carbohydrate transport and metabolism | 11.87631 | 8.307818 | 7.861815 | 8.334 | 8.670412 | 12.24715 | 1.042987445 | 56 | 7 | 0.832787 |
| PPS_5150 | Transcription | 5.938153 | 8.307818 | 5.002973 | 10.4175 | 5.202247 | 4.453509 | 1.042823772 | 40 | 5 | 0.904928 |
| PPS_1113 | Translation | 19.00209 | 9.063074 | 17.15305 | 19.79325 | 17.34082 | 10.0204 | 1.042820251 | 90 | 3 | 0.886247 |
| PPS_0162 | Coenzyme metabolism | 4.750523 | 6.042049 | 2.144131 | 7.29225 | 1.734082 | 4.453509 | 1.041984298 | 27 | 9 | 0.931705 |
| PPS_5136 | Coenzyme metabolism | 8.313414 | 2.265768 | 4.288263 | 5.20875 | 6.93633 | 3.340132 | 1.041551589 | 28 | 6 | 0.92622 |
| PPS_3765 | Signal transduction mechanisms | 1.187631 | 0 | 2.144131 | 0 | 3.468165 | 0 | 1.04094017 | 6 | 2 | 0.974488 |
| PPS_1372 | Amino acid transport and metabolism | 1.187631 | 3.776281 | 5.717683 | 2.0835 | 3.468165 | 5.566886 | 1.040907405 | 23 | 7 | 0.934454 |
| PPS_3081 | Posttranslational modification | 39.19181 | 27.94448 | 30.73255 | 35.4195 | 32.94757 | 33.40132 | 1.039844621 | 196 | 10 | 0.740719 |
| PPS_0900 | Intracellular trafficking and secretion | 20.18972 | 53.62319 | 37.87965 | 16.668 | 38.14981 | 61.23575 | 1.039044696 | 234 | 5 | 0.932672 |
| PPS_2231 | Amino acid transport and metabolism | 8.313414 | 2.265768 | 5.002973 | 4.167 | 8.670412 | 3.340132 | 1.038209601 | 29 | 5 | 0.938293 |
| PPS_4354 | Cell envelope biogenesis | 2.375261 | 1.510512 | 2.144131 | 1.04175 | 5.202247 | 0 | 1.035505094 | 11 | 4 | 0.968493 |
| PPS_4512 | Cell envelope biogenesis | 2.375261 | 0 | 0.71471 | 2.0835 | 0 | 1.113377 | 1.034597596 | 6 | 2 | 0.971186 |
| PPS_0393 | Signal transduction mechanisms | 23.75261 | 9.063074 | 27.159 | 25.002 | 22.54307 | 14.4739 | 1.034085935 | 120 | 19 | 0.92154 |
| PPS_0476 | Translation | 22.56498 | 8.307818 | 10.72066 | 8.334 | 19.07491 | 15.58728 | 1.033724826 | 78 | 6 | 0.935844 |
| PPS_3634 | Secondary metabolites biosynthesis | 29.69077 | 30.9655 | 39.30907 | 32.29425 | 36.41573 | 34.5147 | 1.032604641 | 204 | 22 | 0.762692 |
| PPS_1937 | Signal transduction mechanisms | 1.187631 | 1.510512 | 0 | 1.04175 | 1.734082 | 0 | 1.028793672 | 5 | 4 | 0.971538 |
| PPS_0689 | General function prediction only | 8.313414 | 6.042049 | 10.72066 | 3.12525 | 10.40449 | 12.24715 | 1.027945883 | 50 | 9 | 0.944756 |
| PPS_0671 | General function prediction only | 19.00209 | 17.37089 | 17.15305 | 22.9185 | 17.34082 | 14.4739 | 1.022553448 | 108 | 17 | 0.88768 |
| PPS_0744 |  | 2.375261 | 0 | 1.429421 | 1.04175 | 1.734082 | 1.113377 | 1.022216722 | 7 | 2 | 0.971978 |
| PPS_4455 | Transcription | 2.375261 | 0.755256 | 0 | 2.0835 | 0 | 1.113377 | 1.021197726 | 6 | 2 | 0.982067 |
| PPS_4630 | Signal transduction mechanisms | 5.938153 | 1.510512 | 0.71471 | 8.334 | 0 | 0 | 1.02090117 | 16 | 6 | 0.986937 |
| PPS_0692 | Cell division and chromosome partitioning | 5.938153 | 6.042049 | 2.858842 | 8.334 | 3.468165 | 3.340132 | 1.020436119 | 30 | 12 | 0.961513 |
| PPS_4543 | Coenzyme metabolism | 2.375261 | 1.510512 | 1.429421 | 2.0835 | 0 | 3.340132 | 1.020401396 | 11 | 3 | 0.974481 |
| PPS_1687 | Coenzyme metabolism | 0 | 0.755256 | 1.429421 | 0 | 0 | 2.226755 | 1.0192603 | 5 | 2 | 0.987821 |
| PPS_3592 | Energy production and conversion | 16.62683 | 33.98653 | 15.00892 | 20.835 | 19.07491 | 26.72105 | 1.01537109 | 135 | 15 | 0.962496 |
| PPS_0981 | General function prediction only | 8.313414 | 6.797305 | 5.717683 | 6.2505 | 10.40449 | 4.453509 | 1.013447993 | 40 | 6 | 0.964509 |
| PPS_4169 | Nucleotide transport and metabolism | 0 | 3.021025 | 1.429421 | 1.04175 | 3.468165 | 0 | 1.01336255 | 9 | 2 | 0.988988 |
| PPS_0968 | Function unknown | 2.375261 | 2.265768 | 0.71471 | 2.0835 | 0 | 3.340132 | 1.012676426 | 11 | 3 | 0.984997 |
| PPS_1565 | Translation | 9.501045 | 13.59461 | 9.291235 | 14.5845 | 10.40449 | 7.793641 | 1.01221926 | 66 | 11 | 0.959474 |
| PPS_4922 | Intracellular trafficking and secretion | 1.187631 | 1.510512 | 2.858842 | 1.04175 | 3.468165 | 1.113377 | 1.011932266 | 11 | 3 | 0.982685 |
| PPS_4793 | General function prediction only | 3.562892 | 1.510512 | 3.573552 | 4.167 | 3.468165 | 1.113377 | 1.01174815 | 17 | 5 | 0.978042 |
| PPS_3547 | General function prediction only | 2.375261 | 0.755256 | 0.71471 | 1.04175 | 1.734082 | 1.113377 | 1.011438028 | 7 | 3 | 0.98192 |
| PPS_1341 | Lipid metabolism | 2.375261 | 1.510512 | 1.429421 | 3.12525 | 0 | 2.226755 | 1.00692545 | 11 | 2 | 0.990941 |
| PPS_1202 | Lipid metabolism | 13.06394 | 16.61564 | 11.43537 | 14.5845 | 15.60674 | 11.13377 | 1.005109457 | 82 | 9 | 0.974311 |
| PPS_4568 | Transcription | 7.125784 | 2.265768 | 2.858842 | 8.334 | 1.734082 | 2.226755 | 1.003627882 | 24 | 4 | 0.99578 |
| PPS_4852 |  | 4.750523 | 3.021025 | 4.288263 | 4.167 | 3.468165 | 4.453509 | 1.002393419 | 24 | 3 | 0.988046 |
| PPS_5155 | Intracellular trafficking and secretion | 5.938153 | 0.755256 | 1.429421 | 4.167 | 1.734082 | 2.226755 | 1.000616385 | 15 | 2 | 0.999318 |
| PPS_4174 | Posttranslational modification | 13.06394 | 8.307818 | 10.00595 | 6.2505 | 17.34082 | 7.793641 | 1.00023151 | 59 | 5 | 0.999529 |
| PPS_5064 | Energy production and conversion | 2.375261 | 0.755256 | 0 | 3.12525 | 0 | 0 | 0.998317396 | 6 | 2 | 0.99896 |
| PPS_4860 | Intracellular trafficking and secretion | 2.375261 | 0.755256 | 0 | 3.12525 | 0 | 0 | 0.998317396 | 6 | 2 | 0.99896 |
| PPS_3800 | Cell envelope biogenesis | 4.750523 | 1.510512 | 2.858842 | 6.2505 | 1.734082 | 1.113377 | 0.997596803 | 18 | 7 | 0.997116 |
| PPS_1413 | Translation | 147.2662 | 266.6054 | 228.7073 | 157.3043 | 209.824 | 272.7774 | 0.995839722 | 1314 | 26 | 0.986228 |
| PPS_0963 | Cell division and chromosome partitioning | 17.81446 | 20.39192 | 15.00892 | 12.501 | 22.54307 | 17.81404 | 0.993287885 | 104 | 13 | 0.973369 |
| PPS_0512 | Coenzyme metabolism | 1.187631 | 2.265768 | 2.144131 | 2.0835 | 3.468165 | 0 | 0.991806103 | 11 | 3 | 0.989622 |
| PPS_4463 | Function unknown | 8.313414 | 17.37089 | 12.15008 | 12.501 | 13.87266 | 11.13377 | 0.991358352 | 77 | 15 | 0.971402 |
| PPS_1201 |  | 0 | 3.021025 | 3.573552 | 2.0835 | 0 | 4.453509 | 0.991270452 | 15 | 8 | 0.991543 |
| PPS_1697 | Signal transduction mechanisms | 1.187631 | 4.531537 | 1.429421 | 3.12525 | 1.734082 | 2.226755 | 0.991256804 | 15 | 2 | 0.986874 |
|  |  | 7.125784 | 4.531537 | 5.717683 | 5.20875 | 8.670412 | 3.340132 | 0.991038257 | 33 | 6 | 0.978053 |
| PPS_4619 | Nucleotide transport and metabolism | 3.562892 | 4.531537 | 6.432394 | 10.4175 | 1.734082 | 2.226755 | 0.989778513 | 31 | 7 | 0.987886 |
| PPS_4934 | Translation | 15.4392 | 21.14717 | 20.01189 | 20.835 | 17.34082 | 17.81404 | 0.989250514 | 115 | 16 | 0.927136 |
| PPS_4348 | Intracellular trafficking and secretion | 32.06603 | 32.47601 | 27.87371 | 30.21075 | 24.27715 | 36.74145 | 0.987162416 | 185 | 21 | 0.926188 |
| PPS_0770 | Translation | 20.18972 | 20.39192 | 20.7266 | 16.668 | 26.01124 | 17.81404 | 0.986707055 | 120 | 11 | 0.934898 |
| PPS_1434 | General function prediction only | 0 | 0.755256 | 1.429421 | 1.04175 | 0 | 1.113377 | 0.986474094 | 5 | 5 | 0.986526 |
| PPS_0527 | General function prediction only | 0 | 0.755256 | 1.429421 | 1.04175 | 0 | 1.113377 | 0.986474094 | 5 | 3 | 0.986526 |
| PPS_4010 | Nucleotide transport and metabolism | 0 | 0.755256 | 1.429421 | 1.04175 | 0 | 1.113377 | 0.986474094 | 5 | 2 | 0.986526 |
| PPS_3818 | Translation | 21.37735 | 25.67871 | 30.73255 | 25.002 | 26.01124 | 25.60768 | 0.984988858 | 157 | 24 | 0.899037 |
| PPS_4653 | Amino acid transport and metabolism | 17.81446 | 12.83935 | 12.86479 | 10.4175 | 19.07491 | 13.36053 | 0.984703824 | 83 | 11 | 0.945724 |
| PPS_1457 | Nucleotide transport and metabolism | 13.06394 | 6.042049 | 8.576525 | 7.29225 | 12.13858 | 7.793641 | 0.983453683 | 52 | 8 | 0.955613 |
| PPS_4645 | Cell envelope biogenesis | 7.125784 | 7.552562 | 3.573552 | 7.29225 | 1.734082 | 8.907018 | 0.982547188 | 37 | 7 | 0.968777 |
| PPS_5088 | Amino acid transport and metabolism | 15.4392 | 20.39192 | 14.29421 | 14.5845 | 19.07491 | 15.58728 | 0.982471238 | 99 | 6 | 0.905925 |
| PPS_3975 | Carbohydrate transport and metabolism | 51.06812 | 46.07063 | 52.17386 | 44.79525 | 60.69288 | 41.19496 | 0.982389224 | 292 | 25 | 0.899861 |
| PPS_0978 | Carbohydrate transport and metabolism | 4.750523 | 2.265768 | 1.429421 | 2.0835 | 1.734082 | 4.453509 | 0.979324376 | 16 | 5 | 0.966816 |
| PPS_0908 | Amino acid transport and metabolism | 15.4392 | 33.98653 | 35.73552 | 15.62625 | 24.27715 | 43.42171 | 0.978439378 | 176 | 14 | 0.956327 |
| PPS_1417 | Cell envelope biogenesis | 11.87631 | 9.063074 | 9.291235 | 14.5845 | 10.40449 | 4.453509 | 0.973929996 | 59 | 8 | 0.938588 |
| PPS_4804 | Carbohydrate transport and metabolism | 27.3155 | 13.59461 | 26.44429 | 27.0855 | 17.34082 | 21.15417 | 0.973663059 | 133 | 13 | 0.916876 |
| PPS_4155 | Nucleotide transport and metabolism | 2.375261 | 2.265768 | 2.144131 | 3.12525 | 3.468165 | 0 | 0.971740358 | 13 | 4 | 0.959113 |
| PPS_0878 | Amino acid transport and metabolism | 5.938153 | 5.286793 | 3.573552 | 5.20875 | 6.93633 | 2.226755 | 0.971168408 | 28 | 2 | 0.932521 |
| PPS_5068 | Transcription | 32.06603 | 47.58114 | 39.30907 | 37.503 | 43.35206 | 34.5147 | 0.969850399 | 237 | 24 | 0.831409 |
| PPS_1204 | Nucleotide transport and metabolism | 16.62683 | 14.34987 | 15.00892 | 22.9185 | 13.87266 | 7.793641 | 0.969537981 | 91 | 14 | 0.925552 |
| PPS_0177 | General function prediction only | 1.187631 | 0.755256 | 1.429421 | 1.04175 | 0 | 2.226755 | 0.969218972 | 7 | 4 | 0.962995 |
| PPS_0352 | Energy production and conversion | 13.06394 | 3.776281 | 2.858842 | 10.4175 | 8.670412 | 0 | 0.968975806 | 35 | 7 | 0.966673 |
| PPS_0919 | Lipid metabolism | 2.375261 | 7.552562 | 7.147104 | 4.167 | 3.468165 | 8.907018 | 0.968799624 | 36 | 10 | 0.944193 |
| PPS_4319 | Signal transduction mechanisms | 0 | 1.510512 | 1.429421 | 0 | 1.734082 | 1.113377 | 0.968545713 | 6 | 3 | 0.967254 |
| PPS_0339 |  | 0 | 1.510512 | 1.429421 | 0 | 1.734082 | 1.113377 | 0.968545713 | 6 | 4 | 0.967254 |
| PPS_1281 | DNA replication | 30.8784 | 16.61564 | 25.72958 | 20.835 | 27.74532 | 22.26755 | 0.967554953 | 140 | 14 | 0.876317 |
| PPS_4893 | Amino acid transport and metabolism | 41.56707 | 63.44152 | 60.03568 | 48.96225 | 41.61798 | 69.02939 | 0.96707158 | 336 | 20 | 0.873383 |
| PPS_4267 | Cell envelope biogenesis | 45.12996 | 33.98653 | 35.02081 | 41.67 | 36.41573 | 32.28794 | 0.96702541 | 222 | 5 | 0.793929 |
| PPS_4350 | Cell envelope biogenesis | 7.125784 | 10.57359 | 4.288263 | 9.37575 | 5.202247 | 6.680264 | 0.96682809 | 44 | 7 | 0.917662 |
| PPS_0901 | Intracellular trafficking and secretion | 9.501045 | 10.57359 | 14.29421 | 13.54275 | 5.202247 | 14.4739 | 0.966541261 | 71 | 13 | 0.914703 |
| PPS_3879 | DNA replication | 3.562892 | 6.797305 | 0.71471 | 6.2505 | 0 | 4.453509 | 0.966509998 | 23 | 7 | 0.963764 |
| PPS_4035 | Nucleotide transport and metabolism | 34.44129 | 35.49704 | 36.45023 | 36.46125 | 29.4794 | 36.74145 | 0.965161116 | 212 | 26 | 0.658788 |
| PPS_0443 | Transcription | 143.7033 | 166.1564 | 130.792 | 145.845 | 142.1948 | 136.9454 | 0.964446956 | 869 | 57 | 0.667603 |
| PPS_3011 | Inorganic ion transport and metabolism | 5.938153 | 1.510512 | 2.858842 | 3.12525 | 3.468165 | 3.340132 | 0.963719593 | 19 | 3 | 0.932988 |
| PPS_0840 | Coenzyme metabolism | 3.562892 | 1.510512 | 4.288263 | 2.0835 | 6.93633 | 0 | 0.963485438 | 17 | 5 | 0.962645 |
| PPS_0829 | Cell envelope biogenesis | 7.125784 | 4.531537 | 3.573552 | 8.334 | 5.202247 | 1.113377 | 0.961837486 | 29 | 5 | 0.939395 |
| PPS_1575 | Amino acid transport and metabolism | 2.375261 | 11.32884 | 8.576525 | 7.29225 | 5.202247 | 8.907018 | 0.9605436 | 47 | 9 | 0.925585 |
| PPS_1541 | Posttranslational modification | 1.187631 | 1.510512 | 0.71471 | 1.04175 | 0 | 2.226755 | 0.957704355 | 7 | 4 | 0.949104 |
| PPS_1935 | Signal transduction mechanisms | 0 | 2.265768 | 0.71471 | 0 | 1.734082 | 1.113377 | 0.955369843 | 6 | 2 | 0.960579 |
| PPS_1800 | Function unknown | 4.750523 | 1.510512 | 4.288263 | 1.04175 | 3.468165 | 5.566886 | 0.955210645 | 20 | 3 | 0.928957 |
| PPS_1906 | Amino acid transport and metabolism | 15.4392 | 23.41294 | 17.15305 | 14.5845 | 12.13858 | 26.72105 | 0.954271058 | 113 | 9 | 0.877939 |
| PPS_0449 | Translation | 15.4392 | 17.37089 | 18.58247 | 17.70975 | 19.07491 | 12.24715 | 0.954064275 | 101 | 5 | 0.754529 |
| PPS_1157 | Nucleotide transport and metabolism | 29.69077 | 49.84691 | 45.02676 | 23.96025 | 31.21348 | 63.4625 | 0.952408633 | 252 | 8 | 0.893671 |
| PPS_4204 | Carbohydrate transport and metabolism | 3.562892 | 0 | 2.858842 | 1.04175 | 1.734082 | 3.340132 | 0.95238523 | 12 | 6 | 0.941261 |
| PPS_4527 | Function unknown | 15.4392 | 8.307818 | 10.72066 | 9.37575 | 15.60674 | 7.793641 | 0.950923874 | 64 | 10 | 0.867764 |
| PPS_3676 | Function unknown | 32.06603 | 30.21025 | 33.59139 | 34.37775 | 27.74532 | 28.94781 | 0.949964506 | 189 | 14 | 0.532721 |
| PPS_0568 | General function prediction only | 24.94024 | 15.86038 | 14.29421 | 21.87675 | 10.40449 | 20.04079 | 0.949672306 | 107 | 32 | 0.858625 |
| PPS_1418 | Carbohydrate transport and metabolism | 5.938153 | 3.021025 | 1.429421 | 4.167 | 3.468165 | 2.226755 | 0.94930218 | 19 | 5 | 0.911233 |
| PPS_0543 | Cell envelope biogenesis | 4.750523 | 3.776281 | 10.00595 | 7.29225 | 6.93633 | 3.340132 | 0.94798193 | 37 | 7 | 0.897117 |
| PPS_2569 |  | 30.8784 | 40.02858 | 48.60031 | 35.4195 | 27.74532 | 50.10198 | 0.947781541 | 242 | 17 | 0.815546 |
| PPS_1505 | DNA replication | 7.125784 | 7.552562 | 14.29421 | 11.45925 | 10.40449 | 5.566886 | 0.946779869 | 58 | 13 | 0.870457 |
| PPS_5152 | Translation | 22.56498 | 31.72076 | 35.73552 | 17.70975 | 32.94757 | 34.5147 | 0.94613216 | 178 | 8 | 0.82037 |
| PPS_2033 | Translation | 20.18972 | 55.1337 | 53.60328 | 20.835 | 36.41573 | 64.57588 | 0.94492925 | 264 | 8 | 0.896942 |
| PPS_0603 | Translation | 16.62683 | 16.61564 | 23.58544 | 21.87675 | 17.34082 | 14.4739 | 0.944808283 | 113 | 18 | 0.758002 |
| PPS_5272 | Energy production and conversion | 8.313414 | 9.81833 | 6.432394 | 8.334 | 10.40449 | 4.453509 | 0.944140729 | 47 | 3 | 0.833202 |
| PPS_1420 | Carbohydrate transport and metabolism | 3.562892 | 14.34987 | 10.72066 | 8.334 | 8.670412 | 10.0204 | 0.943820607 | 59 | 14 | 0.882034 |
| PPS_5085 | General function prediction only | 4.750523 | 4.531537 | 5.002973 | 7.29225 | 1.734082 | 4.453509 | 0.943633942 | 29 | 7 | 0.882767 |
| PPS_1079 | Secondary metabolites biosynthesis | 4.750523 | 3.021025 | 1.429421 | 5.20875 | 3.468165 | 0 | 0.943043687 | 17 | 6 | 0.928452 |
| PPS_3605 | Energy production and conversion | 9.501045 | 4.531537 | 5.717683 | 8.334 | 6.93633 | 3.340132 | 0.942289181 | 37 | 12 | 0.865943 |
| PPS_1669 | Function unknown | 7.125784 | 3.776281 | 6.432394 | 7.29225 | 3.468165 | 5.566886 | 0.941898546 | 34 | 3 | 0.834455 |
| PPS_1003 | DNA replication | 32.06603 | 41.53909 | 36.45023 | 27.0855 | 32.94757 | 43.42171 | 0.94002501 | 217 | 8 | 0.71478 |
| PPS_4754 | Function unknown | 4.750523 | 3.776281 | 4.288263 | 5.20875 | 3.468165 | 3.340132 | 0.937728041 | 25 | 6 | 0.717436 |
| PPS_1719 | Energy production and conversion | 3.562892 | 8.307818 | 6.432394 | 4.167 | 5.202247 | 7.793641 | 0.937703713 | 37 | 6 | 0.839395 |
| PPS_4678 | Posttranslational modification | 5.938153 | 1.510512 | 7.147104 | 6.2505 | 5.202247 | 2.226755 | 0.937223731 | 28 | 7 | 0.891887 |
| PPS_1716 | Carbohydrate transport and metabolism | 36.81655 | 49.09165 | 34.3061 | 47.9205 | 34.68165 | 30.06119 | 0.937187456 | 237 | 11 | 0.739056 |
| PPS_3516 | Energy production and conversion | 1.187631 | 1.510512 | 0.71471 | 2.0835 | 0 | 1.113377 | 0.936716852 | 7 | 2 | 0.919209 |
| PPS_1718 | Energy production and conversion | 8.313414 | 17.37089 | 8.576525 | 15.62625 | 8.670412 | 7.793641 | 0.936646945 | 69 | 9 | 0.861174 |
| PPS_3235 |  | 7.125784 | 3.776281 | 2.144131 | 4.167 | 6.93633 | 1.113377 | 0.936419091 | 23 | 3 | 0.907455 |
| PPS_3426 | Nucleotide transport and metabolism | 16.62683 | 15.86038 | 10.00595 | 10.4175 | 10.40449 | 18.92741 | 0.935430867 | 82 | 10 | 0.809195 |
| PPS_1041 | Energy production and conversion | 21.37735 | 13.59461 | 24.30015 | 19.79325 | 15.60674 | 20.04079 | 0.935360261 | 116 | 14 | 0.741436 |
| PPS_0772 | Translation | 27.3155 | 57.39947 | 63.60923 | 32.29425 | 26.01124 | 80.16316 | 0.933553992 | 306 | 10 | 0.881302 |
| PPS_1026 | Amino acid transport and metabolism | 9.501045 | 9.81833 | 6.432394 | 5.20875 | 12.13858 | 6.680264 | 0.93304621 | 48 | 4 | 0.823995 |
| PPS_3083 | Amino acid transport and metabolism | 11.87631 | 8.307818 | 11.43537 | 12.501 | 6.93633 | 10.0204 | 0.931631859 | 62 | 11 | 0.734201 |
| PPS_3590 | Energy production and conversion | 30.8784 | 24.1682 | 36.45023 | 23.96025 | 31.21348 | 30.06119 | 0.931561498 | 177 | 32 | 0.650006 |
| PPS_4636 | Translation | 15.4392 | 21.14717 | 16.43834 | 25.002 | 12.13858 | 12.24715 | 0.931409646 | 106 | 20 | 0.811921 |
| PPS_0026 | Cell envelope biogenesis | 0 | 2.265768 | 0.71471 | 1.04175 | 1.734082 | 0 | 0.931337708 | 6 | 2 | 0.939269 |
| PPS_5034 | Amino acid transport and metabolism | 32.06603 | 27.18922 | 20.7266 | 31.2525 | 24.27715 | 18.92741 | 0.930924531 | 153 | 13 | 0.723557 |
| PPS_1885 | DNA replication | 74.82073 | 88.36497 | 91.48293 | 55.21275 | 79.76779 | 101.3173 | 0.927864054 | 498 | 6 | 0.701064 |
| PPS_1439 | Defense mechanisms | 2.375261 | 6.042049 | 3.573552 | 2.0835 | 3.468165 | 5.566886 | 0.92725198 | 24 | 8 | 0.853867 |
| PPS_0447 | Translation | 157.9549 | 239.4162 | 203.6925 | 155.2208 | 199.4195 | 202.6347 | 0.927148038 | 1181 | 45 | 0.635172 |
| PPS_0988 | Secondary metabolites biosynthesis | 5.938153 | 3.021025 | 2.144131 | 5.20875 | 1.734082 | 3.340132 | 0.926117084 | 21 | 3 | 0.866487 |
| PPS_3273 | Coenzyme metabolism | 1.187631 | 1.510512 | 1.429421 | 2.0835 | 1.734082 | 0 | 0.92489967 | 8 | 2 | 0.887985 |
| PPS_4289 |  | 9.501045 | 15.86038 | 15.00892 | 9.37575 | 12.13858 | 15.58728 | 0.919031287 | 80 | 4 | 0.705705 |
| PPS_1921 | Energy production and conversion | 49.88049 | 61.93101 | 70.75633 | 52.0875 | 55.49064 | 60.12237 | 0.918565529 | 359 | 37 | 0.508393 |
| PPS_3989 | Cell motility and secretion | 3.562892 | 5.286793 | 1.429421 | 3.12525 | 5.202247 | 1.113377 | 0.918452887 | 19 | 2 | 0.871791 |
| PPS_0012 | DNA replication | 27.3155 | 19.63666 | 21.44131 | 28.12725 | 19.07491 | 15.58728 | 0.918061789 | 131 | 17 | 0.696887 |
| PPS_1205 |  | 2.375261 | 3.021025 | 1.429421 | 6.2505 | 0 | 0 | 0.915729349 | 14 | 5 | 0.935944 |
| PPS_5154 | Cell envelope biogenesis | 1.187631 | 1.510512 | 0.71471 | 3.12525 | 0 | 0 | 0.915729349 | 7 | 3 | 0.935944 |
| PPS_0679 | Posttranslational modification | 14.25157 | 14.34987 | 10.72066 | 11.45925 | 12.13858 | 12.24715 | 0.911573517 | 75 | 16 | 0.435184 |
| PPS_4400 | Signal transduction mechanisms | 11.87631 | 7.552562 | 9.291235 | 7.29225 | 12.13858 | 6.680264 | 0.909157256 | 53 | 7 | 0.706491 |
| PPS_3302 | Energy production and conversion | 1.187631 | 2.265768 | 6.432394 | 1.04175 | 3.468165 | 4.453509 | 0.906697516 | 20 | 3 | 0.880226 |
| PPS_4807 | Carbohydrate transport and metabolism | 28.50314 | 21.90243 | 15.72363 | 22.9185 | 22.54307 | 14.4739 | 0.906339135 | 123 | 16 | 0.678864 |
| PPS_0433 | Coenzyme metabolism | 11.87631 | 9.063074 | 19.29718 | 11.45925 | 13.87266 | 11.13377 | 0.906282257 | 78 | 5 | 0.725492 |
| PPS_3771 | Cell motility and secretion | 0 | 0.755256 | 2.858842 | 1.04175 | 0 | 2.226755 | 0.904376327 | 8 | 6 | 0.919842 |
| PPS_0602 | Coenzyme metabolism | 4.750523 | 0 | 0.71471 | 2.0835 | 1.734082 | 1.113377 | 0.902241446 | 9 | 3 | 0.916001 |
| PPS_1312 |  | 2.375261 | 0.755256 | 3.573552 | 2.0835 | 1.734082 | 2.226755 | 0.901592221 | 13 | 4 | 0.814435 |
| PPS_1188 | Nucleotide transport and metabolism | 7.125784 | 6.042049 | 5.717683 | 6.2505 | 5.202247 | 5.566886 | 0.901200324 | 36 | 4 | 0.307718 |
| PPS_1578 | Amino acid transport and metabolism | 21.37735 | 10.57359 | 9.291235 | 15.62625 | 10.40449 | 11.13377 | 0.901128973 | 76 | 10 | 0.767837 |
| PPS_1829 | Lipid metabolism | 3.562892 | 6.797305 | 7.861815 | 6.2505 | 3.468165 | 6.680264 | 0.899951576 | 37 | 7 | 0.730583 |
| PPS_1171 | Lipid metabolism | 2.375261 | 0 | 0.71471 | 1.04175 | 1.734082 | 0 | 0.89833587 | 5 | 2 | 0.910101 |
| PPS_0982 | Function unknown | 2.375261 | 0 | 0.71471 | 1.04175 | 1.734082 | 0 | 0.89833587 | 5 | 2 | 0.910101 |
| PPS_3678 | DNA replication | 2.375261 | 6.042049 | 0.71471 | 3.12525 | 1.734082 | 3.340132 | 0.897880571 | 18 | 5 | 0.865488 |
| PPS_0453 | Translation | 26.12787 | 35.49704 | 31.44726 | 17.70975 | 34.68165 | 31.17456 | 0.897861946 | 178 | 13 | 0.624922 |
| PPS_1183 | Amino acid transport and metabolism | 7.125784 | 5.286793 | 2.858842 | 6.2505 | 5.202247 | 2.226755 | 0.895758416 | 28 | 8 | 0.773867 |
| PPS_2635 | Posttranslational modification | 9.501045 | 9.81833 | 12.86479 | 14.5845 | 8.670412 | 5.566886 | 0.895527361 | 63 | 4 | 0.724061 |
| PPS_5069 | Posttranslational modification | 48.69286 | 62.68626 | 52.17386 | 60.4215 | 46.82022 | 38.9682 | 0.893960666 | 317 | 6 | 0.492093 |
| PPS_3658 | Function unknown | 2.375261 | 0.755256 | 2.858842 | 3.12525 | 0 | 2.226755 | 0.893585515 | 12 | 2 | 0.860648 |
| PPS_4340 | Cell envelope biogenesis | 5.938153 | 5.286793 | 5.717683 | 3.12525 | 8.670412 | 3.340132 | 0.893355642 | 31 | 4 | 0.771989 |
| PPS_4011 |  | 9.501045 | 22.65768 | 24.30015 | 3.12525 | 29.4794 | 17.81404 | 0.893016002 | 108 | 11 | 0.835116 |
| PPS_1577 | Amino acid transport and metabolism | 21.37735 | 17.37089 | 17.86776 | 17.70975 | 13.87266 | 18.92741 | 0.892147442 | 108 | 13 | 0.363343 |
| PPS_3422 | Energy production and conversion | 59.38153 | 78.54664 | 77.18873 | 66.672 | 57.22472 | 67.91601 | 0.891667435 | 420 | 25 | 0.34794 |
| PPS_4571 | Amino acid transport and metabolism | 5.938153 | 7.552562 | 5.002973 | 5.20875 | 3.468165 | 7.793641 | 0.890604185 | 36 | 6 | 0.673459 |
| PPS_3911 | Signal transduction mechanisms | 2.375261 | 0.755256 | 0 | 1.04175 | 1.734082 | 0 | 0.886700831 | 5 | 4 | 0.898295 |
| PPS_1793 | Transcription | 2.375261 | 0.755256 | 0 | 1.04175 | 1.734082 | 0 | 0.886700831 | 5 | 2 | 0.898295 |
| PPS_0474 | Translation | 11.87631 | 58.15472 | 35.73552 | 29.169 | 12.13858 | 52.32873 | 0.885311158 | 219 | 6 | 0.830939 |
|  |  | 21.37735 | 27.18922 | 21.44131 | 23.96025 | 19.07491 | 18.92741 | 0.885079863 | 135 | 26 | 0.351705 |
| PPS_1323 | Coenzyme metabolism | 3.562892 | 3.021025 | 1.429421 | 3.12525 | 1.734082 | 2.226755 | 0.884286609 | 15 | 3 | 0.708137 |
| PPS_0088 | DNA replication | 14.25157 | 8.307818 | 7.147104 | 10.4175 | 6.93633 | 8.907018 | 0.884010469 | 55 | 15 | 0.669588 |
| PPS_4550 | Cell envelope biogenesis | 1.187631 | 3.021025 | 4.288263 | 4.167 | 0 | 3.340132 | 0.883512349 | 18 | 9 | 0.843897 |
| PPS_4747 | Cell motility and secretion | 1.187631 | 3.021025 | 2.858842 | 1.04175 | 5.202247 | 0 | 0.883480703 | 13 | 5 | 0.88329 |
| PPS_4809 | Carbohydrate transport and metabolism | 11.87631 | 12.0841 | 11.43537 | 7.29225 | 13.87266 | 10.0204 | 0.881046045 | 66 | 17 | 0.539216 |
| PPS_0690 | Amino acid transport and metabolism | 3.562892 | 2.265768 | 4.288263 | 2.0835 | 3.468165 | 3.340132 | 0.878903268 | 19 | 5 | 0.611875 |
| PPS_0186 | Function unknown | 21.37735 | 18.8814 | 27.159 | 23.96025 | 20.80899 | 14.4739 | 0.878746948 | 129 | 14 | 0.504413 |
| PPS_0960 | Translation | 9.501045 | 15.86038 | 12.86479 | 10.4175 | 8.670412 | 14.4739 | 0.877979133 | 75 | 13 | 0.57014 |
| PPS_0183 |  | 3.562892 | 1.510512 | 2.858842 | 5.20875 | 1.734082 | 0 | 0.875266915 | 15 | 8 | 0.855753 |
| PPS_5141 | DNA replication | 8.313414 | 3.021025 | 2.858842 | 7.29225 | 1.734082 | 3.340132 | 0.871290045 | 26 | 3 | 0.815053 |
| PPS_0418 | Amino acid transport and metabolism | 9.501045 | 12.83935 | 7.147104 | 6.2505 | 13.87266 | 5.566886 | 0.871218043 | 54 | 9 | 0.710652 |
| PPS_1648 | Coenzyme metabolism | 11.87631 | 6.042049 | 5.717683 | 5.20875 | 8.670412 | 6.680264 | 0.869833801 | 42 | 6 | 0.678513 |
| PPS_1054 | Amino acid transport and metabolism | 20.18972 | 32.47601 | 28.58842 | 22.9185 | 24.27715 | 23.38092 | 0.868590392 | 157 | 18 | 0.429986 |
| PPS_3603 | Energy production and conversion | 10.68868 | 34.74178 | 37.87965 | 11.45925 | 17.34082 | 43.42171 | 0.866903023 | 168 | 9 | 0.791252 |
| PPS_1014 | Transcription | 4.750523 | 4.531537 | 7.147104 | 0 | 8.670412 | 5.566886 | 0.866586917 | 30 | 4 | 0.805986 |
| PPS_2998 | Coenzyme metabolism | 7.125784 | 6.797305 | 5.717683 | 6.2505 | 5.202247 | 5.566886 | 0.866546031 | 37 | 6 | 0.178201 |
| PPS_4921 | Amino acid transport and metabolism | 61.75679 | 46.82588 | 44.31205 | 44.79525 | 48.55431 | 38.9682 | 0.865417464 | 282 | 39 | 0.344279 |
| PPS_4460 | Function unknown | 4.750523 | 2.265768 | 2.144131 | 0 | 3.468165 | 4.453509 | 0.864771693 | 16 | 2 | 0.810803 |
| PPS_4471 | Signal transduction mechanisms | 9.501045 | 4.531537 | 4.288263 | 7.29225 | 5.202247 | 3.340132 | 0.864295801 | 33 | 7 | 0.709007 |
| PPS_1060 | Nucleotide transport and metabolism | 14.25157 | 15.86038 | 17.15305 | 12.501 | 13.87266 | 14.4739 | 0.864224399 | 90 | 14 | 0.11292 |
| PPS_3073 | Carbohydrate transport and metabolism | 11.87631 | 3.776281 | 7.861815 | 7.29225 | 5.202247 | 7.793641 | 0.862796269 | 43 | 10 | 0.698564 |
| PPS_0033 | Translation | 0 | 3.021025 | 2.144131 | 0 | 0 | 4.453509 | 0.862221617 | 11 | 3 | 0.899173 |
| PPS_1002 | General function prediction only | 1.187631 | 2.265768 | 4.288263 | 2.0835 | 3.468165 | 1.113377 | 0.860931718 | 15 | 8 | 0.769218 |
| PPS_4378 | Translation | 8.313414 | 11.32884 | 7.861815 | 7.29225 | 5.202247 | 11.13377 | 0.859082624 | 53 | 7 | 0.568782 |
| PPS_0915 | Nucleotide transport and metabolism | 22.56498 | 11.32884 | 18.58247 | 9.37575 | 15.60674 | 20.04079 | 0.857973722 | 96 | 7 | 0.61155 |
| PPS_4362 | Cell envelope biogenesis | 8.313414 | 3.021025 | 3.573552 | 8.334 | 0 | 4.453509 | 0.857762051 | 28 | 6 | 0.822903 |
| PPS_2575 | General function prediction only | 4.750523 | 10.57359 | 7.861815 | 2.0835 | 12.13858 | 5.566886 | 0.853490403 | 43 | 7 | 0.759488 |
| PPS_3602 | Amino acid transport and metabolism | 2.375261 | 2.265768 | 0 | 0 | 1.734082 | 2.226755 | 0.853439249 | 8 | 3 | 0.836317 |
| PPS_0537 | General function prediction only | 2.375261 | 2.265768 | 0 | 0 | 1.734082 | 2.226755 | 0.853439249 | 8 | 3 | 0.836317 |
| PPS_2065 | Posttranslational modification | 2.375261 | 0.755256 | 1.429421 | 1.04175 | 1.734082 | 1.113377 | 0.852908404 | 8 | 3 | 0.697107 |
| PPS_0245 | Posttranslational modification | 10.68868 | 1.510512 | 5.002973 | 6.2505 | 1.734082 | 6.680264 | 0.852500222 | 31 | 7 | 0.801889 |
| PPS_4892 | Coenzyme metabolism | 1.187631 | 0 | 1.429421 | 0 | 0 | 2.226755 | 0.850863869 | 5 | 2 | 0.889121 |
| PPS_0939 | Translation | 4.750523 | 5.286793 | 10.00595 | 6.2505 | 5.202247 | 5.566886 | 0.84914491 | 39 | 7 | 0.609553 |
| PPS_0415 | Lipid metabolism | 11.87631 | 6.042049 | 2.858842 | 6.2505 | 6.93633 | 4.453509 | 0.849023977 | 36 | 6 | 0.735208 |
| PPS_3747 | Cell motility and secretion | 2.375261 | 3.021025 | 2.144131 | 4.167 | 0 | 2.226755 | 0.847931141 | 15 | 5 | 0.783466 |
| PPS_4651 | Function unknown | 5.938153 | 1.510512 | 2.144131 | 4.167 | 1.734082 | 2.226755 | 0.847285433 | 17 | 3 | 0.77563 |
| PPS_0899 | Translation | 7.125784 | 2.265768 | 6.432394 | 3.12525 | 6.93633 | 3.340132 | 0.846926004 | 28 | 7 | 0.701947 |
| PPS_0481 | DNA replication | 7.125784 | 8.307818 | 7.861815 | 8.334 | 6.93633 | 4.453509 | 0.846683249 | 44 | 5 | 0.406828 |
| PPS_4609 | Function unknown | 4.750523 | 1.510512 | 2.858842 | 3.12525 | 3.468165 | 1.113377 | 0.84505443 | 16 | 2 | 0.714157 |
| PPS_3589 | Energy production and conversion | 27.3155 | 58.90998 | 43.59734 | 37.503 | 36.41573 | 35.62807 | 0.843817768 | 251 | 12 | 0.536095 |
| PPS_4141 | Cell envelope biogenesis | 2.375261 | 3.776281 | 3.573552 | 3.12525 | 1.734082 | 3.340132 | 0.843124401 | 19 | 3 | 0.48888 |
| PPS_5067 | Coenzyme metabolism | 5.938153 | 0 | 0 | 1.04175 | 1.734082 | 2.226755 | 0.842448286 | 9 | 4 | 0.890169 |
| PPS_4333 | Posttranslational modification | 15.4392 | 10.57359 | 12.86479 | 9.37575 | 12.13858 | 11.13377 | 0.839766934 | 71 | 6 | 0.285319 |
| PPS_3588 | Energy production and conversion | 60.56916 | 89.87548 | 73.61517 | 56.2545 | 53.75655 | 77.93641 | 0.838827168 | 428 | 21 | 0.352483 |
| PPS_5162 | DNA replication | 11.87631 | 8.307818 | 8.576525 | 9.37575 | 3.468165 | 11.13377 | 0.833697716 | 54 | 6 | 0.582325 |
| PPS_0807 |  | 1.187631 | 5.286793 | 2.858842 | 2.0835 | 3.468165 | 2.226755 | 0.833408133 | 18 | 4 | 0.714873 |
| PPS_4459 | Secondary metabolites biosynthesis | 3.562892 | 2.265768 | 1.429421 | 2.0835 | 1.734082 | 2.226755 | 0.832773395 | 13 | 2 | 0.584927 |
| PPS_1479 | Cell envelope biogenesis | 4.750523 | 6.042049 | 3.573552 | 6.2505 | 3.468165 | 2.226755 | 0.831499115 | 27 | 7 | 0.598363 |
| PPS_0189 |  | 22.56498 | 18.12615 | 15.72363 | 17.70975 | 19.07491 | 10.0204 | 0.829659694 | 102 | 21 | 0.41195 |
| PPS_3972 | General function prediction only | 3.562892 | 0.755256 | 2.144131 | 3.12525 | 0 | 2.226755 | 0.82819146 | 12 | 3 | 0.779242 |
| PPS_1547 | Secondary metabolites biosynthesis | 17.81446 | 8.307818 | 8.576525 | 9.37575 | 10.40449 | 8.907018 | 0.8267508 | 61 | 6 | 0.588245 |
| PPS_0190 |  | 14.25157 | 16.61564 | 16.43834 | 19.79325 | 10.40449 | 8.907018 | 0.826642296 | 90 | 11 | 0.509044 |
|  |  | 9.501045 | 8.307818 | 5.717683 | 5.20875 | 8.670412 | 5.566886 | 0.826557718 | 42 | 8 | 0.434333 |
| PPS_4821 | Amino acid transport and metabolism | 9.501045 | 4.531537 | 4.288263 | 3.12525 | 8.670412 | 3.340132 | 0.826151533 | 31 | 4 | 0.691266 |
| PPS_4791 | Energy production and conversion | 2.375261 | 3.776281 | 4.288263 | 4.167 | 0 | 4.453509 | 0.825734715 | 21 | 12 | 0.725022 |
| PPS_3595 | Energy production and conversion | 14.25157 | 21.14717 | 13.5795 | 17.70975 | 10.40449 | 12.24715 | 0.824067911 | 93 | 12 | 0.429116 |
| PPS_3997 | Intracellular trafficking and secretion | 1.187631 | 0 | 1.429421 | 1.04175 | 0 | 1.113377 | 0.823494414 | 5 | 3 | 0.800843 |
| PPS_4563 | Coenzyme metabolism | 2.375261 | 1.510512 | 0 | 2.0835 | 0 | 1.113377 | 0.822713214 | 7 | 2 | 0.815175 |
| PPS_1314 | Nucleotide transport and metabolism | 27.3155 | 21.14717 | 20.01189 | 19.79325 | 24.27715 | 12.24715 | 0.822459411 | 123 | 13 | 0.395682 |
| PPS_3427 | Function unknown | 0 | 3.776281 | 2.144131 | 3.12525 | 1.734082 | 0 | 0.820776043 | 12 | 3 | 0.815819 |
| PPS_1566 |  | 8.313414 | 3.776281 | 2.858842 | 2.0835 | 3.468165 | 6.680264 | 0.818269273 | 26 | 9 | 0.698428 |
| PPS_3255 | Inorganic ion transport and metabolism | 9.501045 | 8.307818 | 5.717683 | 6.2505 | 5.202247 | 7.793641 | 0.818071124 | 43 | 6 | 0.35685 |
| PPS_0261 |  | 39.19181 | 30.9655 | 30.73255 | 34.37775 | 22.54307 | 25.60768 | 0.818005864 | 186 | 15 | 0.249776 |
| PPS_0228 | Posttranslational modification | 2.375261 | 0.755256 | 2.144131 | 2.0835 | 0 | 2.226755 | 0.817164289 | 10 | 4 | 0.73527 |
| PPS_0362 | Coenzyme metabolism | 5.938153 | 1.510512 | 0.71471 | 2.0835 | 3.468165 | 1.113377 | 0.816456593 | 13 | 3 | 0.797322 |
| PPS_2533 | Energy production and conversion | 5.938153 | 2.265768 | 5.002973 | 5.20875 | 0 | 5.566886 | 0.815909924 | 25 | 8 | 0.724187 |
| PPS_1946 | Amino acid transport and metabolism | 2.375261 | 1.510512 | 2.858842 | 1.04175 | 0 | 4.453509 | 0.814762423 | 13 | 4 | 0.790698 |
| PPS_4929 | Cell envelope biogenesis | 0 | 3.776281 | 1.429421 | 3.12525 | 0 | 1.113377 | 0.814227851 | 11 | 5 | 0.833177 |
| PPS_3518 | Energy production and conversion | 8.313414 | 9.063074 | 8.576525 | 9.37575 | 1.734082 | 10.0204 | 0.814172424 | 50 | 13 | 0.607585 |
| PPS_5266 |  | 1.187631 | 0.755256 | 0.71471 | 1.04175 | 0 | 1.113377 | 0.810930738 | 5 | 4 | 0.699893 |
| PPS_1153 | Cell envelope biogenesis | 1.187631 | 0.755256 | 0.71471 | 1.04175 | 0 | 1.113377 | 0.810930738 | 5 | 3 | 0.699893 |
| PPS_4692 |  | 2.375261 | 1.510512 | 1.429421 | 2.0835 | 0 | 2.226755 | 0.810930738 | 10 | 4 | 0.699893 |
| PPS_4884 | Function unknown | 2.375261 | 3.021025 | 0.71471 | 2.0835 | 1.734082 | 1.113377 | 0.806899465 | 11 | 3 | 0.637526 |
| PPS_1394 | Function unknown | 10.68868 | 4.531537 | 5.717683 | 8.334 | 5.202247 | 3.340132 | 0.806020767 | 37 | 11 | 0.602191 |
| PPS_4920 | Amino acid transport and metabolism | 15.4392 | 23.41294 | 15.00892 | 16.668 | 15.60674 | 11.13377 | 0.805935045 | 100 | 14 | 0.35053 |
| PPS_4007 |  | 7.125784 | 3.021025 | 3.573552 | 3.12525 | 3.468165 | 4.453509 | 0.805148221 | 24 | 6 | 0.566389 |
| PPS_0894 | Amino acid transport and metabolism | 1.187631 | 2.265768 | 5.002973 | 0 | 3.468165 | 3.340132 | 0.805108449 | 16 | 4 | 0.749483 |
| PPS_0439 | Translation | 11.87631 | 12.83935 | 7.147104 | 10.4175 | 5.202247 | 10.0204 | 0.804705505 | 59 | 7 | 0.441498 |
| PPS_1604 | Amino acid transport and metabolism | 24.94024 | 21.14717 | 22.87073 | 21.87675 | 19.07491 | 14.4739 | 0.803756503 | 126 | 20 | 0.16025 |
| PPS_1549 | Lipid metabolism | 7.125784 | 2.265768 | 5.002973 | 5.20875 | 5.202247 | 1.113377 | 0.800608168 | 25 | 4 | 0.650945 |
| PPS_3410 | Translation | 22.56498 | 11.32884 | 17.15305 | 17.70975 | 5.202247 | 17.81404 | 0.797816397 | 94 | 11 | 0.553481 |
| PPS_3995 | Intracellular trafficking and secretion | 8.313414 | 3.776281 | 4.288263 | 6.2505 | 3.468165 | 3.340132 | 0.797339742 | 29 | 10 | 0.560169 |
| PPS_0803 | Nucleotide transport and metabolism | 5.938153 | 2.265768 | 2.858842 | 3.12525 | 3.468165 | 2.226755 | 0.797284465 | 19 | 4 | 0.586132 |
| PPS_1375 | Cell division and chromosome partitioning | 14.25157 | 14.34987 | 16.43834 | 12.501 | 15.60674 | 7.793641 | 0.79710396 | 82 | 14 | 0.310767 |
| PPS_1544 | Translation | 7.125784 | 3.021025 | 9.291235 | 5.20875 | 6.93633 | 3.340132 | 0.796644532 | 35 | 2 | 0.574827 |
| PPS_1979 | Function unknown | 1.187631 | 0 | 1.429421 | 2.0835 | 0 | 0 | 0.79612496 | 5 | 2 | 0.841285 |
| PPS_4496 | Lipid metabolism | 5.938153 | 0.755256 | 1.429421 | 3.12525 | 0 | 3.340132 | 0.795951866 | 14 | 5 | 0.793238 |
| PPS_4998 | Carbohydrate transport and metabolism | 4.750523 | 1.510512 | 2.144131 | 2.0835 | 3.468165 | 1.113377 | 0.792969702 | 14 | 3 | 0.658162 |
| PPS_4901 | Inorganic ion transport and metabolism | 3.562892 | 2.265768 | 2.144131 | 5.20875 | 0 | 1.113377 | 0.792962811 | 15 | 3 | 0.766142 |
| PPS_0092 | Posttranslational modification | 10.68868 | 12.0841 | 14.29421 | 8.334 | 12.13858 | 8.907018 | 0.792608214 | 68 | 6 | 0.181731 |
| PPS_4843 | Nucleotide transport and metabolism | 11.87631 | 12.83935 | 10.00595 | 10.4175 | 10.40449 | 6.680264 | 0.792079066 | 63 | 6 | 0.193247 |
| PPS_4765 | Coenzyme metabolism | 28.50314 | 21.14717 | 17.15305 | 20.835 | 20.80899 | 11.13377 | 0.790046537 | 118 | 12 | 0.37019 |
| PPS_0454 | Translation | 2.375261 | 3.021025 | 3.573552 | 3.12525 | 1.734082 | 2.226755 | 0.789990513 | 17 | 3 | 0.30685 |
| PPS_1410 | Amino acid transport and metabolis | 5.938153 | 6.797305 | 8.576525 | 2.0835 | 6.93633 | 7.793641 | 0.788920954 | 39 | 9 | 0.500803 |
| PPS_0413 | Energy production and conversion | 57.00627 | 37.76281 | 53.60328 | 45.837 | 39.8839 | 31.17456 | 0.787851988 | 268 | 26 | 0.230896 |
| PPS_4948 | General function prediction only | 1.187631 | 2.265768 | 0.71471 | 1.04175 | 0 | 2.226755 | 0.784169541 | 8 | 3 | 0.725543 |
| PPS_4880 | Transcription | 1.187631 | 0.755256 | 0.71471 | 2.0835 | 0 | 0 | 0.783978847 | 5 | 2 | 0.811031 |
| PPS_0906 | Amino acid transport and metabolism | 1.187631 | 0.755256 | 0.71471 | 2.0835 | 0 | 0 | 0.783978847 | 5 | 2 | 0.811031 |
| PPS_3884 |  | 1.187631 | 2.265768 | 2.144131 | 1.04175 | 0 | 3.340132 | 0.782824123 | 11 | 4 | 0.728856 |
| PPS_4372 | Cytoplasmic | 1.187631 | 2.265768 | 1.429421 | 2.0835 | 1.734082 | 0 | 0.78183968 | 9 | 2 | 0.657091 |
| PPS_0821 |  | 13.06394 | 3.021025 | 9.291235 | 7.29225 | 6.93633 | 5.566886 | 0.780080084 | 44 | 11 | 0.59229 |
| PPS_4923 | Amino acid transport and metabolism | 3.562892 | 9.063074 | 5.717683 | 6.2505 | 6.93633 | 1.113377 | 0.77957263 | 34 | 6 | 0.610122 |
| PPS_0843 | Defense mechanisms | 0 | 1.510512 | 2.144131 | 0 | 1.734082 | 1.113377 | 0.779134708 | 7 | 3 | 0.758216 |
| PPS_5139 | Amino acid transport and metabolism | 14.25157 | 8.307818 | 13.5795 | 6.2505 | 5.202247 | 16.70066 | 0.779033651 | 66 | 12 | 0.564845 |
| PPS_4156 | Transcription | 2.375261 | 5.286793 | 6.432394 | 4.167 | 3.468165 | 3.340132 | 0.778696438 | 27 | 4 | 0.481952 |
| PPS_4854 | Coenzyme metabolism | 2.375261 | 10.57359 | 8.576525 | 8.334 | 1.734082 | 6.680264 | 0.778074616 | 43 | 8 | 0.642582 |
| PPS_4758 | DNA replication | 1.187631 | 4.531537 | 0.71471 | 1.04175 | 1.734082 | 2.226755 | 0.777538355 | 12 | 7 | 0.7347 |
| PPS_0383 | Transcription | 19.00209 | 21.14717 | 23.58544 | 17.70975 | 13.87266 | 17.81404 | 0.775032132 | 118 | 15 | 0.061423 |
| PPS_3748 | Cell motility and secretion | 8.313414 | 6.797305 | 5.717683 | 3.12525 | 5.202247 | 7.793641 | 0.77399779 | 37 | 9 | 0.381991 |
| PPS_4810 | Coenzyme metabolism | 1.187631 | 1.510512 | 0 | 2.0835 | 0 | 0 | 0.77219778 | 5 | 3 | 0.819488 |
| PPS_0800 | General function prediction only | 1.187631 | 1.510512 | 0 | 2.0835 | 0 | 0 | 0.77219778 | 5 | 2 | 0.819488 |
| PPS_3982 | Posttranslational modification | 2.375261 | 1.510512 | 2.858842 | 0 | 5.202247 | 0 | 0.771318597 | 11 | 3 | 0.797438 |
| PPS_1539 | Translation | 1.187631 | 3.776281 | 1.429421 | 2.0835 | 1.734082 | 1.113377 | 0.771265976 | 12 | 5 | 0.623172 |
| PPS_1588 | Amino acid transport and metabolism | 4.750523 | 5.286793 | 5.002973 | 4.167 | 5.202247 | 2.226755 | 0.770995959 | 27 | 4 | 0.317742 |
| PPS_1189 | Translation | 11.87631 | 4.531537 | 4.288263 | 6.2505 | 5.202247 | 4.453509 | 0.768562764 | 35 | 5 | 0.589775 |
| PPS_1663 | Function unknown | 4.750523 | 8.307818 | 4.288263 | 4.167 | 6.93633 | 2.226755 | 0.768455026 | 31 | 4 | 0.512824 |
| PPS_1308 | Signal transduction mechanisms | 4.750523 | 9.063074 | 5.002973 | 4.167 | 6.93633 | 3.340132 | 0.767592704 | 34 | 13 | 0.459205 |
| PPS_0384 | DNA replication | 3.562892 | 2.265768 | 2.144131 | 1.04175 | 1.734082 | 3.340132 | 0.767104483 | 14 | 4 | 0.497277 |
| PPS_4632 | Inorganic ion transport and metabolism | 5.938153 | 1.510512 | 1.429421 | 0 | 3.468165 | 3.340132 | 0.766865332 | 14 | 4 | 0.732503 |
| PPS_0992 | Cell envelope biogenesis | 11.87631 | 12.83935 | 17.86776 | 4.167 | 17.34082 | 11.13377 | 0.766532971 | 76 | 13 | 0.492685 |
| PPS_4633 | Cell envelope biogenesis | 2.375261 | 0.755256 | 2.858842 | 0 | 3.468165 | 1.113377 | 0.764946964 | 10 | 2 | 0.720229 |
| PPS_0789 | Coenzyme metabolism | 1.187631 | 3.021025 | 1.429421 | 2.0835 | 0 | 2.226755 | 0.764490306 | 11 | 3 | 0.657122 |
| PPS_0979 | Translation | 2.375261 | 1.510512 | 3.573552 | 0 | 3.468165 | 2.226755 | 0.763463022 | 13 | 4 | 0.649469 |
|  |  | 10.68868 | 3.021025 | 7.147104 | 6.2505 | 5.202247 | 4.453509 | 0.762641096 | 36 | 5 | 0.537192 |
| PPS_4355 | Cell envelope biogenesis | 1.187631 | 2.265768 | 3.573552 | 3.12525 | 0 | 2.226755 | 0.761639631 | 14 | 3 | 0.656635 |
| PPS_0705 | Cell envelope biogenesis | 2.375261 | 0.755256 | 1.429421 | 0 | 3.468165 | 0 | 0.760572752 | 7 | 5 | 0.791927 |
| PPS_4837 | Coenzyme metabolism | 7.125784 | 3.776281 | 5.002973 | 4.167 | 3.468165 | 4.453509 | 0.76005315 | 28 | 5 | 0.322467 |
| PPS_1847 | Amino acid transport and metabolism | 7.125784 | 6.042049 | 3.573552 | 4.167 | 5.202247 | 3.340132 | 0.759159345 | 29 | 7 | 0.338229 |
| PPS_0163 | Coenzyme metabolism | 10.68868 | 6.797305 | 7.147104 | 12.501 | 1.734082 | 4.453509 | 0.758678469 | 45 | 10 | 0.613234 |
| PPS_0952 | Function unknown | 0 | 3.021025 | 3.573552 | 1.04175 | 1.734082 | 2.226755 | 0.758591054 | 13 | 4 | 0.686407 |
| PPS_0076 | Inorganic ion transport and metabolism | 0 | 2.265768 | 2.144131 | 0 | 0 | 3.340132 | 0.757416721 | 9 | 3 | 0.804441 |
| PPS_0341 |  | 1.187631 | 1.510512 | 1.429421 | 3.12525 | 0 | 0 | 0.757165765 | 8 | 2 | 0.779285 |
| PPS_0333 | Energy production and conversion | 59.38153 | 81.56767 | 100.0595 | 59.37975 | 65.89513 | 56.78224 | 0.75539661 | 444 | 23 | 0.233043 |
| PPS_3604 | Energy production and conversion | 67.69495 | 64.95203 | 80.76228 | 42.71175 | 41.61798 | 76.82303 | 0.755134825 | 390 | 10 | 0.268747 |
| PPS_4158 | Translation | 13.06394 | 8.307818 | 12.86479 | 14.5845 | 3.468165 | 7.793641 | 0.754933295 | 63 | 10 | 0.494864 |
| PPS_4360 | Cell envelope biogenesis | 3.562892 | 5.286793 | 2.858842 | 3.12525 | 3.468165 | 2.226755 | 0.753311629 | 21 | 5 | 0.320803 |
| PPS_0330 | Posttranslational modification | 0 | 2.265768 | 1.429421 | 1.04175 | 1.734082 | 0 | 0.75120167 | 7 | 5 | 0.732413 |
| PPS_1722 | Defense mechanisms | 3.562892 | 3.021025 | 2.858842 | 3.12525 | 1.734082 | 2.226755 | 0.75042554 | 17 | 2 | 0.185379 |
| PPS_4242 |  | 3.562892 | 4.531537 | 3.573552 | 4.167 | 3.468165 | 1.113377 | 0.749790567 | 21 | 5 | 0.406754 |
| PPS_4665 | Nucleotide transport and metabolism | 9.501045 | 17.37089 | 15.00892 | 16.668 | 6.93633 | 7.793641 | 0.749697445 | 79 | 14 | 0.423274 |
| PPS_2776 | Secondary metabolites biosynthesis | 7.125784 | 8.307818 | 8.576525 | 8.334 | 5.202247 | 4.453509 | 0.749257034 | 44 | 6 | 0.227515 |
| PPS_1732 | Signal transduction mechanisms | 2.375261 | 4.531537 | 3.573552 | 1.04175 | 3.468165 | 3.340132 | 0.749025209 | 19 | 4 | 0.434755 |
| PPS_3587 | Energy production and conversion | 91.44756 | 129.9041 | 134.3656 | 83.34 | 84.97004 | 97.9772 | 0.748592573 | 654 | 24 | 0.149909 |
| PPS_0158 | Amino acid transport and metabolism | 16.62683 | 12.83935 | 10.72066 | 7.29225 | 13.87266 | 8.907018 | 0.748302863 | 69 | 9 | 0.269924 |
| PPS_4004 | Translation | 32.06603 | 33.98653 | 27.159 | 21.87675 | 27.74532 | 20.04079 | 0.74736295 | 165 | 21 | 0.064884 |
| PPS_1120 | Amino acid transport and metabolism | 13.06394 | 7.552562 | 20.01189 | 9.37575 | 8.670412 | 12.24715 | 0.745619299 | 74 | 8 | 0.443863 |
| PPS_3544 | Amino acid transport and metabolism | 3.562892 | 1.510512 | 2.858842 | 4.167 | 1.734082 | 0 | 0.743935888 | 14 | 3 | 0.651249 |
| PPS_0028 | Inorganic ion transport and metabolism | 0 | 0.755256 | 2.144131 | 1.04175 | 0 | 1.113377 | 0.743304344 | 6 | 4 | 0.753124 |
| PPS_2015 |  | 0 | 0.755256 | 2.144131 | 1.04175 | 0 | 1.113377 | 0.743304344 | 6 | 4 | 0.753124 |
| PPS_0062 | Inorganic ion transport and metabolism | 8.313414 | 6.797305 | 7.147104 | 6.2505 | 6.93633 | 3.340132 | 0.742523678 | 39 | 3 | 0.218818 |
| PPS_1206 | Carbohydrate transport and metabolism | 58.1939 | 105.7359 | 111.4948 | 61.46325 | 72.83146 | 70.14277 | 0.742262988 | 509 | 18 | 0.29466 |
| PPS_0193 | Energy production and conversion | 26.12787 | 15.86038 | 16.43834 | 18.7515 | 12.13858 | 12.24715 | 0.738314945 | 102 | 19 | 0.279982 |
| PPS_4171 | Posttranslational modification | 84.32177 | 94.40702 | 102.9183 | 64.5885 | 53.75655 | 89.07018 | 0.736436623 | 513 | 15 | 0.126099 |
| PPS_0580 | Secondary metabolites biosynthesis | 0 | 0.755256 | 5.002973 | 3.12525 | 0 | 1.113377 | 0.7360991 | 12 | 7 | 0.796096 |
| PPS_4885 | Carbohydrate transport and metabolism | 5.938153 | 5.286793 | 7.147104 | 2.0835 | 6.93633 | 4.453509 | 0.733360638 | 32 | 5 | 0.367971 |
| PPS_2027 | Carbohydrate transport and metabolism | 2.375261 | 1.510512 | 1.429421 | 1.04175 | 1.734082 | 1.113377 | 0.73171541 | 9 | 3 | 0.278774 |
| PPS_3249 | Secondary metabolites biosynthesis | 22.56498 | 15.86038 | 27.159 | 15.62625 | 15.60674 | 16.70066 | 0.730870173 | 117 | 11 | 0.213589 |
| PPS_5160 | Signal transduction mechanisms | 4.750523 | 1.510512 | 2.858842 | 2.0835 | 3.468165 | 1.113377 | 0.73082591 | 15 | 5 | 0.52354 |
| PPS_0274 | Amino acid transport and metabolism | 16.62683 | 10.57359 | 12.86479 | 10.4175 | 12.13858 | 6.680264 | 0.729719019 | 69 | 7 | 0.205965 |
| PPS_0037 | Amino acid transport and metabolism | 2.375261 | 0 | 1.429421 | 1.04175 | 1.734082 | 0 | 0.729583267 | 6 | 3 | 0.710566 |
| PPS_5143 | Translation | 1.187631 | 5.286793 | 5.717683 | 2.0835 | 3.468165 | 3.340132 | 0.729307617 | 23 | 5 | 0.531404 |
| PPS_5071 | Inorganic ion transport and metabolism | 10.68868 | 6.797305 | 9.291235 | 9.37575 | 3.468165 | 6.680264 | 0.729133976 | 48 | 9 | 0.31289 |
| PPS_1385 | General function prediction only | 0 | 2.265768 | 3.573552 | 3.12525 | 0 | 1.113377 | 0.725876786 | 12 | 4 | 0.720522 |
| PPS_0923 | General function prediction only | 1.187631 | 0.755256 | 4.288263 | 1.04175 | 3.468165 | 0 | 0.723769333 | 11 | 5 | 0.724167 |
| PPS_4138 |  | 3.562892 | 0.755256 | 0 | 3.12525 | 0 | 0 | 0.723747765 | 7 | 3 | 0.804487 |
| PPS_4130 | Cell envelope biogenesis | 5.938153 | 2.265768 | 4.288263 | 0 | 3.468165 | 5.566886 | 0.72325632 | 21 | 3 | 0.589204 |
| PPS_3863 | DNA replication | 2.375261 | 0 | 2.144131 | 1.04175 | 0 | 2.226755 | 0.723217672 | 8 | 4 | 0.696589 |
| PPS_0658 | Amino acid transport and metabolism | 2.375261 | 0.755256 | 0.71471 | 1.04175 | 1.734082 | 0 | 0.721890226 | 6 | 4 | 0.656893 |
| PPS_0898 | Translation | 2.375261 | 0.755256 | 0.71471 | 1.04175 | 1.734082 | 0 | 0.721890226 | 6 | 4 | 0.656893 |
| PPS_2571 | Amino acid transport and metabolism | 41.56707 | 28.69973 | 37.16494 | 30.21075 | 29.4794 | 17.81404 | 0.721427217 | 187 | 15 | 0.144852 |
| PPS_4820 | Coenzyme metabolism | 24.94024 | 23.41294 | 23.58544 | 17.70975 | 17.34082 | 16.70066 | 0.719380315 | 127 | 17 | 0.000791 |
| PPS_4604 | DNA replication | 4.750523 | 1.510512 | 2.144131 | 2.0835 | 1.734082 | 2.226755 | 0.719121652 | 14 | 5 | 0.511568 |
| PPS_3230 |  | 1.187631 | 3.776281 | 2.858842 | 1.04175 | 3.468165 | 1.113377 | 0.718837981 | 14 | 5 | 0.541594 |
| PPS_4968 | Coenzyme metabolism | 10.68868 | 6.797305 | 11.43537 | 9.37575 | 6.93633 | 4.453509 | 0.718002105 | 51 | 8 | 0.249878 |
| PPS_1310 | Function unknown | 2.375261 | 0.755256 | 1.429421 | 1.04175 | 0 | 2.226755 | 0.716787016 | 8 | 2 | 0.620093 |
| PPS_5070 | Nucleotide transport and metabolism | 5.938153 | 6.042049 | 7.861815 | 6.2505 | 3.468165 | 4.453509 | 0.714250664 | 36 | 6 | 0.144191 |
| PPS_3298 | Function unknown | 9.501045 | 6.042049 | 5.002973 | 6.2505 | 1.734082 | 6.680264 | 0.713754405 | 36 | 7 | 0.401649 |
| PPS_4958 | General function prediction only | 3.562892 | 6.042049 | 2.858842 | 2.0835 | 3.468165 | 3.340132 | 0.713410743 | 22 | 3 | 0.348887 |
| PPS_4364 | Cell envelope biogenesis | 4.750523 | 0 | 0.71471 | 1.04175 | 1.734082 | 1.113377 | 0.71162743 | 8 | 5 | 0.757609 |
| PPS_0160 | Coenzyme metabolism | 7.125784 | 6.797305 | 5.717683 | 7.29225 | 0 | 6.680264 | 0.711403462 | 36 | 7 | 0.505007 |
| PPS_1306 | Amino acid transport and metabolism | 2.375261 | 0.755256 | 0 | 0 | 0 | 2.226755 | 0.711305591 | 5 | 2 | 0.782619 |
| PPS_5032 | Amino acid transport and metabolism | 10.68868 | 11.32884 | 8.576525 | 6.2505 | 12.13858 | 3.340132 | 0.710243117 | 52 | 7 | 0.373701 |
| PPS_5214 | Cell envelope biogenesis | 2.375261 | 3.776281 | 2.858842 | 4.167 | 0 | 2.226755 | 0.709598471 | 17 | 4 | 0.55188 |
| PPS_0556 | Lipid metabolism | 15.4392 | 33.23127 | 28.58842 | 11.45925 | 12.13858 | 31.17456 | 0.708946156 | 143 | 7 | 0.423093 |
| PPS_2549 | Function unknown | 0 | 1.510512 | 1.429421 | 2.0835 | 0 | 0 | 0.708689579 | 6 | 2 | 0.755713 |
| PPS_0056 | General function prediction only | 0 | 1.510512 | 1.429421 | 2.0835 | 0 | 0 | 0.708689579 | 6 | 3 | 0.755713 |
| PPS_3615 | Lipid metabolism | 13.06394 | 6.042049 | 11.43537 | 6.2505 | 8.670412 | 6.680264 | 0.707276316 | 52 | 8 | 0.293777 |
| PPS_4755 | DNA replication | 5.938153 | 2.265768 | 3.573552 | 3.12525 | 5.202247 | 0 | 0.707069901 | 19 | 7 | 0.572247 |
| PPS_1583 | Lipid metabolism | 4.750523 | 12.0841 | 6.432394 | 6.2505 | 3.468165 | 6.680264 | 0.704814454 | 43 | 7 | 0.421298 |
| PPS_3574 | Lipid metabolism | 8.313414 | 18.8814 | 30.73255 | 11.45925 | 10.40449 | 18.92741 | 0.704177669 | 109 | 8 | 0.481499 |
| PPS_4779 | Defense mechanisms | 3.562892 | 5.286793 | 4.288263 | 4.167 | 1.734082 | 3.340132 | 0.703398622 | 24 | 9 | 0.218513 |
| PPS_3926 | Posttranslational modification | 2.375261 | 3.776281 | 2.858842 | 5.20875 | 0 | 1.113377 | 0.701649057 | 17 | 4 | 0.633056 |
|  |  | 38.00418 | 63.44152 | 63.60923 | 44.79525 | 32.94757 | 37.85483 | 0.700358638 | 301 | 20 | 0.182775 |
| PPS_1879 | Coenzyme metabolism | 3.562892 | 1.510512 | 3.573552 | 2.0835 | 1.734082 | 2.226755 | 0.699013238 | 15 | 5 | 0.33264 |
| PPS_5134 | Amino acid transport and metabolism | 3.562892 | 0.755256 | 2.144131 | 1.04175 | 3.468165 | 0 | 0.697882987 | 10 | 3 | 0.646405 |
| PPS_2779 | Amino acid transport and metabolism | 1.187631 | 0.755256 | 2.144131 | 0 | 1.734082 | 1.113377 | 0.696708365 | 7 | 4 | 0.562368 |
| PPS_4943 | Coenzyme metabolism | 3.562892 | 2.265768 | 2.858842 | 2.0835 | 1.734082 | 2.226755 | 0.695750849 | 15 | 4 | 0.13021 |
| PPS_0974 | Energy production and conversion | 38.00418 | 22.65768 | 32.16197 | 20.835 | 22.54307 | 21.15417 | 0.695211957 | 159 | 11 | 0.167816 |
| PPS_0408 | Amino acid transport and metabolism | 2.375261 | 1.510512 | 0.71471 | 2.0835 | 0 | 1.113377 | 0.694900203 | 8 | 2 | 0.577583 |
| PPS_4737 | General function prediction only | 11.87631 | 31.72076 | 31.44726 | 11.45925 | 29.4794 | 11.13377 | 0.693888903 | 134 | 5 | 0.440232 |
| PPS_1412 | Nucleotide transport and metabolism | 11.87631 | 3.021025 | 5.002973 | 5.20875 | 5.202247 | 3.340132 | 0.691000955 | 32 | 5 | 0.527705 |
| PPS_3842 | Translation | 3.562892 | 0 | 3.573552 | 2.0835 | 1.734082 | 1.113377 | 0.69095472 | 12 | 3 | 0.603233 |
| PPS_1527 | Defense mechanisms | 3.562892 | 1.510512 | 3.573552 | 3.12525 | 1.734082 | 1.113377 | 0.690729714 | 15 | 7 | 0.382797 |
| PPS_1660 | DNA replication | 2.375261 | 0.755256 | 0 | 1.04175 | 0 | 1.113377 | 0.688425261 | 5 | 2 | 0.707607 |
| PPS_1735 | Carbohydrate transport and metabolism | 9.501045 | 11.32884 | 7.861815 | 6.2505 | 3.468165 | 10.0204 | 0.687971043 | 51 | 9 | 0.257718 |
| PPS_0205 | Amino acid transport and metabolism | 24.94024 | 22.65768 | 23.58544 | 14.5845 | 24.27715 | 10.0204 | 0.686706005 | 121 | 13 | 0.216794 |
| PPS_3923 | Secondary metabolites biosynthesis | 16.62683 | 19.63666 | 18.58247 | 6.2505 | 19.07491 | 12.24715 | 0.685056043 | 94 | 12 | 0.257373 |
| PPS_0399 | Posttranslational modification | 8.313414 | 6.042049 | 5.717683 | 5.20875 | 5.202247 | 3.340132 | 0.685050976 | 34 | 6 | 0.114226 |
| PPS_5081 | Amino acid transport and metabolism | 10.68868 | 9.81833 | 12.15008 | 11.45925 | 8.670412 | 2.226755 | 0.684580939 | 57 | 8 | 0.335389 |
| PPS_0961 | Translation | 11.87631 | 15.86038 | 12.86479 | 13.54275 | 8.670412 | 5.566886 | 0.684212815 | 72 | 15 | 0.200483 |
| PPS_3614 | Amino acid transport and metabolism | 10.68868 | 9.063074 | 10.72066 | 8.334 | 6.93633 | 5.566886 | 0.683806063 | 53 | 8 | 0.035387 |
| PPS_0773 | Nucleotide transport and metabolism | 17.81446 | 15.10512 | 18.58247 | 13.54275 | 13.87266 | 7.793641 | 0.683643614 | 89 | 9 | 0.09201 |
| PPS_0839 | Posttranslational modification | 9.501045 | 3.776281 | 5.002973 | 6.2505 | 1.734082 | 4.453509 | 0.680409638 | 31 | 6 | 0.425691 |
| PPS_3993 | Function unknown | 3.562892 | 2.265768 | 0 | 0 | 1.734082 | 2.226755 | 0.679544987 | 9 | 3 | 0.646326 |
| PPS_1215 | Posttranslational modification | 2.375261 | 1.510512 | 0.71471 | 3.12525 | 0 | 0 | 0.679330696 | 8 | 2 | 0.69879 |
| PPS_3506 |  | 1.187631 | 1.510512 | 2.144131 | 1.04175 | 0 | 2.226755 | 0.67499369 | 9 | 2 | 0.513836 |
| PPS_1702 | DNA replication | 5.938153 | 6.797305 | 6.432394 | 3.12525 | 8.670412 | 1.113377 | 0.673473427 | 32 | 8 | 0.453661 |
| PPS_0986 | Secondary metabolites biosynthesis | 4.750523 | 0 | 0 | 2.0835 | 0 | 1.113377 | 0.672952767 | 7 | 3 | 0.782909 |
| PPS_0463 | Translation | 9.501045 | 18.12615 | 7.861815 | 6.2505 | 8.670412 | 8.907018 | 0.671417202 | 62 | 3 | 0.346389 |
| PPS_0967 | Translation | 7.125784 | 6.042049 | 10.00595 | 4.167 | 6.93633 | 4.453509 | 0.671312119 | 40 | 7 | 0.165953 |
|  |  | 34.44129 | 44.56011 | 47.17089 | 25.002 | 19.07491 | 40.08158 | 0.66701245 | 225 | 16 | 0.143722 |
| PPS_4304 |  | 2.375261 | 0 | 3.573552 | 0 | 1.734082 | 2.226755 | 0.665819667 | 10 | 2 | 0.628203 |
| PPS_1298 | Inorganic ion transport and metabolism | 1.187631 | 0.755256 | 2.858842 | 2.0835 | 0 | 1.113377 | 0.665776352 | 9 | 4 | 0.576039 |
| PPS_0008 | Carbohydrate transport and metabolism | 3.562892 | 0 | 0.71471 | 0 | 1.734082 | 1.113377 | 0.665667227 | 6 | 2 | 0.719371 |
| PPS_4366 | General function prediction only | 4.750523 | 5.286793 | 5.002973 | 2.0835 | 3.468165 | 4.453509 | 0.66522486 | 26 | 4 | 0.128524 |
| PPS_0286 | Amino acid transport and metabolism | 14.25157 | 4.531537 | 4.288263 | 7.29225 | 6.93633 | 1.113377 | 0.664978231 | 36 | 7 | 0.546558 |
| PPS_4347 | Amino acid transport and metabolism | 9.501045 | 9.81833 | 9.291235 | 5.20875 | 10.40449 | 3.340132 | 0.662459688 | 48 | 5 | 0.266804 |
| PPS_0432 | Translation | 5.938153 | 5.286793 | 6.432394 | 8.334 | 0 | 3.340132 | 0.661148948 | 32 | 9 | 0.497543 |
| PPS_4713 | Energy production and conversion | 8.313414 | 14.34987 | 11.43537 | 7.29225 | 5.202247 | 10.0204 | 0.660286955 | 61 | 12 | 0.162178 |
| PPS_4930 | Energy production and conversion | 52.25575 | 71.74934 | 66.46807 | 37.503 | 34.68165 | 53.44211 | 0.659550994 | 336 | 18 | 0.058713 |
| PPS_0024 | Translation | 2.375261 | 10.57359 | 7.861815 | 4.167 | 1.734082 | 7.793641 | 0.658062832 | 39 | 4 | 0.475249 |
| PPS_1199 | Cell envelope biogenesis | 4.750523 | 0.755256 | 2.858842 | 1.04175 | 0 | 4.453509 | 0.656964553 | 14 | 6 | 0.618649 |
|  |  | 1.187631 | 7.552562 | 7.147104 | 3.12525 | 1.734082 | 5.566886 | 0.656261357 | 30 | 7 | 0.492252 |
| PPS_5138 | Carbohydrate transport and metabolism | 5.938153 | 1.510512 | 0.71471 | 3.12525 | 0 | 2.226755 | 0.655611675 | 13 | 6 | 0.649189 |
| PPS_3981 | Amino acid transport and metabolism | 2.375261 | 3.021025 | 2.144131 | 2.0835 | 1.734082 | 1.113377 | 0.65393725 | 13 | 3 | 0.087961 |
| PPS_1662 | Cell envelope biogenesis | 3.562892 | 3.776281 | 2.858842 | 2.0835 | 3.468165 | 1.113377 | 0.653562724 | 17 | 5 | 0.220589 |
| PPS_1396 | Energy production and conversion | 8.313414 | 4.531537 | 7.147104 | 6.2505 | 3.468165 | 3.340132 | 0.653199294 | 34 | 4 | 0.192114 |
| PPS_1061 | Nucleotide transport and metabolism | 19.00209 | 19.63666 | 17.86776 | 17.70975 | 6.93633 | 12.24715 | 0.652902277 | 99 | 15 | 0.167126 |
| PPS_0281 | Cell envelope biogenesis | 1.187631 | 6.042049 | 2.144131 | 1.04175 | 1.734082 | 3.340132 | 0.65245225 | 17 | 8 | 0.556673 |
| PPS_1649 | Inorganic ion transport and metabolism | 0 | 4.531537 | 1.429421 | 1.04175 | 1.734082 | 1.113377 | 0.652447104 | 11 | 3 | 0.658768 |
| PPS_0220 | Amino acid transport and metabolism | 20.18972 | 14.34987 | 10.72066 | 7.29225 | 12.13858 | 10.0204 | 0.650708426 | 74 | 9 | 0.188056 |
| PPS_0185 |  | 7.125784 | 3.021025 | 3.573552 | 2.0835 | 3.468165 | 3.340132 | 0.648073102 | 22 | 4 | 0.338072 |
| PPS_3932 | Amino acid transport and metabolism | 5.938153 | 0 | 0.71471 | 2.0835 | 0 | 2.226755 | 0.647879593 | 10 | 2 | 0.726853 |
| PPS_1195 | Cell envelope biogenesis | 11.87631 | 6.797305 | 2.144131 | 2.0835 | 6.93633 | 4.453509 | 0.647204582 | 32 | 7 | 0.493596 |
| PPS_1405 | General function prediction only | 5.938153 | 6.797305 | 9.291235 | 5.20875 | 3.468165 | 5.566886 | 0.646660869 | 39 | 5 | 0.107601 |
| PPS_1212 | Lipid metabolism | 0 | 2.265768 | 2.144131 | 0 | 1.734082 | 1.113377 | 0.645697142 | 8 | 3 | 0.595047 |
| PPS_4288 | Nucleotide transport and metabolism | 3.562892 | 0.755256 | 2.144131 | 4.167 | 0 | 0 | 0.644818925 | 11 | 4 | 0.664678 |
| PPS_1526 | Posttranslational modification | 1.187631 | 2.265768 | 0 | 0 | 0 | 2.226755 | 0.644800809 | 6 | 2 | 0.700948 |
| PPS_0423 | Coenzyme metabolism | 4.750523 | 0.755256 | 2.144131 | 2.0835 | 1.734082 | 1.113377 | 0.644577479 | 12 | 3 | 0.523175 |
| PPS_1005 | Translation | 33.25366 | 26.43397 | 28.58842 | 15.62625 | 15.60674 | 25.60768 | 0.643896902 | 150 | 27 | 0.067221 |
| PPS_1956 | Function unknown | 3.562892 | 4.531537 | 5.717683 | 2.0835 | 3.468165 | 3.340132 | 0.643768053 | 24 | 4 | 0.105789 |
| PPS_1723 | Cell envelope biogenesis | 0 | 3.776281 | 1.429421 | 0 | 0 | 3.340132 | 0.641629512 | 10 | 4 | 0.711518 |
| PPS_3650 | Function unknown | 7.125784 | 0.755256 | 1.429421 | 3.12525 | 1.734082 | 1.113377 | 0.641505271 | 14 | 3 | 0.643156 |
|  |  | 2.375261 | 2.265768 | 1.429421 | 1.04175 | 1.734082 | 1.113377 | 0.64067891 | 10 | 3 | 0.127737 |
| PPS_4778 | Cell envelope biogenesis | 8.313414 | 9.063074 | 8.576525 | 3.12525 | 3.468165 | 10.0204 | 0.640149564 | 45 | 11 | 0.29918 |
| PPS_0509 | Function unknown | 1.187631 | 0.755256 | 1.429421 | 1.04175 | 0 | 1.113377 | 0.639066035 | 6 | 2 | 0.393434 |
| PPS_0916 | General function prediction only | 0 | 2.265768 | 2.858842 | 1.04175 | 0 | 2.226755 | 0.637805497 | 10 | 5 | 0.600814 |
| PPS_3165 | General function prediction only | 2.375261 | 6.797305 | 3.573552 | 4.167 | 1.734082 | 2.226755 | 0.637671518 | 23 | 7 | 0.381125 |
| PPS_0668 | Amino acid transport and metabolism | 34.44129 | 65.70729 | 50.02973 | 31.2525 | 27.74532 | 36.74145 | 0.637503992 | 265 | 8 | 0.17469 |
| PPS_1422 | Cell envelope biogenesis | 5.938153 | 6.797305 | 7.861815 | 6.2505 | 3.468165 | 3.340132 | 0.634006086 | 36 | 5 | 0.100351 |
| PPS_4974 | Amino acid transport and metabolism | 13.06394 | 11.32884 | 12.15008 | 7.29225 | 6.93633 | 8.907018 | 0.633108628 | 62 | 14 | 0.00526 |
| PPS_4521 | Amino acid transport and metabolism | 95.01045 | 123.1068 | 118.6419 | 65.63025 | 69.3633 | 77.93641 | 0.632291548 | 582 | 19 | 0.027924 |
| PPS_0688 | Translation | 2.375261 | 3.776281 | 2.858842 | 0 | 3.468165 | 2.226755 | 0.632039599 | 15 | 2 | 0.396104 |
| PPS_1561 | Amino acid transport and metabolism | 13.06394 | 24.1682 | 22.87073 | 20.835 | 10.40449 | 6.680264 | 0.630914289 | 107 | 13 | 0.252182 |
| PPS_4576 | Inorganic ion transport and metabolism | 20.18972 | 10.57359 | 11.43537 | 12.501 | 5.202247 | 8.907018 | 0.630594827 | 70 | 4 | 0.244151 |
|  | Amino acid transport and metabolism | 9.501045 | 7.552562 | 9.291235 | 5.20875 | 6.93633 | 4.453509 | 0.630050792 | 44 | 5 | 0.028942 |
| PPS_3708 | Inorganic ion transport and metabolism | 4.750523 | 8.307818 | 4.288263 | 5.20875 | 3.468165 | 2.226755 | 0.628576641 | 30 | 4 | 0.243639 |
| PPS_0182 |  | 3.562892 | 1.510512 | 3.573552 | 2.0835 | 0 | 3.340132 | 0.627230162 | 15 | 8 | 0.42346 |
| PPS_4299 | General function prediction only | 3.562892 | 0 | 1.429421 | 3.12525 | 0 | 0 | 0.626012465 | 8 | 3 | 0.693532 |
| PPS_4219 | Posttranslational modification | 13.06394 | 7.552562 | 5.002973 | 5.20875 | 5.202247 | 5.566886 | 0.623661716 | 41 | 7 | 0.309111 |
| PPS_3815 | Inorganic ion transport and metabolism | 5.938153 | 4.531537 | 3.573552 | 4.167 | 3.468165 | 1.113377 | 0.62297167 | 23 | 5 | 0.205732 |
| PPS_1509 | Amino acid transport and metabolism | 5.938153 | 5.286793 | 4.288263 | 5.20875 | 0 | 4.453509 | 0.62284078 | 27 | 8 | 0.353468 |
| PPS_4739 | DNA replication | 4.750523 | 0 | 4.288263 | 1.04175 | 3.468165 | 1.113377 | 0.622129195 | 14 | 5 | 0.552616 |
| PPS_5038 | Amino acid transport and metabolism | 2.375261 | 3.776281 | 7.147104 | 2.0835 | 1.734082 | 4.453509 | 0.621949881 | 24 | 4 | 0.379609 |
| PPS_0832 | Posttranslational modification | 4.750523 | 2.265768 | 2.144131 | 0 | 3.468165 | 2.226755 | 0.621687425 | 14 | 2 | 0.433317 |
| PPS_0808 | General function prediction only | 4.750523 | 6.042049 | 6.432394 | 6.2505 | 0 | 4.453509 | 0.621424119 | 31 | 7 | 0.363092 |
| PPS_4345 | Coenzyme metabolism | 2.375261 | 4.531537 | 2.144131 | 1.04175 | 3.468165 | 1.113377 | 0.621294428 | 15 | 3 | 0.35833 |
| PPS_4560 | Function unknown | 0 | 3.021025 | 2.144131 | 2.0835 | 0 | 1.113377 | 0.618931417 | 10 | 2 | 0.580901 |
| PPS_1082 | Nucleotide transport and metabolism | 26.12787 | 43.0496 | 45.02676 | 20.835 | 17.34082 | 32.28794 | 0.616997845 | 201 | 28 | 0.129458 |
| PPS_4330 | Signal transduction mechanisms | 2.375261 | 1.510512 | 1.429421 | 1.04175 | 0 | 2.226755 | 0.614936027 | 9 | 4 | 0.411512 |
| PPS_4869 | Inorganic ion transport and metabolism | 5.938153 | 5.286793 | 4.288263 | 2.0835 | 5.202247 | 2.226755 | 0.613187242 | 25 | 9 | 0.178063 |
| PPS_0930 | Amino acid transport and metabolism | 14.25157 | 4.531537 | 10.00595 | 6.2505 | 6.93633 | 4.453509 | 0.612744721 | 46 | 8 | 0.316238 |
| PPS_3559 | Signal transduction mechanisms | 0 | 0 | 2.858842 | 0 | 1.734082 | 0 | 0.606568179 | 5 | 3 | 0.756872 |
| PPS_0975 | Inorganic ion transport and metabolism | 8.313414 | 7.552562 | 3.573552 | 2.0835 | 5.202247 | 4.453509 | 0.603885866 | 31 | 2 | 0.227141 |
| PPS_4216 | Intracellular trafficking and secretion | 7.125784 | 6.042049 | 2.144131 | 4.167 | 1.734082 | 3.340132 | 0.603528979 | 25 | 6 | 0.317113 |
| PPS_3413 | Cell envelope biogenesis | 1.187631 | 2.265768 | 0 | 2.0835 | 0 | 0 | 0.603318623 | 6 | 3 | 0.65732 |
| PPS_4335 | Inorganic ion transport and metabolism | 2.375261 | 1.510512 | 4.288263 | 2.0835 | 1.734082 | 1.113377 | 0.603246621 | 14 | 6 | 0.318175 |
| PPS_5145 | Nucleotide transport and metabolism | 0 | 2.265768 | 1.429421 | 0 | 0 | 2.226755 | 0.602609052 | 7 | 2 | 0.648617 |
| PPS_0428 | Amino acid transport and metabolism | 11.87631 | 15.10512 | 15.00892 | 10.4175 | 10.40449 | 4.453509 | 0.601936026 | 71 | 9 | 0.088149 |
| PPS_4176 | Amino acid transport and metabolism | 4.750523 | 3.776281 | 5.002973 | 4.167 | 1.734082 | 2.226755 | 0.60073698 | 23 | 7 | 0.120367 |
| PPS_4214 | General function prediction only | 2.375261 | 2.265768 | 3.573552 | 2.0835 | 1.734082 | 1.113377 | 0.600269101 | 14 | 7 | 0.105907 |
| PPS_4973 | General function prediction only | 7.125784 | 8.307818 | 2.858842 | 4.167 | 3.468165 | 3.340132 | 0.599990742 | 30 | 5 | 0.277146 |
| PPS_3959 | Function unknown | 4.750523 | 0 | 0 | 0 | 1.734082 | 1.113377 | 0.599399257 | 6 | 2 | 0.733947 |
| PPS_4949 | Nucleotide transport and metabolism | 22.56498 | 17.37089 | 22.87073 | 5.20875 | 3.468165 | 28.94781 | 0.599056779 | 107 | 4 | 0.415549 |
| PPS_1733 | Signal transduction mechanisms | 0 | 0.755256 | 2.144131 | 0 | 1.734082 | 0 | 0.598085782 | 5 | 3 | 0.672773 |
| PPS_1397 | Function unknown | 4.750523 | 2.265768 | 2.858842 | 4.167 | 1.734082 | 0 | 0.597569935 | 16 | 4 | 0.413857 |
| PPS_0335 | Posttranslational modification | 7.125784 | 8.307818 | 10.72066 | 9.37575 | 1.734082 | 4.453509 | 0.595059571 | 46 | 17 | 0.253283 |
| PPS_0763 | Energy production and conversion | 0 | 1.510512 | 1.429421 | 0 | 1.734082 | 0 | 0.589837352 | 5 | 3 | 0.624748 |
| PPS_0822 |  | 16.62683 | 15.10512 | 16.43834 | 8.334 | 12.13858 | 7.793641 | 0.586797726 | 79 | 11 | 0.029103 |
| PPS_0912 | Posttranslational modification | 5.938153 | 5.286793 | 5.002973 | 7.29225 | 0 | 2.226755 | 0.586581952 | 28 | 11 | 0.40905 |
| PPS_1558 | Function unknown | 4.750523 | 1.510512 | 1.429421 | 1.04175 | 3.468165 | 0 | 0.586430113 | 11 | 4 | 0.51901 |
| PPS_0972 | General function prediction only | 3.562892 | 3.776281 | 3.573552 | 4.167 | 0 | 2.226755 | 0.585898999 | 19 | 6 | 0.33727 |
| PPS_4626 | Coenzyme metabolism | 2.375261 | 0 | 1.429421 | 0 | 0 | 2.226755 | 0.585266911 | 6 | 2 | 0.631373 |
| PPS_1213 | Function unknown | 2.375261 | 0 | 1.429421 | 0 | 0 | 2.226755 | 0.585266911 | 6 | 3 | 0.631373 |
| PPS_1192 | Lipid metabolism | 1.187631 | 2.265768 | 1.429421 | 0 | 1.734082 | 1.113377 | 0.583158849 | 8 | 2 | 0.333569 |
| PPS_1903 | Function unknown | 3.562892 | 3.021025 | 2.858842 | 1.04175 | 0 | 4.453509 | 0.581954865 | 16 | 4 | 0.431633 |
| PPS_2768 | Carbohydrate transport and metabolism | 0 | 3.021025 | 0.71471 | 1.04175 | 0 | 1.113377 | 0.576895104 | 7 | 2 | 0.633262 |
| PPS_3782 | acyl-carrier-protein | 9.501045 | 4.531537 | 5.002973 | 4.167 | 3.468165 | 3.340132 | 0.57656825 | 30 | 7 | 0.229775 |
| PPS_4599 | Lipid metabolism | 5.938153 | 0 | 1.429421 | 3.12525 | 0 | 1.113377 | 0.575308409 | 11 | 3 | 0.63985 |
| PPS_4561 | Carbohydrate transport and metabolism | 3.562892 | 3.021025 | 2.858842 | 2.0835 | 0 | 3.340132 | 0.574369446 | 16 | 4 | 0.30127 |
| PPS_3639 | Function unknown | 8.313414 | 6.042049 | 5.002973 | 2.0835 | 3.468165 | 5.566886 | 0.574351707 | 31 | 5 | 0.122824 |
| PPS_0768 | Energy production and conversion | 4.750523 | 2.265768 | 3.573552 | 2.0835 | 1.734082 | 2.226755 | 0.570767373 | 17 | 4 | 0.164595 |
| PPS_3776 |  | 11.87631 | 13.59461 | 12.86479 | 10.4175 | 6.93633 | 4.453509 | 0.568851904 | 64 | 8 | 0.076186 |
| PPS_4664 | Nucleotide transport and metabolism | 21.37735 | 23.41294 | 31.44726 | 15.62625 | 8.670412 | 18.92741 | 0.566965689 | 130 | 13 | 0.06316 |
| PPS_3334 | Inorganic ion transport and metabolism | 10.68868 | 6.042049 | 7.147104 | 3.12525 | 10.40449 | 0 | 0.566623719 | 36 | 5 | 0.388314 |
| PPS_3742 | Cell motility and secretion | 17.81446 | 24.92345 | 10.72066 | 11.45925 | 12.13858 | 6.680264 | 0.566384228 | 87 | 8 | 0.191341 |
| PPS_0021 | Lipid metabolism | 0 | 2.265768 | 1.429421 | 2.0835 | 0 | 0 | 0.563841203 | 7 | 2 | 0.605343 |
| PPS_3831 | Amino acid transport and metabolism | 15.4392 | 13.59461 | 7.147104 | 6.2505 | 5.202247 | 8.907018 | 0.562721156 | 58 | 8 | 0.159026 |
| PPS_4627 | Coenzyme metabolism | 4.750523 | 7.552562 | 7.861815 | 6.2505 | 1.734082 | 3.340132 | 0.561605309 | 35 | 6 | 0.15454 |
| PPS_4732 | Nucleotide transport and metabolism | 24.94024 | 56.64421 | 35.73552 | 20.835 | 27.74532 | 16.70066 | 0.556435309 | 197 | 12 | 0.195586 |
| PPS_4750 | Inorganic ion transport and metabolism | 3.562892 | 4.531537 | 3.573552 | 3.12525 | 0 | 3.340132 | 0.55411316 | 20 | 7 | 0.245056 |
| PPS_3010 | General function prediction only | 14.25157 | 12.0841 | 14.29421 | 9.37575 | 8.670412 | 4.453509 | 0.55377161 | 66 | 13 | 0.041079 |
| PPS_0490 | Amino acid transport and metabolism | 3.562892 | 1.510512 | 2.858842 | 1.04175 | 0 | 3.340132 | 0.552413764 | 13 | 5 | 0.374859 |
|  |  | 3.562892 | 1.510512 | 0.71471 | 2.0835 | 0 | 1.113377 | 0.552317546 | 9 | 3 | 0.457784 |
| PPS_2383 | Transcription | 3.562892 | 4.531537 | 5.002973 | 1.04175 | 1.734082 | 4.453509 | 0.551967603 | 22 | 5 | 0.192391 |
| PPS_2030 | Transcription | 4.750523 | 4.531537 | 7.861815 | 4.167 | 5.202247 | 0 | 0.546506998 | 28 | 3 | 0.257376 |
| PPS_1513 | General function prediction only | 7.125784 | 6.042049 | 7.861815 | 4.167 | 1.734082 | 5.566886 | 0.545323864 | 35 | 9 | 0.086597 |
| PPS_0654 |  | 1.187631 | 0.755256 | 2.144131 | 0 | 0 | 2.226755 | 0.544835993 | 7 | 2 | 0.515788 |
| PPS_1008 | Amino acid transport and metabolism | 49.88049 | 80.05715 | 87.90938 | 32.29425 | 43.35206 | 42.30834 | 0.541456318 | 365 | 19 | 0.092189 |
| PPS_5183 | Nucleotide transport and metabolism | 1.187631 | 4.531537 | 5.717683 | 0 | 1.734082 | 4.453509 | 0.541022305 | 20 | 5 | 0.403865 |
| PPS_0334 | Energy production and conversion | 30.8784 | 92.89651 | 58.60625 | 41.67 | 27.74532 | 28.94781 | 0.539327242 | 313 | 17 | 0.255611 |
| PPS_4987 | Inorganic ion transport and metabolism | 7.125784 | 9.81833 | 7.147104 | 3.12525 | 8.670412 | 1.113377 | 0.535840039 | 38 | 11 | 0.235633 |
| PPS_0794 |  | 4.750523 | 3.021025 | 2.858842 | 0 | 3.468165 | 2.226755 | 0.535720698 | 16 | 2 | 0.251087 |
| PPS_5049 | Inorganic ion transport and metabolism | 3.562892 | 0 | 3.573552 | 2.0835 | 1.734082 | 0 | 0.534941828 | 11 | 5 | 0.471922 |
| PPS_0426 | General function prediction only | 7.125784 | 1.510512 | 6.432394 | 5.20875 | 1.734082 | 1.113377 | 0.534632388 | 24 | 5 | 0.349358 |
|  | Defense mechanisms | 7.125784 | 3.021025 | 4.288263 | 3.12525 | 3.468165 | 1.113377 | 0.533893605 | 22 | 12 | 0.203964 |
| PPS_0045 | Amino acid transport and metabolism | 23.75261 | 28.69973 | 27.159 | 19.79325 | 10.40449 | 12.24715 | 0.533151343 | 132 | 14 | 0.031604 |
| PPS_5144 | Function unknown | 8.313414 | 5.286793 | 7.147104 | 3.12525 | 3.468165 | 4.453509 | 0.532450855 | 33 | 7 | 0.049429 |
| PPS_0541 | Energy production and conversion | 0 | 4.531537 | 5.002973 | 0 | 1.734082 | 3.340132 | 0.532194549 | 17 | 2 | 0.478618 |
| PPS_3626 | Energy production and conversion | 3.562892 | 0.755256 | 2.858842 | 2.0835 | 1.734082 | 0 | 0.531919723 | 11 | 2 | 0.354678 |
| PPS_3414 | Cell division and chromosome partitioning | 4.750523 | 3.021025 | 1.429421 | 3.12525 | 1.734082 | 0 | 0.528132735 | 14 | 4 | 0.334013 |
| PPS_0044 | Amino acid transport and metabolism | 9.501045 | 9.81833 | 10.00595 | 5.20875 | 6.93633 | 3.340132 | 0.528049169 | 47 | 6 | 0.044656 |
| PPS_2334 |  | 9.501045 | 5.286793 | 7.861815 | 5.20875 | 0 | 6.680264 | 0.52490931 | 37 | 6 | 0.219288 |
| PPS_3980 | Amino acid transport and metabolism | 10.68868 | 7.552562 | 11.43537 | 2.0835 | 3.468165 | 10.0204 | 0.524725141 | 48 | 8 | 0.18583 |
| PPS_2810 | General function prediction only | 2.375261 | 2.265768 | 3.573552 | 2.0835 | 0 | 2.226755 | 0.524707723 | 14 | 4 | 0.209932 |
| PPS_4827 |  | 3.562892 | 2.265768 | 2.144131 | 4.167 | 0 | 0 | 0.522652565 | 13 | 3 | 0.46273 |
| PPS_3424 | Translation | 2.375261 | 1.510512 | 3.573552 | 1.04175 | 1.734082 | 1.113377 | 0.521388907 | 12 | 5 | 0.175471 |
| PPS_0995 | Amino acid transport and metabolism | 2.375261 | 1.510512 | 3.573552 | 1.04175 | 1.734082 | 1.113377 | 0.521388907 | 12 | 5 | 0.175471 |
| PPS_0361 | Coenzyme metabolism | 1.187631 | 0 | 2.144131 | 0 | 1.734082 | 0 | 0.520470085 | 5 | 2 | 0.56412 |
| PPS_4470 |  | 2.375261 | 2.265768 | 3.573552 | 3.12525 | 0 | 1.113377 | 0.515988196 | 14 | 5 | 0.284969 |
| PPS_5086 |  | 4.750523 | 5.286793 | 1.429421 | 4.167 | 1.734082 | 0 | 0.514626145 | 18 | 3 | 0.338373 |
| PPS_0058 | Amino acid transport and metabolism | 21.37735 | 15.86038 | 20.01189 | 12.501 | 6.93633 | 10.0204 | 0.514548808 | 92 | 21 | 0.016053 |
| PPS_2741 | Transcription | 3.562892 | 1.510512 | 1.429421 | 0 | 0 | 3.340132 | 0.513643191 | 10 | 2 | 0.475254 |
| PPS_5140 | Nucleotide transport and metabolism | 0 | 6.042049 | 3.573552 | 2.0835 | 1.734082 | 1.113377 | 0.512808243 | 17 | 4 | 0.468155 |
| PPS_0009 | DNA replication | 1.187631 | 3.021025 | 0 | 1.04175 | 0 | 1.113377 | 0.512070276 | 7 | 2 | 0.529252 |
| PPS_4841 | Nucleotide transport and metabolism | 5.938153 | 1.510512 | 0.71471 | 4.167 | 0 | 0 | 0.510450585 | 12 | 3 | 0.567706 |
| PPS_3617 | Amino acid transport and metabolism | 62.94442 | 69.48357 | 72.90046 | 27.0855 | 52.02247 | 25.60768 | 0.509990931 | 326 | 19 | 0.04752 |
| PPS_0145 |  | 1.187631 | 0.755256 | 2.144131 | 2.0835 | 0 | 0 | 0.509784877 | 7 | 4 | 0.464233 |
| PPS_0293 |  | 4.750523 | 0 | 0.71471 | 1.04175 | 1.734082 | 0 | 0.507907427 | 7 | 3 | 0.614317 |
| PPS_0513 | Coenzyme metabolism | 8.313414 | 12.0841 | 18.58247 | 7.29225 | 3.468165 | 8.907018 | 0.504552105 | 66 | 9 | 0.153053 |
| PPS_3600 | Cell envelope biogenesis | 4.750523 | 6.042049 | 4.288263 | 3.12525 | 0 | 4.453509 | 0.502542426 | 25 | 4 | 0.189828 |
| PPS_4940 | Amino acid transport and metabolism | 7.125784 | 5.286793 | 7.147104 | 3.12525 | 0 | 6.680264 | 0.50131255 | 32 | 6 | 0.228643 |
| PPS_2986 | Amino acid transport and metabolism | 1.187631 | 3.021025 | 3.573552 | 1.04175 | 1.734082 | 1.113377 | 0.499756621 | 13 | 4 | 0.207457 |
| PPS_3924 | Energy production and conversion | 4.750523 | 4.531537 | 1.429421 | 3.12525 | 0 | 2.226755 | 0.49965125 | 17 | 2 | 0.277731 |
| PPS_5083 | Function unknown | 3.562892 | 2.265768 | 2.144131 | 0 | 1.734082 | 2.226755 | 0.496794237 | 12 | 3 | 0.185674 |
| PPS_1546 | Lipid metabolism | 7.125784 | 11.32884 | 7.147104 | 4.167 | 5.202247 | 3.340132 | 0.496426561 | 41 | 6 | 0.076581 |
| PPS_3610 | Defense mechanisms | 1.187631 | 3.021025 | 0 | 2.0835 | 0 | 0 | 0.495051237 | 7 | 4 | 0.563152 |
| PPS_1376 | Cell division and chromosome partitioning | 3.562892 | 3.021025 | 2.144131 | 2.0835 | 0 | 2.226755 | 0.493839475 | 14 | 3 | 0.168562 |
| PPS_1595 | Energy production and conversion | 0 | 1.510512 | 2.858842 | 1.04175 | 0 | 1.113377 | 0.493237047 | 8 | 4 | 0.47787 |
|  |  | 2.375261 | 0 | 2.144131 | 0 | 0 | 2.226755 | 0.492711029 | 7 | 3 | 0.510685 |
| PPS_3851 | Signal transduction mechanisms | 0 | 3.776281 | 2.858842 | 1.04175 | 0 | 2.226755 | 0.49260651 | 12 | 2 | 0.45057 |
| PPS_3927 | Amino acid transport and metabolism | 10.68868 | 2.265768 | 2.858842 | 2.0835 | 3.468165 | 2.226755 | 0.491891402 | 22 | 7 | 0.428343 |
| PPS_3779 | Cell motility and secretion | 5.938153 | 0 | 0.71471 | 1.04175 | 0 | 2.226755 | 0.491292886 | 9 | 2 | 0.616384 |
| PPS_4829 | Function unknown | 0 | 2.265768 | 2.144131 | 1.04175 | 0 | 1.113377 | 0.4887021 | 8 | 3 | 0.428501 |
| PPS_3261 | Function unknown | 7.125784 | 18.12615 | 12.15008 | 5.20875 | 5.202247 | 7.793641 | 0.486728886 | 62 | 2 | 0.175051 |
| PPS_4394 | Amino acid transport and metabolism | 8.313414 | 4.531537 | 2.144131 | 2.0835 | 5.202247 | 0 | 0.486070253 | 21 | 6 | 0.337136 |
| PPS_4871 | General function prediction only | 3.562892 | 3.021025 | 7.861815 | 4.167 | 1.734082 | 1.113377 | 0.485573183 | 24 | 7 | 0.253083 |
| PPS_1175 | Amino acid transport and metabolism | 2.375261 | 2.265768 | 2.144131 | 1.04175 | 0 | 2.226755 | 0.48171363 | 11 | 3 | 0.208912 |
| PPS_3852 | Energy production and conversion | 5.938153 | 4.531537 | 5.717683 | 2.0835 | 3.468165 | 2.226755 | 0.480523869 | 25 | 6 | 0.010635 |
| PPS_2848 | Function unknown | 0 | 3.776281 | 0.71471 | 1.04175 | 0 | 1.113377 | 0.479877862 | 8 | 3 | 0.577189 |
| PPS_3891 | acyl-carrier-protein | 3.562892 | 2.265768 | 2.858842 | 4.167 | 0 | 0 | 0.479654562 | 14 | 7 | 0.392741 |
| PPS_1503 | Amino acid transport and metabolism | 0 | 1.510512 | 2.858842 | 2.0835 | 0 | 0 | 0.476843944 | 8 | 4 | 0.520102 |
|  | Translation | 3.562892 | 2.265768 | 0 | 1.04175 | 1.734082 | 0 | 0.476238487 | 8 | 2 | 0.446024 |
| PPS_3084 | Energy production and conversion | 4.750523 | 3.776281 | 2.144131 | 0 | 1.734082 | 3.340132 | 0.475517319 | 16 | 5 | 0.207159 |
| PPS_4706 | General function prediction only | 2.375261 | 2.265768 | 3.573552 | 1.04175 | 1.734082 | 1.113377 | 0.473451934 | 13 | 3 | 0.054951 |
| PPS_3321 | General function prediction only | 8.313414 | 8.307818 | 6.432394 | 5.20875 | 3.468165 | 2.226755 | 0.47296982 | 36 | 10 | 0.022755 |
| PPS_4825 | Function unknown | 23.75261 | 28.69973 | 32.16197 | 15.62625 | 12.13858 | 12.24715 | 0.47287479 | 136 | 7 | 0.013538 |
| PPS_3832 | Lipid metabolism | 23.75261 | 33.98653 | 31.44726 | 16.668 | 12.13858 | 13.36053 | 0.472797475 | 144 | 20 | 0.02259 |
| PPS_1667 |  | 0 | 2.265768 | 2.144131 | 2.0835 | 0 | 0 | 0.472459719 | 8 | 2 | 0.486293 |
| PPS_0557 | Amino acid transport and metabolism | 2.375261 | 2.265768 | 1.429421 | 0 | 1.734082 | 1.113377 | 0.469068914 | 9 | 2 | 0.158698 |
| PPS_3702 | Carbohydrate transport and metabolism | 4.750523 | 0 | 0 | 0 | 0 | 2.226755 | 0.468738867 | 6 | 2 | 0.66512 |
| PPS_1683 | Cell motility and secretion | 4.750523 | 3.776281 | 0.71471 | 2.0835 | 0 | 2.226755 | 0.466401356 | 14 | 3 | 0.322947 |
| PPS_4536 | Signal transduction mechanisms | 16.62683 | 26.43397 | 16.43834 | 11.45925 | 6.93633 | 8.907018 | 0.458873866 | 95 | 4 | 0.067552 |
| PPS_1551 | General function prediction only | 4.750523 | 1.510512 | 0.71471 | 2.0835 | 0 | 1.113377 | 0.458284692 | 10 | 3 | 0.428724 |
| PPS_4640 | Cell envelope biogenesis | 4.750523 | 3.776281 | 7.861815 | 4.167 | 0 | 3.340132 | 0.458069852 | 27 | 4 | 0.170194 |
| PPS_0515 | Transcription | 2.375261 | 0.755256 | 1.429421 | 2.0835 | 0 | 0 | 0.456914081 | 7 | 3 | 0.387722 |
| PPS_4122 | Amino acid transport and metabolism | 54.63101 | 42.29435 | 48.60031 | 25.002 | 20.80899 | 20.04079 | 0.452509739 | 224 | 15 | 0.008636 |
| PPS_4789 | General function prediction only | 2.375261 | 0.755256 | 0.71471 | 0 | 1.734082 | 0 | 0.450969999 | 5 | 3 | 0.426602 |
| PPS_1515 |  | 2.375261 | 4.531537 | 2.858842 | 1.04175 | 0 | 3.340132 | 0.448704012 | 16 | 4 | 0.214491 |
| PPS_3520 | Energy production and conversion | 7.125784 | 5.286793 | 11.43537 | 2.0835 | 5.202247 | 3.340132 | 0.445567935 | 37 | 8 | 0.120999 |
| PPS_1411 | Amino acid transport and metabolis | 10.68868 | 14.34987 | 20.7266 | 3.12525 | 10.40449 | 6.680264 | 0.441602622 | 72 | 15 | 0.08431 |
| PPS_0165 | Transcription | 4.750523 | 5.286793 | 5.002973 | 3.12525 | 3.468165 | 0 | 0.438383529 | 23 | 4 | 0.122662 |
| PPS_3749 | Cell motility and secretion | 7.125784 | 1.510512 | 6.432394 | 3.12525 | 3.468165 | 0 | 0.437557269 | 22 | 7 | 0.259251 |
| PPS_4733 | Amino acid transport and metabolism | 28.50314 | 26.43397 | 20.7266 | 12.501 | 13.87266 | 6.680264 | 0.436853094 | 114 | 10 | 0.011473 |
| PPS_1027 | Amino acid transport and metabolism | 20.18972 | 21.90243 | 23.58544 | 10.4175 | 10.40449 | 7.793641 | 0.435698597 | 102 | 15 | 0.000756 |
| PPS_0565 | Amino acid transport and metabolism | 3.562892 | 2.265768 | 2.144131 | 0 | 3.468165 | 0 | 0.435000054 | 11 | 6 | 0.324978 |
| PPS_4762 | DNA replication | 1.187631 | 3.776281 | 1.429421 | 1.04175 | 1.734082 | 0 | 0.43417615 | 10 | 3 | 0.293466 |
| PPS_1860 | Cell motility and secretion | 2.375261 | 4.531537 | 2.858842 | 3.12525 | 0 | 1.113377 | 0.434034769 | 16 | 4 | 0.183931 |
| PPS_3032 |  | 0 | 3.021025 | 2.144131 | 0 | 0 | 2.226755 | 0.431110808 | 9 | 4 | 0.449189 |
| PPS_2628 | General function prediction only | 4.750523 | 3.021025 | 1.429421 | 0 | 1.734082 | 2.226755 | 0.43048046 | 13 | 2 | 0.218467 |
| PPS_0985 | Cell envelope biogenesis | 7.125784 | 1.510512 | 2.858842 | 2.0835 | 1.734082 | 1.113377 | 0.428960466 | 16 | 5 | 0.324538 |
| PPS_0035 | Energy production and conversion | 2.375261 | 6.042049 | 5.717683 | 2.0835 | 1.734082 | 2.226755 | 0.427615107 | 23 | 6 | 0.146011 |
| PPS_4723 | Transcription | 8.313414 | 5.286793 | 4.288263 | 3.12525 | 0 | 4.453509 | 0.423667259 | 27 | 12 | 0.127949 |
| PPS_3838 | Energy production and conversion | 8.313414 | 6.797305 | 5.717683 | 3.12525 | 3.468165 | 2.226755 | 0.42346834 | 31 | 12 | 0.018687 |
| PPS_2301 |  | 3.562892 | 4.531537 | 3.573552 | 2.0835 | 1.734082 | 1.113377 | 0.422606077 | 18 | 3 | 0.006605 |
| PPS_3636 | Translation | 2.375261 | 5.286793 | 1.429421 | 2.0835 | 1.734082 | 0 | 0.419907913 | 14 | 4 | 0.273935 |
| PPS_3612 |  | 8.313414 | 8.307818 | 6.432394 | 5.20875 | 0 | 4.453509 | 0.419121013 | 36 | 13 | 0.096491 |
| PPS_1714 | DNA replication | 0 | 3.021025 | 2.144131 | 1.04175 | 0 | 1.113377 | 0.417243411 | 9 | 6 | 0.385291 |
| PPS_1599 | Carbohydrate transport and metabolism | 4.750523 | 4.531537 | 3.573552 | 3.12525 | 0 | 2.226755 | 0.416316602 | 20 | 6 | 0.100246 |
| PPS_3987 | Function unknown | 4.750523 | 2.265768 | 5.002973 | 1.04175 | 1.734082 | 2.226755 | 0.416214084 | 18 | 8 | 0.101142 |
| PPS_0583 | Cell motility and secretion | 4.750523 | 8.307818 | 5.717683 | 2.0835 | 3.468165 | 2.226755 | 0.414274048 | 29 | 8 | 0.058505 |
| PPS_4867 | Amino acid transport and metabolism | 16.62683 | 4.531537 | 10.72066 | 4.167 | 3.468165 | 5.566886 | 0.41412974 | 46 | 6 | 0.213806 |
| PPS_0835 | Cell motility and secretion | 4.750523 | 3.776281 | 0.71471 | 2.0835 | 1.734082 | 0 | 0.413090593 | 13 | 2 | 0.279196 |
| PPS_3703 | Lipid metabolism | 0 | 1.510512 | 3.573552 | 2.0835 | 0 | 0 | 0.409809912 | 9 | 3 | 0.47346 |
| PPS_3360 | DNA replication | 2.375261 | 3.021025 | 1.429421 | 1.04175 | 1.734082 | 0 | 0.406673257 | 10 | 7 | 0.120119 |
| PPS_1983 |  | 7.125784 | 1.510512 | 2.144131 | 2.0835 | 0 | 2.226755 | 0.399822233 | 15 | 3 | 0.352144 |
| PPS_2782 |  | 8.313414 | 3.776281 | 5.717683 | 3.12525 | 1.734082 | 2.226755 | 0.397929819 | 26 | 6 | 0.102049 |
| PPS_4842 | Nucleotide transport and metabolism | 4.750523 | 10.57359 | 10.72066 | 6.2505 | 1.734082 | 2.226755 | 0.39206869 | 42 | 8 | 0.102189 |
| PPS_3105 |  | 8.313414 | 3.021025 | 2.144131 | 4.167 | 0 | 1.113377 | 0.391760932 | 19 | 3 | 0.309648 |
| PPS_2752 | Energy production and conversion | 3.562892 | 2.265768 | 5.002973 | 3.12525 | 0 | 1.113377 | 0.391319309 | 17 | 4 | 0.144707 |
| PPS_4847 | Lipid metabolism | 8.313414 | 6.042049 | 7.861815 | 4.167 | 0 | 4.453509 | 0.388009231 | 34 | 11 | 0.06897 |
|  |  | 2.375261 | 3.021025 | 2.858842 | 2.0835 | 0 | 1.113377 | 0.387259583 | 13 | 5 | 0.095808 |
| PPS_1736 | Translation | 8.313414 | 3.021025 | 2.858842 | 1.04175 | 0 | 4.453509 | 0.387173282 | 20 | 3 | 0.270284 |
| PPS_3951 | Translation | 1.187631 | 2.265768 | 2.144131 | 1.04175 | 0 | 1.113377 | 0.385013948 | 9 | 3 | 0.081767 |
| PPS_2028 | Nucleotide transport and metabolism | 1.187631 | 2.265768 | 2.144131 | 1.04175 | 0 | 1.113377 | 0.385013948 | 9 | 4 | 0.081767 |
| PPS_1678 | General function prediction only | 0 | 0.755256 | 2.144131 | 0 | 0 | 1.113377 | 0.384004311 | 5 | 2 | 0.470002 |
| PPS_3760 | Cell motility and secretion | 1.187631 | 3.021025 | 1.429421 | 1.04175 | 0 | 1.113377 | 0.382245153 | 9 | 2 | 0.175691 |
| PPS_0905 | Translation | 0 | 1.510512 | 1.429421 | 0 | 0 | 1.113377 | 0.378708361 | 5 | 2 | 0.382131 |
| PPS_1028 | Amino acid transport and metabolism | 74.82073 | 82.32292 | 65.75336 | 25.002 | 38.14981 | 21.15417 | 0.378228406 | 329 | 31 | 0.002825 |
| PPS_1179 | Amino acid transport and metabolism | 2.375261 | 0.755256 | 4.288263 | 1.04175 | 1.734082 | 0 | 0.374162924 | 11 | 3 | 0.269538 |
| PPS_1047 | Carbohydrate transport and metabolism | 0 | 2.265768 | 0.71471 | 0 | 0 | 1.113377 | 0.3735565 | 5 | 4 | 0.473201 |
| PPS_0879 | Inorganic ion transport and metabolism | 8.313414 | 2.265768 | 4.288263 | 2.0835 | 3.468165 | 0 | 0.373410807 | 20 | 5 | 0.221247 |
| PPS_3822 | Amino acid transport and metabolism | 3.562892 | 3.021025 | 5.002973 | 2.0835 | 0 | 2.226755 | 0.371994101 | 18 | 4 | 0.062037 |
| PPS_3998 | Intracellular trafficking and secretion | 2.375261 | 2.265768 | 2.858842 | 1.04175 | 1.734082 | 0 | 0.370117331 | 11 | 3 | 0.075233 |
| PPS_4374 | Energy production and conversion | 1.187631 | 3.021025 | 1.429421 | 2.0835 | 0 | 0 | 0.369540949 | 9 | 4 | 0.261384 |
| PPS_0130 |  | 4.750523 | 3.021025 | 4.288263 | 0 | 0 | 4.453509 | 0.369285187 | 18 | 3 | 0.223773 |
| PPS_2428 | Energy production and conversion | 2.375261 | 2.265768 | 4.288263 | 1.04175 | 0 | 2.226755 | 0.366042956 | 14 | 4 | 0.109383 |
| PPS_0869 | Amino acid transport and metabolism | 5.938153 | 3.021025 | 9.291235 | 2.0835 | 3.468165 | 1.113377 | 0.365199514 | 27 | 8 | 0.155703 |
| PPS_3421 | Energy production and conversion | 26.12787 | 14.34987 | 13.5795 | 9.37575 | 6.93633 | 3.340132 | 0.363544491 | 76 | 11 | 0.089344 |
| PPS_1892 | Cell motility and secretion | 5.938153 | 6.797305 | 2.144131 | 3.12525 | 0 | 2.226755 | 0.359687638 | 22 | 6 | 0.147655 |
| PPS_3024 |  | 3.562892 | 2.265768 | 0 | 2.0835 | 0 | 0 | 0.35745778 | 8 | 3 | 0.382628 |
| PPS_1155 | Energy production and conversion | 9.501045 | 5.286793 | 13.5795 | 7.29225 | 1.734082 | 1.113377 | 0.357443138 | 43 | 13 | 0.123973 |
| PPS_0390 | Translation | 4.750523 | 3.021025 | 4.288263 | 2.0835 | 0 | 2.226755 | 0.357406514 | 18 | 3 | 0.048894 |
| PPS_4295 | Energy production and conversion | 7.125784 | 6.797305 | 5.717683 | 4.167 | 1.734082 | 1.113377 | 0.357137665 | 29 | 7 | 0.029789 |
| PPS_1416 | Translation | 2.375261 | 2.265768 | 1.429421 | 1.04175 | 0 | 1.113377 | 0.355019325 | 9 | 4 | 0.051155 |
| PPS_0157 | Posttranslational modification | 4.750523 | 3.776281 | 4.288263 | 1.04175 | 3.468165 | 0 | 0.351922873 | 18 | 5 | 0.10566 |
| PPS_4093 |  | 3.562892 | 6.042049 | 2.858842 | 1.04175 | 0 | 3.340132 | 0.351569171 | 19 | 4 | 0.122767 |
| PPS_0479 | DNA replication | 3.562892 | 3.021025 | 4.288263 | 2.0835 | 1.734082 | 0 | 0.351133142 | 16 | 5 | 0.046631 |
| PPS_3189 | Amino acid transport and metabolism | 0 | 2.265768 | 0.71471 | 1.04175 | 0 | 0 | 0.349524366 | 5 | 2 | 0.454057 |
| PPS_1217 | Transcription | 10.68868 | 9.063074 | 12.86479 | 5.20875 | 1.734082 | 4.453509 | 0.349403783 | 49 | 7 | 0.009793 |
| PPS_4794 | General function prediction only | 2.375261 | 0.755256 | 2.858842 | 2.0835 | 0 | 0 | 0.347866936 | 9 | 3 | 0.239528 |
| PPS_0373 | Coenzyme metabolism | 5.938153 | 2.265768 | 2.858842 | 2.0835 | 1.734082 | 0 | 0.34508398 | 15 | 2 | 0.157248 |
| PPS_1009 | General function prediction only | 3.562892 | 1.510512 | 1.429421 | 0 | 0 | 2.226755 | 0.342428794 | 9 | 2 | 0.23466 |
| PPS_0358 | Coenzyme metabolism | 1.187631 | 6.797305 | 7.861815 | 2.0835 | 0 | 3.340132 | 0.342255135 | 26 | 4 | 0.230998 |
| PPS_2017 |  | 1.187631 | 2.265768 | 2.858842 | 1.04175 | 0 | 1.113377 | 0.341420319 | 10 | 3 | 0.090496 |
| PPS_3955 | Amino acid transport and metabolism | 0 | 1.510512 | 3.573552 | 0 | 1.734082 | 0 | 0.341081908 | 8 | 4 | 0.41318 |
| PPS_5158 | Transcription | 4.750523 | 5.286793 | 2.858842 | 2.0835 | 0 | 2.226755 | 0.334227819 | 19 | 6 | 0.049863 |
| PPS_1197 | Lipid metabolism | 1.187631 | 0.755256 | 1.429421 | 0 | 0 | 1.113377 | 0.330152937 | 5 | 3 | 0.169712 |
| PPS_0140 |  | 2.375261 | 2.265768 | 2.144131 | 0 | 0 | 2.226755 | 0.328180059 | 10 | 2 | 0.176281 |
| PPS_4293 | Carbohydrate transport and metabolism | 57.00627 | 89.12023 | 102.2036 | 21.87675 | 34.68165 | 24.4943 | 0.326390969 | 372 | 23 | 0.04424 |
| PPS_2408 | Energy production and conversion | 7.125784 | 2.265768 | 4.288263 | 0 | 0 | 4.453509 | 0.325553317 | 19 | 7 | 0.207608 |
| PPS_3508 | Translation | 5.938153 | 23.41294 | 6.432394 | 2.0835 | 1.734082 | 7.793641 | 0.324485509 | 55 | 12 | 0.294114 |
| PPS_0030 | Translation | 3.562892 | 0.755256 | 2.144131 | 2.0835 | 0 | 0 | 0.322409462 | 9 | 4 | 0.244837 |
| PPS_5177 | Inorganic ion transport and metabolism | 2.375261 | 3.776281 | 0.71471 | 1.04175 | 0 | 1.113377 | 0.313872418 | 10 | 5 | 0.210781 |
| PPS_2800 | Cell division and chromosome partitioning | 1.187631 | 0 | 2.144131 | 1.04175 | 0 | 0 | 0.312672403 | 5 | 2 | 0.358307 |
| PPS_3020 |  | 1.187631 | 0 | 2.144131 | 1.04175 | 0 | 0 | 0.312672403 | 5 | 3 | 0.358307 |
| PPS_4128 | General function prediction only | 1.187631 | 1.510512 | 2.858842 | 0 | 1.734082 | 0 | 0.31205456 | 8 | 5 | 0.175223 |
| PPS_1219 | DNA replication | 5.938153 | 2.265768 | 4.288263 | 1.04175 | 1.734082 | 1.113377 | 0.311331439 | 17 | 7 | 0.108573 |
|  | Energy production and conversion | 1.187631 | 0.755256 | 1.429421 | 1.04175 | 0 | 0 | 0.308913098 | 5 | 2 | 0.142039 |
| PPS_4993 | Signal transduction mechanisms | 5.938153 | 6.797305 | 5.002973 | 2.0835 | 0 | 3.340132 | 0.305755996 | 26 | 10 | 0.032888 |
| PPS_3550 |  | 1.187631 | 1.510512 | 0.71471 | 1.04175 | 0 | 0 | 0.305243116 | 5 | 4 | 0.141578 |
| PPS_5279 | Energy production and conversion | 0 | 1.510512 | 2.144131 | 0 | 0 | 1.113377 | 0.304647291 | 6 | 2 | 0.328178 |
| PPS_4197 | Cell envelope biogenesis | 0 | 1.510512 | 2.144131 | 0 | 0 | 1.113377 | 0.304647291 | 6 | 4 | 0.328178 |
| PPS_1285 |  | 0 | 1.510512 | 2.144131 | 0 | 0 | 1.113377 | 0.304647291 | 6 | 2 | 0.328178 |
| PPS_4849 | Lipid metabolism | 5.938153 | 4.531537 | 5.717683 | 2.0835 | 1.734082 | 1.113377 | 0.30461765 | 23 | 7 | 0.003464 |
| PPS_4886 | Carbohydrate transport and metabolism | 1.187631 | 6.797305 | 2.858842 | 1.04175 | 0 | 2.226755 | 0.301417514 | 17 | 9 | 0.265213 |
| PPS_3285 | Energy production and conversion | 0 | 2.265768 | 1.429421 | 0 | 0 | 1.113377 | 0.301304526 | 6 | 2 | 0.335537 |
| PPS_4731 | Cell motility and secretion | 7.125784 | 6.042049 | 1.429421 | 1.04175 | 0 | 3.340132 | 0.300185353 | 20 | 9 | 0.183546 |
| PPS_5047 | Amino acid transport and metabolism | 3.562892 | 1.510512 | 2.144131 | 1.04175 | 0 | 1.113377 | 0.298596007 | 10 | 3 | 0.089861 |
| PPS_1133 | Energy production and conversion | 17.81446 | 9.063074 | 10.72066 | 2.0835 | 3.468165 | 5.566886 | 0.295720385 | 51 | 10 | 0.066835 |
| PPS_0283 |  | 1.187631 | 3.776281 | 2.144131 | 2.0835 | 0 | 0 | 0.293118666 | 11 | 5 | 0.178559 |
| PPS_3202 | Cell envelope biogenesis | 2.375261 | 0.755256 | 0.71471 | 0 | 0 | 1.113377 | 0.289547802 | 5 | 2 | 0.249357 |
| PPS_0605 | Posttranslational modification | 2.375261 | 0.755256 | 2.858842 | 0 | 1.734082 | 0 | 0.289527204 | 8 | 4 | 0.174878 |
| PPS_1727 | Function unknown | 3.562892 | 2.265768 | 1.429421 | 2.0835 | 0 | 0 | 0.287059339 | 10 | 2 | 0.138587 |
| PPS_4203 | Cell envelope biogenesis | 3.562892 | 2.265768 | 1.429421 | 2.0835 | 0 | 0 | 0.287059339 | 10 | 3 | 0.138587 |
| PPS_0957 | Function unknown | 0 | 1.510512 | 2.144131 | 1.04175 | 0 | 0 | 0.285048316 | 6 | 2 | 0.313201 |
| PPS_4548 | Lipid metabolism | 0 | 1.510512 | 2.144131 | 1.04175 | 0 | 0 | 0.285048316 | 6 | 2 | 0.313201 |
| PPS_0359 | Coenzyme metabolism | 0 | 1.510512 | 2.144131 | 1.04175 | 0 | 0 | 0.285048316 | 6 | 2 | 0.313201 |
| PPS_0984 | General function prediction only | 0 | 2.265768 | 1.429421 | 1.04175 | 0 | 0 | 0.281920602 | 6 | 2 | 0.321098 |
| PPS_1407 | Translation | 0 | 2.265768 | 1.429421 | 1.04175 | 0 | 0 | 0.281920602 | 6 | 2 | 0.321098 |
| PPS_5002 | Secondary metabolites biosynthesis | 4.750523 | 3.021025 | 7.861815 | 1.04175 | 0 | 3.340132 | 0.280290437 | 23 | 2 | 0.103691 |
| PPS_1368 | Inorganic ion transport and metabolism | 8.313414 | 3.776281 | 3.573552 | 1.04175 | 0 | 3.340132 | 0.279755642 | 21 | 6 | 0.122177 |
| PPS_2584 | General function prediction only | 0 | 3.021025 | 0.71471 | 1.04175 | 0 | 0 | 0.278860781 | 6 | 3 | 0.435333 |
| PPS_1909 | Transcription | 7.125784 | 9.063074 | 11.43537 | 4.167 | 3.468165 | 0 | 0.276393815 | 40 | 6 | 0.020565 |
| PPS_3274 | Amino acid transport and metabolism | 2.375261 | 0 | 1.429421 | 1.04175 | 0 | 0 | 0.27380737 | 5 | 3 | 0.320328 |
| PPS_4285 |  | 7.125784 | 6.042049 | 1.429421 | 0 | 1.734082 | 2.226755 | 0.271341237 | 19 | 4 | 0.169204 |
| PPS_5033 | Amino acid transport and metabolism | 2.375261 | 3.021025 | 2.858842 | 0 | 0 | 2.226755 | 0.26974199 | 12 | 4 | 0.105388 |
| PPS_0828 | General function prediction only | 2.375261 | 1.510512 | 0 | 1.04175 | 0 | 0 | 0.268093335 | 5 | 2 | 0.310666 |
| PPS_4863 | Cell motility and secretion | 3.562892 | 3.776281 | 0.71471 | 1.04175 | 0 | 1.113377 | 0.267588595 | 11 | 3 | 0.175122 |
| PPS_4438 |  | 3.562892 | 1.510512 | 2.858842 | 2.0835 | 0 | 0 | 0.262662054 | 11 | 3 | 0.102614 |
| PPS_0265 | Inorganic ion transport and metabolism | 4.750523 | 8.307818 | 6.432394 | 1.04175 | 1.734082 | 2.226755 | 0.25666488 | 28 | 12 | 0.031998 |
| PPS_5059 | Function unknown | 1.187631 | 0.755256 | 2.144131 | 1.04175 | 0 | 0 | 0.254892439 | 6 | 3 | 0.133951 |
| PPS_4868 | Cell envelope biogenesis | 3.562892 | 3.776281 | 3.573552 | 1.04175 | 1.734082 | 0 | 0.254366572 | 15 | 7 | 0.030833 |
| PPS_5206 | Coenzyme metabolism | 0 | 2.265768 | 2.144131 | 0 | 0 | 1.113377 | 0.25247224 | 7 | 2 | 0.275862 |
| PPS_4517 | DNA replication | 0 | 2.265768 | 2.144131 | 0 | 0 | 1.113377 | 0.25247224 | 7 | 4 | 0.275862 |
| PPS_3333 | DNA replication | 0 | 3.021025 | 1.429421 | 0 | 0 | 1.113377 | 0.250172095 | 7 | 2 | 0.333746 |
| PPS_4029 | Signal transduction mechanisms | 0 | 3.021025 | 1.429421 | 0 | 0 | 1.113377 | 0.250172095 | 7 | 5 | 0.333746 |
| PPS_4835 | Signal transduction mechanisms | 4.750523 | 2.265768 | 0 | 0 | 1.734082 | 0 | 0.247150866 | 8 | 3 | 0.330876 |
| PPS_3824 | Amino acid transport and metabolism | 2.375261 | 0 | 2.144131 | 0 | 0 | 1.113377 | 0.246355515 | 6 | 2 | 0.273015 |
| PPS_1645 | Function unknown | 0 | 4.531537 | 0 | 0 | 0 | 1.113377 | 0.245695286 | 7 | 2 | 0.532845 |
| PPS_1185 | Translation | 5.938153 | 6.042049 | 6.432394 | 1.04175 | 3.468165 | 0 | 0.244936387 | 25 | 7 | 0.043222 |
| PPS_0391 | Function unknown | 2.375261 | 1.510512 | 0.71471 | 0 | 0 | 1.113377 | 0.242013073 | 6 | 3 | 0.132174 |
| PPS_4650 | Function unknown | 3.562892 | 0.755256 | 0 | 1.04175 | 0 | 0 | 0.241249255 | 5 | 2 | 0.4235 |
| PPS_4565 | Translation | 0 | 2.265768 | 2.144131 | 1.04175 | 0 | 0 | 0.23622986 | 7 | 2 | 0.265957 |
| PPS_0994 | Amino acid transport and metabolism | 5.938153 | 1.510512 | 6.432394 | 1.04175 | 0 | 2.226755 | 0.235465066 | 19 | 6 | 0.13929 |
| PPS_3448 | Carbohydrate transport and metabolism | 4.750523 | 2.265768 | 2.144131 | 1.04175 | 0 | 1.113377 | 0.235265057 | 12 | 3 | 0.094754 |
| PPS_1108 | Nucleotide transport and metabolism | 2.375261 | 6.797305 | 7.147104 | 2.0835 | 1.734082 | 0 | 0.233925207 | 24 | 8 | 0.097483 |
| PPS_3685 | Nucleotide transport and metabolism | 1.187631 | 0.755256 | 2.858842 | 0 | 0 | 1.113377 | 0.231870102 | 7 | 4 | 0.189849 |
| PPS_1216 | Cell envelope biogenesis | 2.375261 | 3.021025 | 2.144131 | 0 | 1.734082 | 0 | 0.229971679 | 10 | 3 | 0.06093 |
| PPS_1525 | Lipid metabolism | 2.375261 | 0.755256 | 1.429421 | 1.04175 | 0 | 0 | 0.22845704 | 6 | 3 | 0.121204 |
| PPS_1181 | Inorganic ion transport and metabolism | 2.375261 | 0.755256 | 1.429421 | 1.04175 | 0 | 0 | 0.22845704 | 6 | 3 | 0.121204 |
| PPS_0290 | Amino acid transport and metabolism | 2.375261 | 0.755256 | 1.429421 | 1.04175 | 0 | 0 | 0.22845704 | 6 | 3 | 0.121204 |
| PPS_4346 | Posttranslational modification | 3.562892 | 3.021025 | 2.858842 | 1.04175 | 0 | 1.113377 | 0.228230695 | 13 | 3 | 0.008118 |
| PPS_4707 | Amino acid transport and metabolism | 4.750523 | 0.755256 | 2.144131 | 0 | 1.734082 | 0 | 0.226680106 | 9 | 3 | 0.230615 |
| PPS_3680 | Cell division and chromosome partitioning | 2.375261 | 1.510512 | 0.71471 | 1.04175 | 0 | 0 | 0.226443565 | 6 | 5 | 0.122463 |
| PPS_0380 | Function unknown | 2.375261 | 1.510512 | 0.71471 | 1.04175 | 0 | 0 | 0.226443565 | 6 | 3 | 0.122463 |
| PPS_0914 | Function unknown | 2.375261 | 2.265768 | 0 | 1.04175 | 0 | 0 | 0.224465271 | 6 | 3 | 0.259207 |
| PPS_0771 | Translation | 3.562892 | 0 | 1.429421 | 0 | 0 | 1.113377 | 0.223018335 | 6 | 4 | 0.339256 |
| PPS_0820 | Lipid metabolism | 13.06394 | 7.552562 | 11.43537 | 3.12525 | 1.734082 | 2.226755 | 0.221081889 | 43 | 8 | 0.030364 |
| PPS_3745 | Cell motility and secretion | 1.187631 | 5.286793 | 1.429421 | 0 | 1.734082 | 0 | 0.219397329 | 11 | 3 | 0.25904 |
| PPS_2749 | Transcription | 0 | 1.510512 | 3.573552 | 0 | 0 | 1.113377 | 0.21899354 | 8 | 5 | 0.330261 |
| PPS_4711 | Amino acid transport and metabolism | 19.00209 | 13.59461 | 12.15008 | 4.167 | 0 | 5.566886 | 0.217532675 | 60 | 9 | 0.013286 |
| PPS_3079 | Defense mechanisms | 1.187631 | 1.510512 | 2.144131 | 1.04175 | 0 | 0 | 0.215136515 | 7 | 5 | 0.049442 |
| PPS_4484 | Cytoplasmic | 1.187631 | 1.510512 | 2.144131 | 1.04175 | 0 | 0 | 0.215136515 | 7 | 3 | 0.049442 |
| PPS_0187 | Cell envelope biogenesis | 0 | 0.755256 | 4.288263 | 1.04175 | 0 | 0 | 0.206552223 | 8 | 5 | 0.42109 |
| PPS_0154 | Inorganic ion transport and metabolism | 3.562892 | 1.510512 | 0 | 1.04175 | 0 | 0 | 0.205335502 | 6 | 2 | 0.322604 |
| PPS_4539 |  | 0 | 9.81833 | 1.429421 | 0 | 0 | 2.226755 | 0.197973315 | 17 | 5 | 0.431468 |
| PPS_1605 | General function prediction only | 2.375261 | 2.265768 | 0.71471 | 1.04175 | 0 | 0 | 0.19451093 | 7 | 3 | 0.098753 |
| PPS_2305 | Amino acid transport and metabolism | 5.938153 | 3.021025 | 7.147104 | 3.12525 | 0 | 0 | 0.194039195 | 22 | 7 | 0.056079 |
| PPS_1675 | Cell motility and secretion | 2.375261 | 4.531537 | 2.144131 | 0 | 1.734082 | 0 | 0.191591637 | 12 | 4 | 0.067422 |
| PPS_3336 | Coenzyme metabolism | 5.938153 | 4.531537 | 4.288263 | 1.04175 | 1.734082 | 0 | 0.188090615 | 19 | 4 | 0.005172 |
| PPS_5195 | Energy production and conversion | 47.50523 | 52.86793 | 51.45915 | 8.334 | 8.670412 | 11.13377 | 0.185324094 | 205 | 31 | 0.000151 |
| PPS_1048 | Cell envelope biogenesis | 8.313414 | 2.265768 | 0.71471 | 2.0835 | 0 | 0 | 0.184480227 | 13 | 5 | 0.315417 |
| PPS_4247 | Carbohydrate transport and metabolism | 2.375261 | 1.510512 | 2.144131 | 1.04175 | 0 | 0 | 0.17276392 | 8 | 4 | 0.021354 |
| PPS_4666 | Signal transduction mechanisms | 3.562892 | 2.265768 | 0.71471 | 0 | 0 | 1.113377 | 0.170153474 | 8 | 3 | 0.145935 |
| PPS_1520 |  | 4.750523 | 0 | 1.429421 | 1.04175 | 0 | 0 | 0.168569506 | 7 | 2 | 0.347421 |
| PPS_0002 | Cell division and chromosome partitioning | 2.375261 | 2.265768 | 2.144131 | 0 | 0 | 1.113377 | 0.16409003 | 9 | 2 | 0.032992 |
| PPS_1322 |  | 8.313414 | 2.265768 | 2.858842 | 1.04175 | 0 | 1.113377 | 0.160375304 | 16 | 5 | 0.186354 |
| PPS_5050 | Coenzyme metabolism | 3.562892 | 1.510512 | 1.429421 | 1.04175 | 0 | 0 | 0.160199604 | 8 | 2 | 0.103697 |
| PPS_1680 | Amino acid transport and metabolism | 1.187631 | 3.021025 | 2.858842 | 0 | 0 | 1.113377 | 0.157534878 | 10 | 3 | 0.055948 |
| PPS_4454 | Energy production and conversion | 0 | 3.021025 | 4.288263 | 0 | 0 | 1.113377 | 0.152323646 | 11 | 4 | 0.24152 |
| PPS_0925 | General function prediction only | 0 | 4.531537 | 2.858842 | 0 | 0 | 1.113377 | 0.150652263 | 11 | 3 | 0.250724 |
| PPS_4745 | RNA processing and modification | 2.375261 | 3.021025 | 2.858842 | 0 | 0 | 1.113377 | 0.134870995 | 11 | 4 | 0.010626 |
| PPS_1126 | Energy production and conversion | 2.375261 | 2.265768 | 3.573552 | 1.04175 | 0 | 0 | 0.126817167 | 11 | 4 | 0.012659 |
| PPS_2527 | Cell motility and secretion | 3.562892 | 3.776281 | 2.144131 | 0 | 0 | 1.113377 | 0.117403942 | 12 | 3 | 0.014238 |
| PPS_1039 | Amino acid transport and metabolism | 21.37735 | 15.86038 | 17.86776 | 1.04175 | 1.734082 | 3.340132 | 0.110986475 | 69 | 17 | 0.003996 |
| PPS_0572 | Signal transduction mechanisms | 3.562892 | 3.021025 | 2.858842 | 1.04175 | 0 | 0 | 0.110322638 | 12 | 5 | 0.004528 |
| PPS_4488 | Cell motility and secretion | 3.562892 | 6.042049 | 0.71471 | 0 | 0 | 1.113377 | 0.107889037 | 13 | 5 | 0.178772 |
| PPS_2212 | Signal transduction mechanisms | 4.750523 | 3.021025 | 2.858842 | 0 | 0 | 1.113377 | 0.104735329 | 13 | 3 | 0.016836 |
| PPS_1053 | Carbohydrate transport and metabolism | 27.3155 | 17.37089 | 22.87073 | 0 | 6.93633 | 0 | 0.102673539 | 82 | 8 | 0.006165 |
| PPS_2183 | Carbohydrate transport and metabolism | 54.63101 | 55.1337 | 79.33286 | 7.29225 | 8.670412 | 3.340132 | 0.102078489 | 245 | 19 | 0.017156 |
| PPS_5196 | Lipid metabolism | 14.25157 | 15.86038 | 21.44131 | 1.04175 | 1.734082 | 2.226755 | 0.097037258 | 67 | 15 | 0.017205 |
| PPS_1051 | Carbohydrate transport and metabolism | 3.562892 | 4.531537 | 5.717683 | 0 | 0 | 1.113377 | 0.080608762 | 18 | 9 | 0.007919 |
| PPS_0065 |  | 5.938153 | 3.776281 | 4.288263 | 1.04175 | 0 | 0 | 0.074396385 | 17 | 6 | 0.009527 |
| PPS_1040 | Carbohydrate transport and metabolism | 4.750523 | 6.797305 | 6.432394 | 0 | 0 | 1.113377 | 0.061922332 | 23 | 9 | 0.00348 |
| PPS_2748 | Carbohydrate transport and metabolism | 13.06394 | 7.552562 | 7.861815 | 0 | 1.734082 | 0 | 0.060891331 | 33 | 9 | 0.028511 |
| PPS_4182 | Energy production and conversion | 41.56707 | 43.0496 | 48.60031 | 0 | 5.202247 | 1.113377 | 0.047408554 | 164 | 25 | 0.000158 |
| PPS_1038 | Carbohydrate transport and metabolism | 83.13414 | 148.7855 | 121.5008 | 3.12525 | 5.202247 | 2.226755 | 0.029863167 | 445 | 14 | 0.02644 |
| PPS_4180 | Energy production and conversion | 35.62892 | 33.98653 | 35.73552 | 3.12525 | 0 | 0 | 0.029665129 | 128 | 17 | 7.49E-05 |
| PPS_2226 | Carbohydrate transport and metabolism | 46.31759 | 51.35742 | 46.45618 | 4.167 | 0 | 0 | 0.028911161 | 176 | 16 | 3.46E-05 |
| PPS_1052 | Carbohydrate transport and metabolism | 14.25157 | 19.63666 | 12.15008 | 1.04175 | 0 | 0 | 0.022627897 | 56 | 7 | 0.019367 |
| PPS_0005 | General function prediction only | 5.938153 | 1.510512 | 1.429421 | 0 | 0 | 0 | 0 | 9 | 3 | 0.185324 |
| PPS_1694 | General function prediction only | 3.562892 | 2.265768 | 2.144131 | 0 | 0 | 0 | 0 | 9 | 2 | 0.027966 |
| PPS_3407 | Amino acid transport and metabolism | 1.187631 | 0.755256 | 2.144131 | 0 | 0 | 0 | 0 | 5 | 3 | 0.079989 |
| PPS_4110 | Secondary metabolites biosynthesis | 1.187631 | 2.265768 | 0.71471 | 0 | 0 | 0 | 0 | 5 | 2 | 0.093995 |
| PPS_0175 |  | 2.375261 | 0.755256 | 1.429421 | 0 | 0 | 0 | 0 | 5 | 2 | 0.083725 |
| PPS_3737 | Coenzyme metabolism | 8.313414 | 4.531537 | 3.573552 | 0 | 0 | 0 | 0 | 18 | 2 | 0.063334 |
| PPS_3734 | Secondary metabolites biosynthesis | 5.938153 | 6.797305 | 5.717683 | 0 | 0 | 0 | 0 | 22 | 5 | 0.002854 |
| PPS_3378 | Energy production and conversion | 0 | 3.776281 | 2.858842 | 0 | 0 | 0 | 0 | 9 | 4 | 0.191198 |
| PPS_2801 | Cell envelope biogenesis | 2.375261 | 3.776281 | 0.71471 | 0 | 0 | 0 | 0 | 8 | 2 | 0.122583 |
| PPS_2021 | Carbohydrate transport and metabolism | 3.562892 | 0.755256 | 0.71471 | 0 | 0 | 0 | 0 | 5 | 2 | 0.217108 |
| PPS_5045 | Amino acid transport and metabolism | 1.187631 | 2.265768 | 0.71471 | 0 | 0 | 0 | 0 | 5 | 2 | 0.093995 |
| PPS_5023 | Inorganic ion transport and metabolism | 0 | 3.021025 | 0.71471 | 0 | 0 | 0 | 0 | 5 | 3 | 0.305239 |
| PPS_1535 | Cell envelope biogenesis | 3.562892 | 1.510512 | 2.144131 | 0 | 0 | 0 | 0 | 8 | 2 | 0.058115 |
|  |  | 2.375261 | 4.531537 | 0.71471 | 0 | 0 | 0 | 0 | 9 | 2 | 0.148222 |
| PPS_4879 | Function unknown | 1.187631 | 2.265768 | 1.429421 | 0 | 0 | 0 | 0 | 6 | 3 | 0.037992 |
| PPS_3827 | Transcription | 1.187631 | 2.265768 | 1.429421 | 0 | 0 | 0 | 0 | 6 | 4 | 0.037992 |
| PPS_3821 |  | 1.187631 | 2.265768 | 1.429421 | 0 | 0 | 0 | 0 | 6 | 2 | 0.037992 |
|  |  | 2.375261 | 3.776281 | 2.858842 | 0 | 0 | 0 | 0 | 11 | 2 | 0.018203 |
| PPS_2174 | Amino acid transport and metabolism | 1.187631 | 3.021025 | 2.144131 | 0 | 0 | 0 | 0 | 8 | 4 | 0.057195 |
| PPS_3292 |  | 4.750523 | 4.531537 | 2.858842 | 0 | 0 | 0 | 0 | 14 | 3 | 0.021105 |
| PPS_0542 |  | 1.187631 | 1.510512 | 1.429421 | 0 | 0 | 0 | 0 | 5 | 2 | 0.004932 |
| PPS_1814 | General function prediction only | 1.187631 | 1.510512 | 1.429421 | 0 | 0 | 0 | 0 | 5 | 2 | 0.004932 |
| PPS_2072 | Secondary metabolites biosynthesis | 4.750523 | 0.755256 | 0.71471 | 0 | 0 | 0 | 0 | 6 | 5 | 0.261483 |
| PPS_4957 | General function prediction only | 5.938153 | 1.510512 | 1.429421 | 0 | 0 | 0 | 0 | 9 | 4 | 0.185324 |
| PPS_0310 | Cell motility and secretion | 2.375261 | 3.776281 | 2.144131 | 0 | 0 | 0 | 0 | 10 | 3 | 0.032362 |
| PPS_4432 | Inorganic ion transport and metabolism | 4.750523 | 0.755256 | 0.71471 | 0 | 0 | 0 | 0 | 6 | 5 | 0.261483 |
| PPS_4808 | Carbohydrate transport and metabolism | 2.375261 | 1.510512 | 1.429421 | 0 | 0 | 0 | 0 | 6 | 2 | 0.027966 |
| PPS_4784 | Cell envelope biogenesis | 2.375261 | 2.265768 | 0 | 0 | 0 | 0 | 0 | 5 | 2 | 0.183731 |
| PPS_2390 | Cell motility and secretion | 2.375261 | 5.286793 | 2.144131 | 0 | 0 | 0 | 0 | 12 | 4 | 0.083846 |
| PPS_3106 | Inorganic ion transport and metabolism | 1.187631 | 1.510512 | 1.429421 | 0 | 0 | 0 | 0 | 5 | 3 | 0.004932 |
| PPS_4741 | General function prediction only | 4.750523 | 1.510512 | 4.288263 | 0 | 0 | 0 | 0 | 12 | 3 | 0.073749 |
| PPS_3568 |  | 0 | 2.265768 | 2.144131 | 0 | 0 | 0 | 0 | 6 | 2 | 0.183814 |
| PPS_0618 | Energy production and conversion | 1.187631 | 1.510512 | 1.429421 | 0 | 0 | 0 | 0 | 5 | 3 | 0.004932 |
| PPS_0288 | Amino acid transport and metabolism | 2.375261 | 3.776281 | 1.429421 | 0 | 0 | 0 | 0 | 9 | 4 | 0.065688 |
| PPS_3868 | Cell motility and secretion | 3.562892 | 3.776281 | 0.71471 | 0 | 0 | 0 | 0 | 9 | 2 | 0.112737 |
| PPS_5180 | Carbohydrate transport and metabolism | 1.187631 | 2.265768 | 1.429421 | 0 | 0 | 0 | 0 | 6 | 2 | 0.037992 |
| PPS_3052 | Cell motility and secretion | 2.375261 | 4.531537 | 0.71471 | 0 | 0 | 0 | 0 | 9 | 2 | 0.148222 |
| PPS_5003 | Energy production and conversion | 2.375261 | 3.021025 | 1.429421 | 0 | 0 | 0 | 0 | 8 | 3 | 0.038872 |
| PPS_1029 | Amino acid transport and metabolism | 1.187631 | 0 | 2.858842 | 0 | 0 | 0 | 0 | 5 | 3 | 0.245333 |
| PPS_3238 | Amino acid transport and metabolism | 4.750523 | 1.510512 | 0 | 0 | 0 | 0 | 0 | 6 | 2 | 0.274846 |
| PPS_2222 | Energy production and conversion | 7.125784 | 3.776281 | 5.002973 | 0 | 0 | 0 | 0 | 18 | 3 | 0.032409 |
| PPS_2217 | Amino acid transport and metabolism | 4.750523 | 2.265768 | 5.717683 | 0 | 0 | 0 | 0 | 15 | 4 | 0.053959 |
| PPS_1460 | Cell motility and secretion | 3.562892 | 3.776281 | 1.429421 | 0 | 0 | 0 | 0 | 10 | 3 | 0.059871 |
| PPS_2922 | Energy production and conversion | 4.750523 | 5.286793 | 11.43537 | 0 | 0 | 0 | 0 | 27 | 7 | 0.079244 |
| PPS_2924 |  | 3.562892 | 1.510512 | 0.71471 | 0 | 0 | 0 | 0 | 6 | 3 | 0.150824 |
| PPS_2925 | Amino acid transport and metabolism | 3.562892 | 1.510512 | 1.429421 | 0 | 0 | 0 | 0 | 7 | 2 | 0.089933 |
| PPS_0236 | Coenzyme metabolism | 1.187631 | 0.755256 | 2.144131 | 0 | 0 | 0 | 0 | 5 | 3 | 0.079989 |
| PPS_3535 | General function prediction only | 2.375261 | 0.755256 | 1.429421 | 0 | 0 | 0 | 0 | 5 | 2 | 0.083725 |
| PPS_1042 | Signal transduction mechanisms | 2.375261 | 1.510512 | 0.71471 | 0 | 0 | 0 | 0 | 5 | 3 | 0.085428 |
| PPS_1044 | Carbohydrate transport and metabolism | 4.750523 | 7.552562 | 3.573552 | 0 | 0 | 0 | 0 | 19 | 6 | 0.046301 |
| PPS_4178 | Function unknown | 2.375261 | 3.021025 | 1.429421 | 0 | 0 | 0 | 0 | 8 | 3 | 0.038872 |
| PPS_4283 | General function prediction only | 0 | 4.531537 | 2.144131 | 0 | 0 | 0 | 0 | 9 | 4 | 0.231189 |
| PPS_1198 | Acyl carrier protein | 0 | 1.510512 | 2.144131 | 0 | 0 | 0 | 0 | 5 | 2 | 0.195505 |
| PPS_2750 | Carbohydrate transport and metabolism | 4.750523 | 0.755256 | 0.71471 | 0 | 0 | 0 | 0 | 6 | 3 | 0.261483 |
| PPS_0353 | Amino acid transport and metabolism | 1.187631 | 1.510512 | 3.573552 | 0 | 0 | 0 | 0 | 8 | 2 | 0.107562 |
| PPS_0374 | General function prediction only | 0 | 3.776281 | 0.71471 | 0 | 0 | 0 | 0 | 6 | 2 | 0.325355 |
| PPS_4015 | Function unknown | 0 | 4.531537 | 0.71471 | 0 | 0 | 0 | 0 | 7 | 3 | 0.339752 |
