# Supplementary material for: Systematic Unraveling of the Unsolved Pathway of Nicotine Degradation in Pseudomonas
Source: PLoS Genet. 2013 Oct 24;9(10):e1003923. doi: 10.1371/journal.pgen.1003923 (PMC3812094; doi:10.1371/journal.pgen.1003923)
Supplement: Table S2 — Abundance of differential expression protein components involved in central energy metabolism in P. putida S16 cells grown on nicotine and glycerol. (DOCX) [file pgen.1003923.s007.docx]

Table S2. Abundance of differential expression protein components involved in central energy metabolism in *P. putida* S16 cells grown on nicotine and glycerol.

| **NCBI database accession no.** | **KEG** | **Protein annotation** | gs16-1 | | gs16-2 | | gs16-3 | ns16-1 | ns16-2 | ns16-3 |
| --- | --- | --- | --- | --- | --- | --- | --- | --- | --- | --- |
| PPS_1707 | Transcription | TetR family transcriptional regulator | | 0 | | 0 | 0 | 3.12525 | 3.468165 | 1.113377 |
| PPS_4075 |  | Porin | | 0 | | 0 | 0 | 2.0835 | 1.734082 | 2.226755 |
| PPS_4076 | Carbohydrate transport and metabolism | major facilitator superfamily metabolite/H(+) symporter | | 0 | | 0 | 0 | 3.12525 | 8.670412 | 3.340132 |
| PPS_4078 | Energy production and conversion | aldehyde oxidAse and xanthine dehydrogenase (*spmA*) | | 0 | | 0 | 0 | 48.96225 | 34.68165 | 36.74145 |
| PPS_4079 | Energy production and conversion | aldehyde dehydrogenase **(***adh***)** | | 0 | | 0 | 0 | 66.672 | 50.28839 | 62.34913 |
| PPS_4080 | Amino acid transport and metabolism | amine oxidase | | 0 | | 0 | 0 | 140.6363 | 97.10861 | 121.3581 |
| PPS_1542 | Cell division and chromosome partitioning | Maf-like protein | | 0 | | 0 | 0 | 1.04175 | 1.734082 | 3.340132 |
| PPS_3077 | Cell envelope biogenesis | RND efflux system outer membrane lipoprotein | | 0 | | 0 | 0 | 4.167 | 3.468165 | 1.113377 |
| PPS_1628 | Amino acid transport and metabolism | dihydrodipicolinate synthetase | | 0 | | 0 | 0 | 3.12525 | 3.468165 | 0 |
| PPS_0750 | Energy production and conversion | alcohol dehydrogenase | | 0 | | 0 | 0 | 4.167 | 3.468165 | 2.226755 |
| PPS_0749 | Energy production and conversion | xenobiotic reductase A | | 0 | | 0 | 0 | 9.37575 | 6.93633 | 3.340132 |
| PPS_3898 | Lipid metabolism | dehydratase | | 0 | | 0 | 0 | 4.167 | 0 | 3.340132 |
| PPS_4740 | Cell envelope biogenesis | *N*-acetylmuramoyl-L-alanine amidase | | 0 | | 0 | 0 | 1.04175 | 3.468165 | 3.340132 |
| PPS_3778 | Cell motility and secretion | flagellar cap protein FliD | | 0 | | 0 | 0 | 3.12525 | 0 | 4.453509 |
| PPS_4370 | General function prediction only | transport-associated | | 0 | | 0 | 0 | 1.04175 | 3.468165 | 2.226755 |
| PPS_3191 | Amino acid transport and metabolism | conserved hypothetical protein | | 0 | | 0 | 0 | 3.12525 | 3.468165 | 2.226755 |
| PPS_3192 | Energy production and conversion | putative acyl-CoA synthetase | | 0 | | 0 | 0 | 2.0835 | 3.468165 | 2.226755 |
| PPS_3194 | Lipid metabolism | enoyl-CoA hydratase/isomerase | | 0 | | 0 | 0 | 4.167 | 0 | 3.340132 |
| PPS_3960 | Coenzyme metabolism | molybdenum cofactor biosynthesis protein B | | 0 | | 0 | 0 | 11.45925 | 10.40449 | 7.793641 |
| PPS_1564 | Defense mechanisms | secretion protein HlyD family protein | | 0 | | 0 | 0 | 3.12525 | 0 | 3.340132 |
| PPS_4047 | Intracellular trafficking and secretion | putative Sec-independent protein translocase protein | | 0 | | 0 | 0 | 6.2505 | 6.93633 | 8.907018 |
| PPS_4050 | Posttranslational modification | cytochrome c-type biogenesis protein | | 0 | | 0 | 0 | 7.29225 | 6.93633 | 10.0204 |
| PPS_4052 | Posttranslational modification | thiol:disulfide interchange protein DsbE | | 0 | | 0 | 0 | 2.0835 | 10.40449 | 4.453509 |
| PPS_4053 | Posttranslational modification | cytochrome c-type biogenesis protein CcmF | | 0 | | 0 | 0 | 3.12525 | 6.93633 | 7.793641 |
| PPS_4081 | Amino acid transport and metabolism | amine oxidase | | 0 | | 0 | 0.71471 | 120.843 | 46.82022 | 73.4829 |
| PPS_4077 | Energy production and conversion | ferredoxin:(2Fe-2S)-binding:carbon monoxide dehydrogenase subunit G (SpmC) | | 0 | | 0.755256 | 0 | 34.37775 | 55.49064 | 30.06119 |
| PPS_3300 | Posttranslational modification | alkyl hydroperoxide reductase subunit F | | 0 | | 0 | 0.71471 | 5.20875 | 6.93633 | 11.13377 |
| PPS_2020 | Amino acid transport and metabolism | L-asparaginase type II | | 0 | | 0 | 0.71471 | 7.29225 | 3.468165 | 4.453509 |
| PPS_4429 | Energy production and conversion | D-isomer specific 2-hydroxyacid dehydrogenase, NAD-binding | | 0 | | 0.755256 | 0 | 6.2505 | 0 | 4.453509 |
| PPS_4061  PPS_0380  PPS_0381 | Coenzyme metabolism  Function unknown  DNA replication | para-nitrophenol 4-monooxygenase (HspB)  6-hydroxy-3-succinoylpyridine hydroxylase (HspA)  nicotine oxidoreductase (NicA1) | | 8.313414  2.375261  1.187631 | | 18.8814  1.510512  0 | 12.86479  0.71471  0.71471 | 156.2625  1.04175  3.12525 | 171.6742  0  5.202247 | 144.739  0  4.962766511 |
