# Supplementary material for: Systematic Unraveling of the Unsolved Pathway of Nicotine Degradation in Pseudomonas
Source: PLoS Genet. 2013 Oct 24;9(10):e1003923. doi: 10.1371/journal.pgen.1003923 (PMC3812094; doi:10.1371/journal.pgen.1003923)
Supplement: Table S3 — Abundance of differential expression protein components involved in central energy metabolism in P. putida S16 cells grown on nicotine and glycerol. (DOC) [file pgen.1003923.s008.doc]

Table S3. Abundance of differential expression protein components involved in central energy metabolism in *P. putida* S16 cells grown on nicotine and glycerol.

| **NCBI database accession no.** | **KEG** | **Protein annotation** | gs16-1 | gs16-2 | gs16-3 | | ns16-1 | ns16-2 | ns16-3 |
| --- | --- | --- | --- | --- | --- | --- | --- | --- | --- |
| PPS_1051 | Carbohydrate transport and metabolism | glucose-6-phosphate 1-dehydrogenase | 3.562892 | 4.531537 | 5.717683 | 0 | | 0 | 1.113377 |
| PPS_0065 |  | conserved hypothetical protein | 5.938153 | 3.776281 | 4.288263 | 1.04175 | | 0 | 0 |
| PPS_1040 | Carbohydrate transport and metabolism | glucokinase | 4.750523 | 6.797305 | 6.432394 | 0 | | 0 | 1.113377 |
| PPS_2748 | Carbohydrate transport and metabolism | glycerate kinase | 13.06394 | 7.552562 | 7.861815 | 0 | | 1.734082 | 0 |
| PPS_4182 | Energy production and conversion | glycerol-3-phosphate dehydrogenase | 41.56707 | 43.0496 | 48.60031 | 0 | | 5.202247 | 1.113377 |
| PPS_1038 | Carbohydrate transport and metabolism | glyceraldehyde-3-phosphate dehydrogenase type I | 83.13414 | 148.7855 | 121.5008 | 3.12525 | | 5.202247 | 2.226755 |
| PPS_4180 | Energy production and conversion | glycerol kinase | 35.62892 | 33.98653 | 35.73552 | 3.12525 | | 0 | 0 |
| PPS_2226 | Carbohydrate transport and metabolism | putative quinoprotein ethanol dehydrogenase | 46.31759 | 51.35742 | 46.45618 | 4.167 | | 0 | 0 |
| PPS_1052 | Carbohydrate transport and metabolism | 6-phosphogluconolactonase | 14.25157 | 19.63666 | 12.15008 | 1.04175 | | 0 | 0 |
| PPS_0005 | General function prediction only | tRNA modification GTPase TrmE | 5.938153 | 1.510512 | 1.429421 | 0 | | 0 | 0 |
| PPS_1694 | General function prediction only | MOSC domain-containing protein | 3.562892 | 2.265768 | 2.144131 | 0 | | 0 | 0 |
| PPS_3407 | Amino acid transport and metabolism | conserved hypothetical protein | 1.187631 | 0.755256 | 2.144131 | 0 | | 0 | 0 |
| PPS_4110 | Secondary metabolites biosynthesis | short-chain dehydrogenase/reductase SDR | 1.187631 | 2.265768 | 0.71471 | 0 | | 0 | 0 |
| PPS_0175 |  | conserved hypothetical protein | 2.375261 | 0.755256 | 1.429421 | 0 | | 0 | 0 |
| PPS_3737 | Coenzyme metabolism | putative Pyrroloquinoline quinone (Coenzyme PQQ) biosynthesis protein C | 8.313414 | 4.531537 | 3.573552 | 0 | | 0 | 0 |
| PPS_3734 | Secondary metabolites biosynthesis | hypothetical protein | 5.938153 | 6.797305 | 5.717683 | 0 | | 0 | 0 |
| PPS_3378 | Energy production and conversion | malate synthase G | 0 | 3.776281 | 2.858842 | 0 | | 0 | 0 |
| PPS_2801 | Cell envelope biogenesis | lipopolysaccharide biosynthesis protein | 2.375261 | 3.776281 | 0.71471 | 0 | | 0 | 0 |
| PPS_2021 | Carbohydrate transport and metabolism | monosaccharide-transporting ATPase | 3.562892 | 0.755256 | 0.71471 | 0 | | 0 | 0 |
| PPS_5045 | Amino acid transport and metabolism | glycine dehydrogenase | 1.187631 | 2.265768 | 0.71471 | 0 | | 0 | 0 |
| PPS_5023 | Inorganic ion transport and metabolism | sulfate ABC transporter, periplasmic sulfate-binding protein | 0 | 3.021025 | 0.71471 | 0 | | 0 | 0 |
| PPS_1535 | Cell envelope biogenesis | 3-deoxy-manno-octulosonate cytidylyltransferase | 3.562892 | 1.510512 | 2.144131 | 0 | | 0 | 0 |
|  |  |  | 2.375261 | 4.531537 | 0.71471 | 0 | | 0 | 0 |
| PPS_4879 | Function unknown | conserved hypothetical protein | 1.187631 | 2.265768 | 1.429421 | 0 | | 0 | 0 |
| PPS_3827 | Transcription | AraC family transcriptional regulator | 1.187631 | 2.265768 | 1.429421 | 0 | | 0 | 0 |
| PPS_3821 |  | conserved hypothetical protein | 1.187631 | 2.265768 | 1.429421 | 0 | | 0 | 0 |
|  |  |  | 2.375261 | 3.776281 | 2.858842 | 0 | | 0 | 0 |
| PPS_2174 | Amino acid transport and metabolism | dihydrodipicolinate synthase | 1.187631 | 3.021025 | 2.144131 | 0 | | 0 | 0 |
| PPS_3292 |  | AraC family transcriptional regulator | 4.750523 | 4.531537 | 2.858842 | 0 | | 0 | 0 |
| PPS_0542 |  | Fis family GAF modulated sigma54 specific transcriptional regulator | 1.187631 | 1.510512 | 1.429421 | 0 | | 0 | 0 |
| PPS_1814 | General function prediction only | conserved hypothetical protein | 1.187631 | 1.510512 | 1.429421 | 0 | | 0 | 0 |
| PPS_2072 | Secondary metabolites biosynthesis | short-chain dehydrogenase/reductase SDR | 4.750523 | 0.755256 | 0.71471 | 0 | | 0 | 0 |
| PPS_4957 | General function prediction only | peptidase M16 domain-containing protein | 5.938153 | 1.510512 | 1.429421 | 0 | | 0 | 0 |
| PPS_0310 | Cell motility and secretion | methyl-accepting chemotaxis sensory transducer | 2.375261 | 3.776281 | 2.144131 | 0 | | 0 | 0 |
| PPS_4432 | Inorganic ion transport and metabolism | TonB-dependent siderophore receptor | 4.750523 | 0.755256 | 0.71471 | 0 | | 0 | 0 |
| PPS_4808 | Carbohydrate transport and metabolism | D-erythrose-4-phosphate dehydrogenase | 2.375261 | 1.510512 | 1.429421 | 0 | | 0 | 0 |
| PPS_4784 | Cell envelope biogenesis | conserved hypothetical protein | 2.375261 | 2.265768 | 0 | 0 | | 0 | 0 |
| PPS_2390 | Cell motility and secretion | methyl-accepting chemotaxis sensory transducer | 2.375261 | 5.286793 | 2.144131 | 0 | | 0 | 0 |
| PPS_3106 | Inorganic ion transport and metabolism | TonB-dependent siderophore receptor | 1.187631 | 1.510512 | 1.429421 | 0 | | 0 | 0 |
| PPS_4741 | General function prediction only | conserved hypothetical protein | 4.750523 | 1.510512 | 4.288263 | 0 | | 0 | 0 |
| PPS_3568 |  | conserved hypothetical protein | 0 | 2.265768 | 2.144131 | 0 | | 0 | 0 |
| PPS_0618 | Energy production and conversion | FAD-dependent pyridine nucleotide-disulphide oxidoreductase | 1.187631 | 1.510512 | 1.429421 | 0 | | 0 | 0 |
| PPS_0288 | Amino acid transport and metabolism | choline ABC transporter ATP-binding protein | 2.375261 | 3.776281 | 1.429421 | 0 | | 0 | 0 |
| PPS_3868 | Cell motility and secretion | methyl-accepting chemotaxis sensory transducer with Pas/Pac sensor | 3.562892 | 3.776281 | 0.71471 | 0 | | 0 | 0 |
| PPS_5180 | Carbohydrate transport and metabolism | aldose 1-epimerase | 1.187631 | 2.265768 | 1.429421 | 0 | | 0 | 0 |
| PPS_3052 | Cell motility and secretion | methyl-accepting chemotaxis sensory transducer | 2.375261 | 4.531537 | 0.71471 | 0 | | 0 | 0 |
| PPS_5003 | Energy production and conversion | FAD linked oxidase domain-containing protein | 2.375261 | 3.021025 | 1.429421 | 0 | | 0 | 0 |
| PPS_1029 | Amino acid transport and metabolism | arginine/ornithine antiporter | 1.187631 | 0 | 2.858842 | 0 | | 0 | 0 |
| PPS_3238 | Amino acid transport and metabolism | shikimate 5-dehydrogenase | 4.750523 | 1.510512 | 0 | 0 | | 0 | 0 |
| PPS_2222 | Energy production and conversion | cytochrome c class I | 7.125784 | 3.776281 | 5.002973 | 0 | | 0 | 0 |
| PPS_2217 | Amino acid transport and metabolism | ABC-type branched-chain amino acid transport systems periplasmic compoment-like protein | 4.750523 | 2.265768 | 5.717683 | 0 | | 0 | 0 |
| PPS_1460 | Cell motility and secretion | methyl-accepting chemotaxis sensory transducer | 3.562892 | 3.776281 | 1.429421 | 0 | | 0 | 0 |
| PPS_2922 | Energy production and conversion | aldehyde dehydrogenase | 4.750523 | 5.286793 | 11.43537 | 0 | | 0 | 0 |
| PPS_2924 |  | conserved hypothetical protein | 3.562892 | 1.510512 | 0.71471 | 0 | | 0 | 0 |
| PPS_2925 | Amino acid transport and metabolism | threonine dehydratase | 3.562892 | 1.510512 | 1.429421 | 0 | | 0 | 0 |
| PPS_0236 | Coenzyme metabolism | glutamate-cysteine ligase | 1.187631 | 0.755256 | 2.144131 | 0 | | 0 | 0 |
| PPS_3535 | General function prediction only | NADPH-dependent FMN reductase | 2.375261 | 0.755256 | 1.429421 | 0 | | 0 | 0 |
| PPS_1042 | Signal transduction mechanisms | integral membrane sensor signal transduction histidine kinase | 2.375261 | 1.510512 | 0.71471 | 0 | | 0 | 0 |
| PPS_1044 | Carbohydrate transport and metabolism | extracellular solute-binding protein | 4.750523 | 7.552562 | 3.573552 | 0 | | 0 | 0 |
| PPS_4178 | Function unknown | YbaK/EbsC protein | 2.375261 | 3.021025 | 1.429421 | 0 | | 0 | 0 |
| PPS_4283 | General function prediction only | conserved hypothetical protein | 0 | 4.531537 | 2.144131 | 0 | | 0 | 0 |
| PPS_1198 | acyl carrier protein | UDP-N-acetylglucosamine acyltransferase | 0 | 1.510512 | 2.144131 | 0 | | 0 | 0 |
| PPS_2750 | Carbohydrate transport and metabolism | major facilitator transporter | 4.750523 | 0.755256 | 0.71471 | 0 | | 0 | 0 |
| PPS_0353 | Amino acid transport and metabolism | ACT domain-containing protein | 1.187631 | 1.510512 | 3.573552 | 0 | | 0 | 0 |
| PPS_0374 | General function prediction only | pyrroloquinoline quinone biosynthesis protein PqqB | 0 | 3.776281 | 0.71471 | 0 | | 0 | 0 |
| PPS_4015 | Function unknown | conserved hypothetical protein | 0 | 4.531537 | 0.71471 | 0 | | 0 | 0 |
