# Supplementary material for: Systematic Unraveling of the Unsolved Pathway of Nicotine Degradation in Pseudomonas
Source: PLoS Genet. 2013 Oct 24;9(10):e1003923. doi: 10.1371/journal.pgen.1003923 (PMC3812094; doi:10.1371/journal.pgen.1003923)
Supplement: Table S4 — Primers used for gene expression analysis (RT-qPCR). (DOC) [file pgen.1003923.s009.doc]

Table S4. Primers used for gene expression analysis (RT-qPCR)

| Target gene | Primer sequence | Primer length (bases) | GC content (%) | Melt temp (°C) | Amplicon length (bases) |
| --- | --- | --- | --- | --- | --- |
| 16S rRNA | GAACGCTAATACCGCATACGTCC  ATCATCCTCTCAGACCAGTTAC | 23  22 | 52.2  45.5 | 63  52.4 | 144 |
| *spmA*  PPS_4078 | CCTATTCGCACTGGTATGG  CTCACGCCTATCCTCAAC | 19  18 | 52.6  55.6 | 51.9  51 | 180 |
| *spmC*  PPS_4077 | AGGAGCGGAGGTAGTTAG  TGACAGTCCAGGTAATTCG | 18  19 | 55.6  47.4 | 51  50.9 | 197 |
| *porin*  PPS_4075 | TGGATGCTTATGCTGGTCTC  GTGACGGTTGTCTCTGAATATC | 20  22 | 50  45.5 | 54.3  54.2 | 121 |
| *hspB*  PPS_4061 | AGTAGTCGCCTTCTCACCATATAG  ACCGTCGTGTATGACCTGATTC | 24  22 | 45.8  50 | 57.1  58.9 | 184 |
| *mfs*  PPS_4076 | CGACGATATAGACCACCT  GTAGTCATTGCCATCACC | 18  18 | 50  50 | 57.6  57.6 | 85 |
| *sapd*  PPS_4079 | CAGAGATTGCCGTTAGGA  TTACCGAAGAGATGGGAG | 18  18 | 50  50 | 57.6  57.6 | 90 |
| *pnao*  PPS_4080 | ACTTGATGCCCTACCTCT  CACTTACCGTCCTAACCAG | 18  19 | 50  52.63 | 57.6  60.2 | 150 |
| *nicA2*  PPS_4081 | CATCGCTCGCAAAGAAAC  ACCAAGAACCTCAACACC | 18  18 | 50  50 | 57.6  57.6 | 91 |
