# Supplementary material for: Systematic Unraveling of the Unsolved Pathway of Nicotine Degradation in Pseudomonas
Source: PLoS Genet. 2013 Oct 24;9(10):e1003923. doi: 10.1371/journal.pgen.1003923 (PMC3812094; doi:10.1371/journal.pgen.1003923)
Supplement: Table S5 — The spm genes and their products in P. putida S16. (DOC) [file pgen.1003923.s010.doc]

Table S5. *spm* genes and their products in *P. putida* S16

|  |  | Related gene products | | | | |
| --- | --- | --- | --- | --- | --- | --- |
| Gene (PP no.) | Gene product (aa) | Name (aa) | Function | Organism | % identity | Accession no. |
| *spmA* (PPS_4078) | SpmA (801) | QorL (788) | quinoline 2-oxidoreductase (large subunit) | *Pseudomonas putida* 86 | 36 | 1T3Q-B |
|  |  | NdhL (816) | nicotine dehydrogenase large subunit | *Arthrobacter nicotinovorans* | 34 | AAK64263 |
| *spmB* | SpmB (278) | QorM (288) | quinoline 2-oxidoreductase (medium subunit) | *Pseudomonas putida* 86 | 34 | 1T3Q-C |
|  |  | NdhM (283) | nicotine dehydrogenase medium subunit | *Arthrobacter nicotinovorans* | 33 | AAK64243 |
| *spmC* (PPS_4077) | SpmC (391) | QorS (168) | quinoline 2-oxidoreductase (small subunit) | *Pseudomonas putida* 86 | 50 | 1T3Q-D |
|  |  | NdhS (165) | nicotine dehydrogenase small subunit | *Arthrobacter nicotinovorans* | 42 | AAK64244 |
