# Supplementary material for: Systematic Unraveling of the Unsolved Pathway of Nicotine Degradation in Pseudomonas
Source: PLoS Genet. 2013 Oct 24;9(10):e1003923. doi: 10.1371/journal.pgen.1003923 (PMC3812094; doi:10.1371/journal.pgen.1003923)
Supplement: Table S6 — Primers of genes knocked out. (DOCX) [file pgen.1003923.s011.docx]

**Table S6. Primers of genes knocked out.**

| Target gene | Primer sequence | Primer length (bases) | GC content (%) | Melt temp (°C) |
| --- | --- | --- | --- | --- |
| *mfs* | ATGGCACGTCGACGATATAGACCACCT  TATGGCTGAATTCGCACCTAAAGGGCG | 27  27 | 51.9  51.9 | 70.0  73.7 |
| *pnao* | GAGTCGACATCCCATATCCACTTTAGC  GCGAATTCTGCCTGTAACGAATACTAC | 27  27 | 48.1  44.4 | 65.8  64.4 |
| *nicA2* | CGGAATTCTGTAGTGAGTTTCAGTGTC  TAGTCGACGTGTAGTGGAAGATCCACT | 27  27 | 44.4  48.1 | 62.9  64.8 |
| *nicA1* | GTCAGAATTCACTCCGCCTGCGTATCAAG | 25 | 52.0 | 67.8 |
|  | CAATGTCGACACTCACCCACTACGCTTCTG | 25 | 56.0 | 67.6 |
