# Supplementary material for: Systematic Unraveling of the Unsolved Pathway of Nicotine Degradation in Pseudomonas
Source: PLoS Genet. 2013 Oct 24;9(10):e1003923. doi: 10.1371/journal.pgen.1003923 (PMC3812094; doi:10.1371/journal.pgen.1003923)
Supplement: Text S1 — Supplementary materials and methods. (DOCX) [file pgen.1003923.s012.docx]

**Supporting information**

**Systematic Unraveling of the Unsolved Pathway of Nicotine Degradation in *Pseudomonas***

Hongzhi Tang, Lijuan Wang^†^, Weiwei Wang^†^, Hao Yu^†^, Kunzhi Zhang, Yuxiang Yao, and Ping Xu^*^

State Key Laboratory of Microbial Metabolism, and School of Life Sciences & Biotechnology, Shanghai Jiao Tong University, Shanghai 200240, People’s Republic of China

^*^ Corresponding author.

Mailing address: State Key Laboratory of Microbial Metabolism, and School of Life Sciences & Biotechnology, Shanghai Jiao Tong University, Shanghai 200240, P. R. China.

E-mail: [pingxu@sjtu.edu.cn](mailto:pingxu@sjtu.edu.cn) (P. Xu).

Tel: +86-21-34206647; Fax: +86-21-34206723.

^†^ These authors contributed equally to this work.

Short Title: Molecular Mechanism of Nicotine Degradation

**Materials and Methods**

**Expression and purification of recombinant nicotine oxidoreductase**. The *nicA2* gene was amplified by PCR from genomic DNA of strain S16 with Prime STAR^TM^ HS DNA polymerase (TaKaRa Co. Ltd., China). Primers were designed as follows, forward: 5′-TGCCATGGCAatgagtgataaaacaaaaac-3′, and reverse: 5′-TAGTCGACCTAGCTTAAGAGCTGCTTAACC-3′. The PCR products were ligated into pET-28a to get pET28a-*nicA2*, and transformed *E. coli* BL21(DE3). Cells were cultured at 37°C in LB medium containing 100 mg l^-1^ kanamycin until an OD_600nm_ of 0.6 reached. Then, IPTG was added to a final concentration of 1 mM and the culture was continuously incubated for h at 30°C. The induced *E. coli* cells were washed and resuspended in binding buffer (20 mM PBS, 100 mM NaCl, 10 mM imidazole, 0.1 mM PMSF, pH 7). This suspension was disrupted by sonication, and the cell debris was removed by centrifugation. The supernatant was applied to a column of Ni sepharose (GE Healthcare), which had been equilibrated with the binding buffer. After a wash with 50 ml of wash buffer (20 mM PBS, 100 mM NaCl, 10 mM imidazole pH 7), His_6_-tagged NicA2 was eluted from the column with elution buffer (20 mM PBS, 100 mM NaCl, 50 mM imidazole pH 7). All subsequent chromatographic steps were performed with a fast protein liquid chromatography system (GE Healthcare) at 4°C.

**Nicotine degradation by resting cells.** Cells were harvested in mid-exponential phase by centrifugation at 6,000 g for 15 min at 4°C, washed three times with PBS buffer. These cells are called resting cells. The degradation experiment was performed in a 30 ml shaking tube containing 5 ml resting cells (OD_600nm_ ~7) resuspended with ddH_2_O and 3 g l^-1^ nicotine, at 30°C with shaking at 150 rpm.
